# Supplementary material for: Clonal hematopoiesis with JAK2V617F promotes pulmonary hypertension with ALK1 upregulation in lung neutrophils
Source: Nat Commun. 2021 Oct 26;12:6177. doi: 10.1038/s41467-021-26435-0 (PMC8548396; doi:10.1038/s41467-021-26435-0)
Supplement: Supplementary file 1 — Supplementary Information [file 41467_2021_26435_MOESM1_ESM.pdf]

# Clonal hematopoiesis with JAK2V617F promotes pulmonary hypertension with ALK1 upregulation in lung neutrophils

Yusuke Kimishima[1], Tomofumi Misaka[1,2], Tetsuro Yokokawa[1,3], Kento Wada[1], Koki Ueda[4], Koichi Sugimoto[1,3], Keiji Minakawa[4], Kazuhiko Nakazato[1], Takafumi Ishida[1], Motohiko Oshima[5], Shuhei Koide[5], Kotaro Shide[6], Kazuya Shimoda[6], Atsushi Iwama[5], Kazuhiko Ikeda[4], Yasuchika Takeishi[1]

[1]Department of Cardiovascular Medicine, Fukushima Medical University, Fukushima, Japan, [2]Department of Advanced Cardiac Therapeutics, Fukushima Medical University, Fukushima, Japan, [3]Department of Pulmonary Hypertension, Fukushima Medical University, Fukushima, Japan, [4]Department of Blood Transfusion and Transplantation Immunology, Fukushima Medical University, Fukushima, Japan, [5]Division of Stem Cell and Molecular Medicine, Center for Stem Cell Biology and Regenerative Medicine, The Institute of Medical Science, The University of Tokyo, Tokyo, Japan, [6]Department of Gastroenterology and Hematology, University of Miyazaki, Miyazaki, Japan.

## Supplementary Information

**Supplementary Figures 1-33**

**Supplementary Tables 1-6**

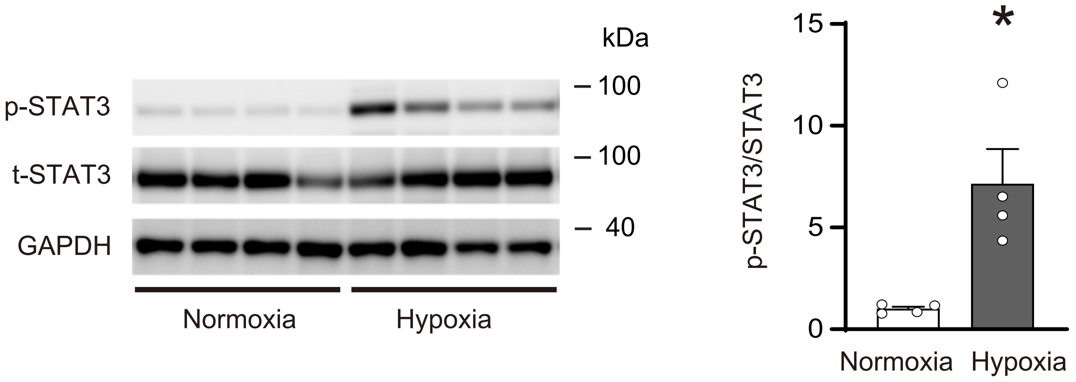


**Supplementary Figure 1. Changes in phosphorylation levels of STAT3 on whole lung homogenates during chronic hypoxia in the wild-type (WT) mice.**

Lung homogenates obtained from the adult WT mice with a C57BL/6J background after normoxia (21% $O_2$) or chronic hypoxia (10% $O_2$) for 3 weeks were analyzed by immunoblotting with anti-phosphorylated-STAT3 and STAT3 antibodies. p-STAT3 and t-STAT3 indicate phosphorylated and total STAT3, respectively. p-STAT3 to t-STAT3 ratios are shown in the bar graph (n = 4 in each group). Data are presented as mean ± SEM. *P = 0.0116 versus the normoxia group by the unpaired Student's t-test (two-sided). Source data are provided as a Source Data file.

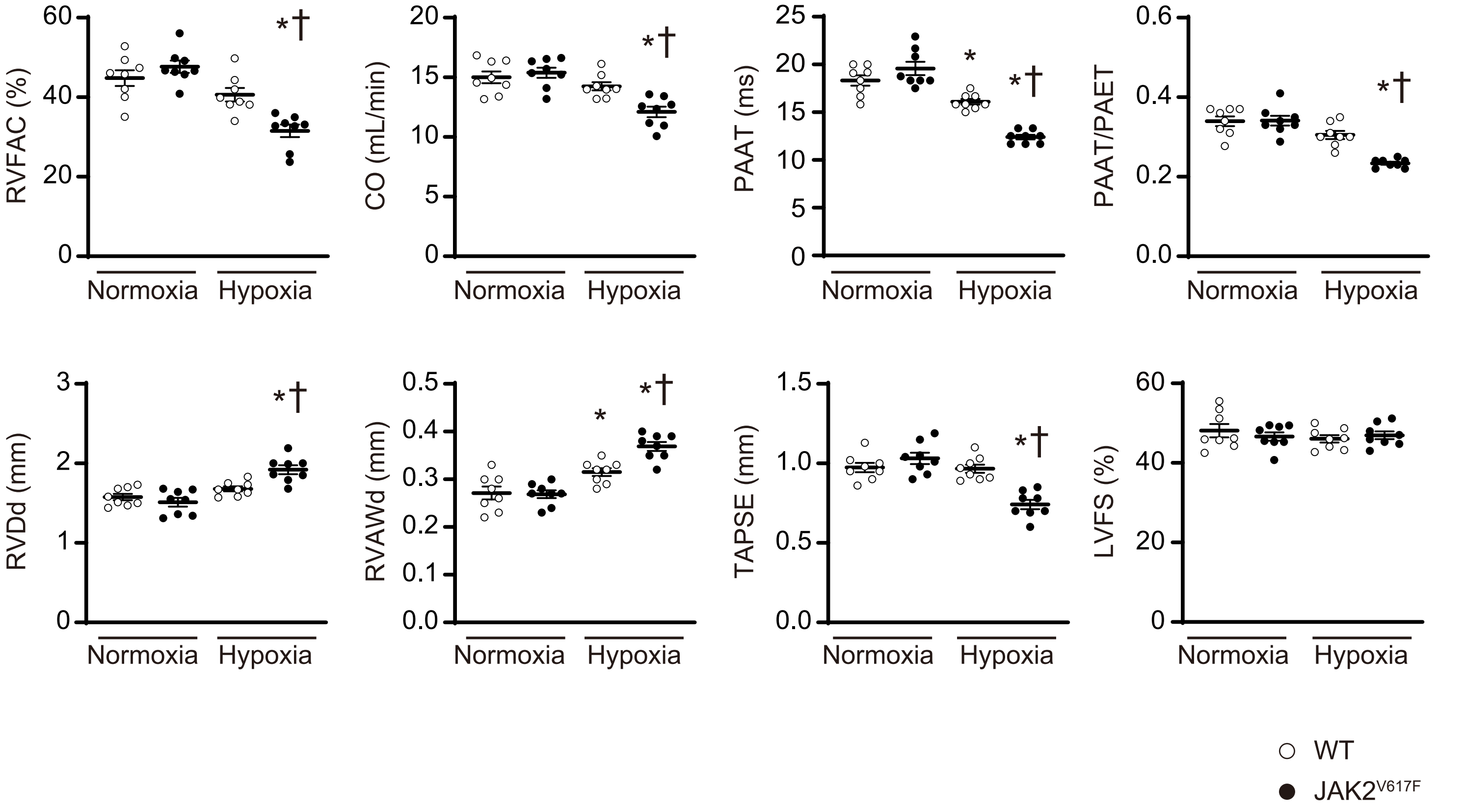


**Supplementary Figure 2. Echocardiographic analysis in JAK2$^{V617F}$ mice after exposure to chronic hypoxia.**

Echocardiography was performed to evaluate pulmonary hemodynamics and cardiac function 2 weeks after normoxia or chronic hypoxia (n = 8 in each group). All data are presented as mean ± SEM. *P < 0.05 versus the corresponding normoxia-exposed group and †P < 0.05 versus the corresponding WT mice by one-way ANOVA with Tukey post-hoc analysis. *P < 0.0001, †P = 0.0039 for RVFAC, *P < 0.0001, †P = 0.0078 for CO, *P = 0.0134 [left], < 0.0001 [right], †P < 0.0001 for PAAT, *P < 0.0001, †P = 0.0002 for PAAT/PAET, *P < 0.0001, †P = 0.0052 for RVDd, *P = 0.0235 [left], < 0.0001 [right], †P = 0.0042 for RVAWd, *P < 0.0001, †P < 0.0001 for TAPSE. RVFAC, right ventricular fractional area change; CO, cardiac output; PAAT, pulmonary artery acceleration time; PAET, pulmonary artery ejection time; RVDd, right ventricular diastolic diameter, RVAWd, right ventricular anterior wall diameter; TAPSE, tricuspid annular plane systolic excursion; LVFS, left ventricular fractional shortening; WT, wild-type mice; JAK2$^{V617F}$, JAK2V617F-expressing transgenic mice. Source data are provided as a Source Data file.

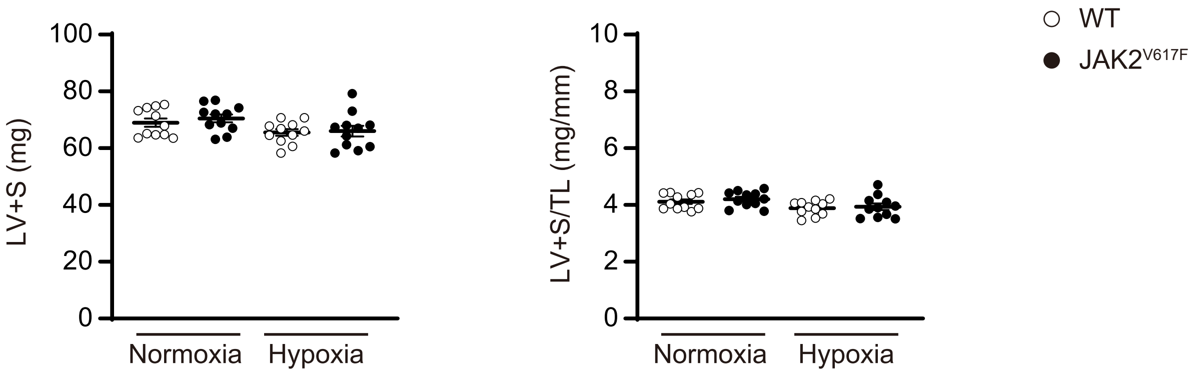


**Supplementary Figure 3. Left ventricular weight in JAK2$^{V617F}$ mice after exposure to chronic hypoxia.**
Left ventricular (LV) weight including septum (S) was measured after exposure to normoxia or chronic hypoxia for 2 weeks (n = 11 in each group). LV+S was normalized by tibia length (TL). All data are presented as mean ± SEM. The statistical comparison was performed by the one-way ANOVA. WT, wild-type mice; JAK2$^{V617F}$, JAK2V617F-expressing transgenic mice. Source data are provided as a Source Data file.

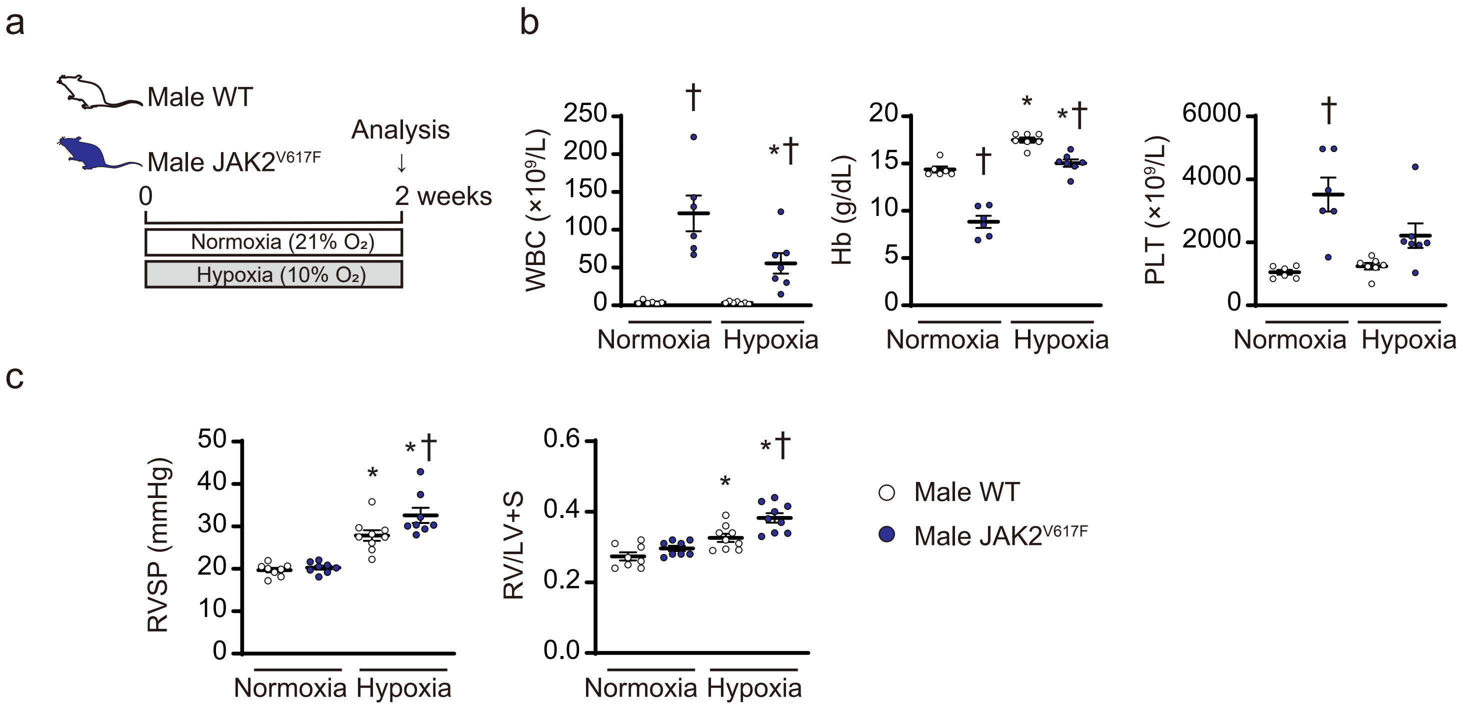


**Supplementary Figure 4. Male JAK2$^{V617F}$ mice also develop pulmonary hypertension in response to exposure to chronic hypoxia.**
**(a)** Experimental design. Male wild-type (WT) mice and male JAK2$^{V617F}$ mice aged between 8 and 10 weeks were exposed to normoxia (21% $O_2$) or hypoxia (10% $O_2$) for 2 weeks. **(b)** Peripheral blood cell counts in WT mice or JAK2$^{V617F}$ mice after normoxia or hypoxia for 2 weeks (n = 6, 6, 7, 7, *P = 0.0088, $^{\dagger}$P < 0.0001 [left], 0.0360 [right] for WBC, n = 6, 6, 7, 7, *P = 0.0002 [left], < 0.0001 [right], $^{\dagger}$P < 0.0001 [left], 0.0015 [right] for Hb, n = 6, 6, 7, 7, $^{\dagger}$P = 0.0003 for PLT). **(c)** Right ventricular systolic pressure (RVSP) and right ventricular hypertrophy determined by the ratio of right ventricle (RV) weight to left ventricle weight plus septum weight (RV/LV+S) (n = 8, 8, 9, 8, *P < 0.0001 [left], < 0.0001 [right], $^{\dagger}$P = 0.0302 for RVSP, n = 8, 8, 9, 8, *P =0.0171 [left], < 0.0001 [right], $^{\dagger}$P = 0.00052 for RV/LV+S). All data are presented as mean ± SEM. *P < 0.05 versus the corresponding normoxia-exposed group and $^{\dagger}$P < 0.05 versus the corresponding WT mice by the one-way ANOVA with Tukey post-hoc analysis. WBC, white blood cell count; Hb, hemoglobin concentration; PLT, platelet count. WT, wild-type mice; JAK2$^{V617F}$, JAK2V617F-expressing transgenic mice. Source data are provided as a Source Data file.

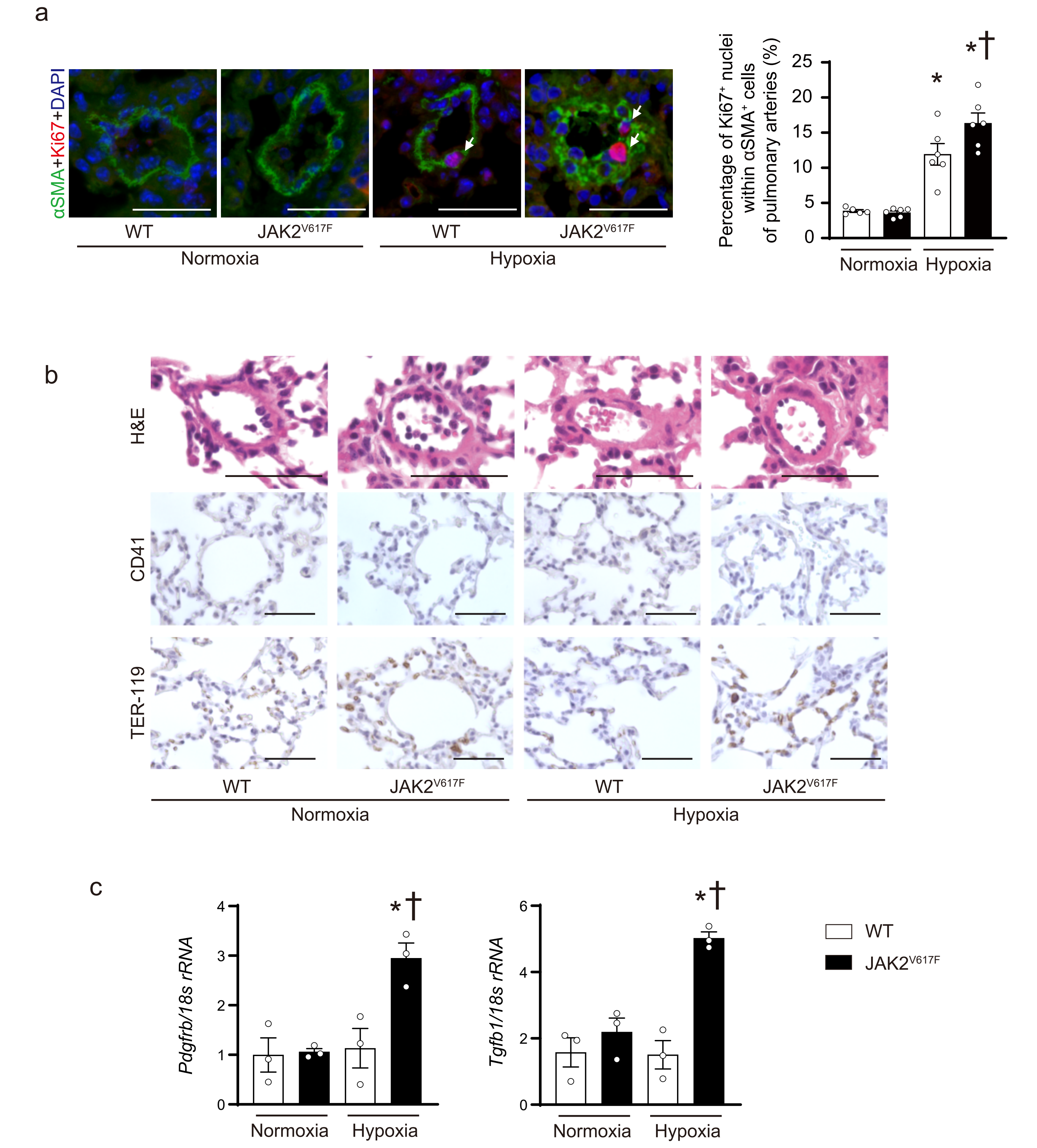


**Supplementary Figure 5. Histological images and mRNA expression of the lung in JAK2$^{V617F}$ mice after exposure to chronic hypoxia.**

**(a)** Left, triple-labeled immunofluorescent staining (αSMA, green; Ki67, red; DAPI, blue) of the lung sections. Right, quantitative analyses of the percentage of Ki67-positive nuclei within αSMA$^{+}$ cells of distal pulmonary arteries with a diameter of 50-100 μm (n = 5, 6, 6, 6, *P = 0.0005 [left], < 0.0001 [right], $^{†}$P = 0.0448). More than 80 αSMA$^{+}$ cells were counted. White arrows indicate Ki67-positive nuclei within αSMA$^{+}$ cells. Scale bars, 50 μm. **(b)** Representative images of the lung sections of H&E staining and immunostaining for CD41 and TER-119 from WT mice and JAK2$^{V617F}$ mice after normoxia and chronic hypoxia. Scale bars, 50 μ m. **(c)** mRNA levels of *Pdgfrb* and*Tgfb1* in the lungs. The *18s rRNA* was used for the normalization. Data are presented as mean ± SEM. The average value for the normoxia-WT mice was set to 1 (n = 3 in each group, *P = 0.0105, $^{†}$P = 0.0132 for *Pdgfrb*, *P = 0.0036, $^{†}$P = 0.0009 for *Tgfb1*). All data are presented as mean ± SEM. $^{*}$P < 0.05 versus the corresponding normoxia-exposed group and $^{†}$P < 0.05 versus the corresponding WT mice by the one-way ANOVA with Tukey post-hoc analysis. WT, wild-type mice; JAK2$^{V617F}$, JAK2V617F-expressing transgenic mice. Source data are provided as a Source Data file.

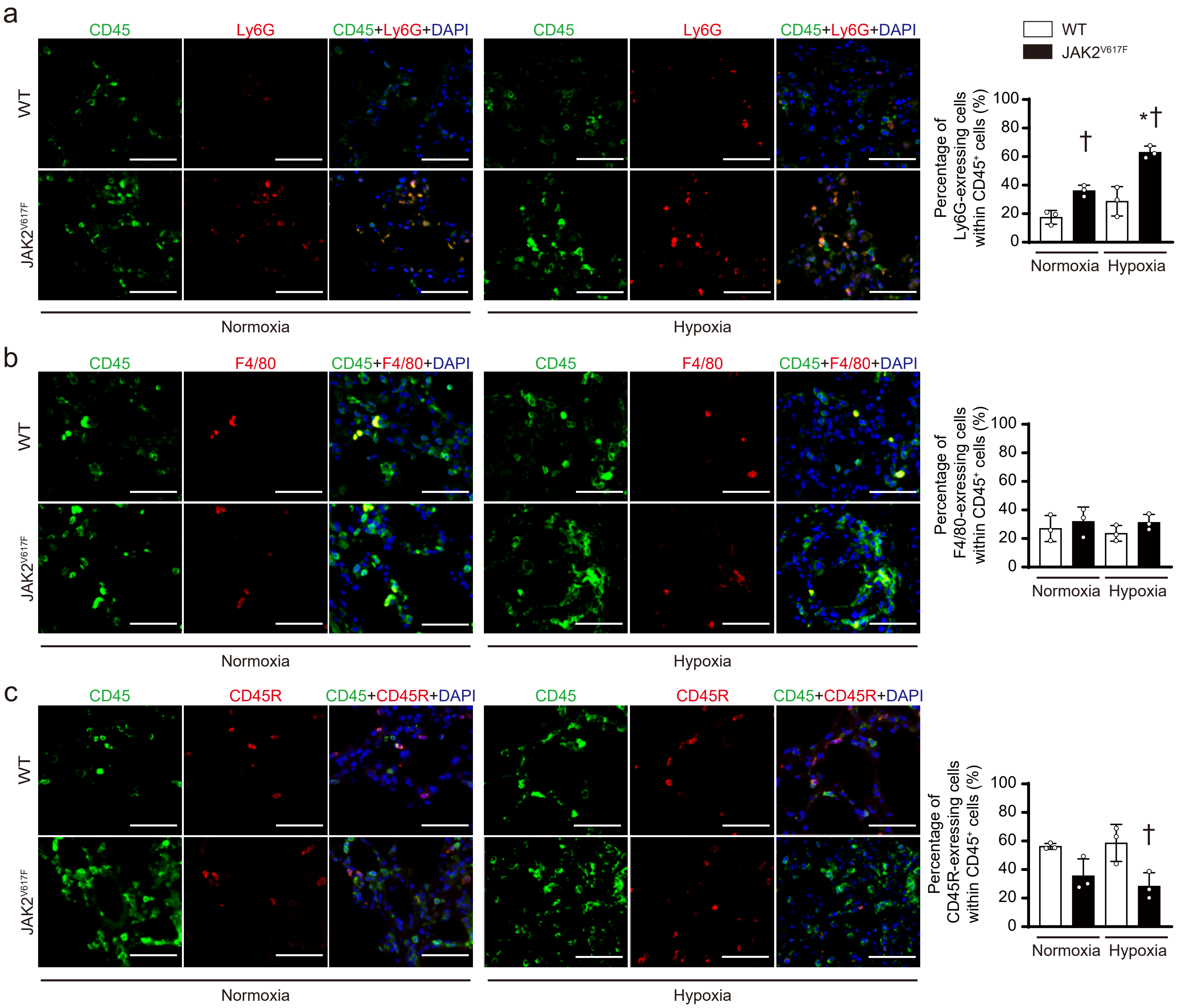


**Supplementary Figure 6. Characterization of the infiltrated leukocytes in the pulmonary arterial regions in JAK2$^{V617F}$ mice after exposure to chronic hypoxia.**

**(a)** Left, triple-labeled immunofluorescent staining (CD45, green; Ly6G, red; DAPI, blue) of the lung sections from WT mice and JAK2$^{V617F}$ mice after normoxia or chronic hypoxia. Right, quantitative analyses of the Ly6G-expressing cells within CD45$^{+}$ cells (n = 3 in each group, *P = 0.0038, $^{†}$P = 0.0297 [left], 0.0008 [right]). **(b)** Left, triple-labeled immunofluorescent staining (CD45, green; F4/80, red; DAPI, blue). Right, quantitative analyses of the F4/80-expressing cells within CD45$^{+}$ cells (n = 3 in each group). **(c)** Left, triple-labeled immunofluorescent staining (CD45, green; CD45R, red; DAPI, blue). Right, quantitative analyses of the CD45R-expressing cells within CD45$^{+}$ cells (n = 3 in each group, $^{†}$P = 0.0250). At least 100 CD45$^{+}$ cells were counted in each. Data are presented as mean ± SEM. $^{*}$P < 0.05 versus the corresponding normoxia-exposed group and $^{†}$P < 0.05 versus the corresponding WT mice by the one-way ANOVA with Tukey post-hoc analysis. Scale bars, 50 μm. WT, wild-type mice; JAK2$^{V617F}$, JAK2V617F-expressing transgenic mice. Source data are provided as a Source Data file.

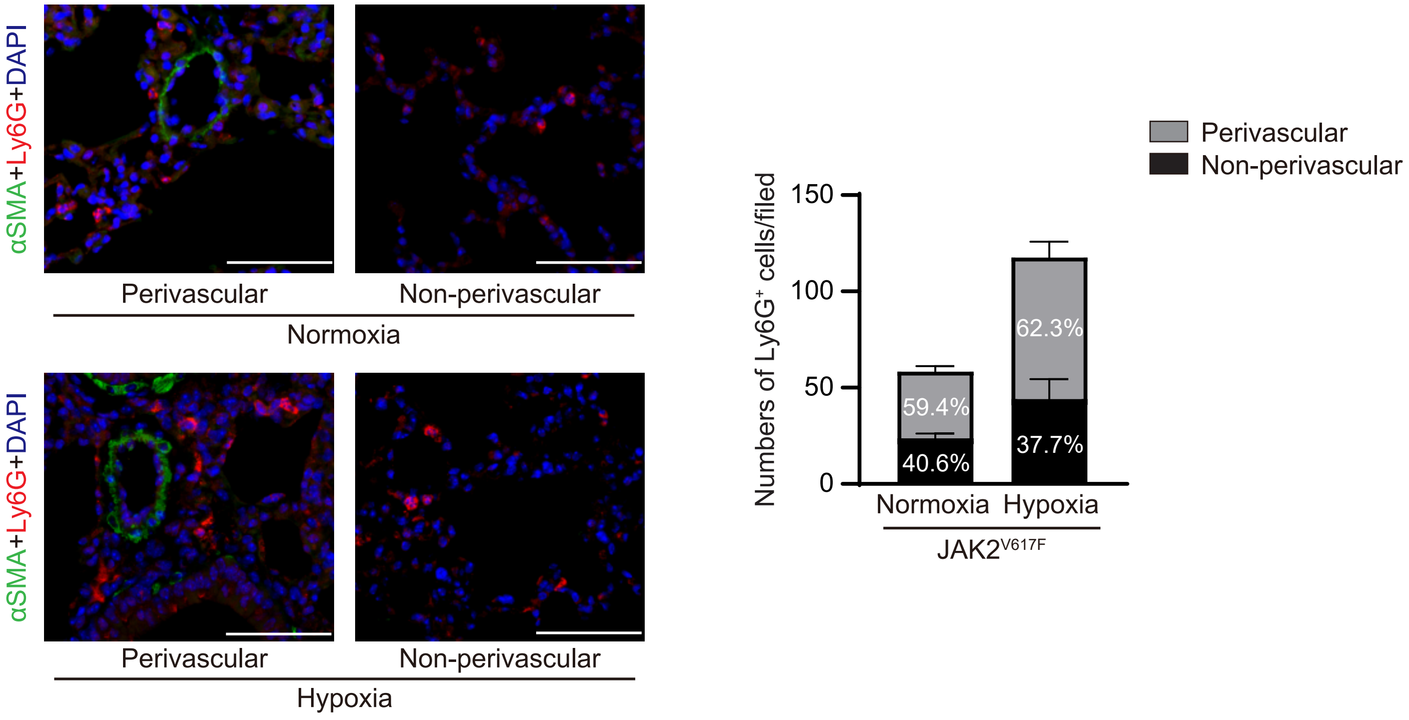


**Supplementary Figure 7. Chronic hypoxia increased Ly6G⁺ neutrophils in perivascular regions as well as non-perivascular regions of the lungs in JAK2$^{V617F}$ mice.**

Left, triple-labeled immunofluorescent staining (αSMA, green; Ly6G, red; DAPI, blue) of the lung sections in JAK2$^{V617F}$ mice. Perivascular regions were determined as the area within 100 μm from distal pulmonary arteries with diameters of 50 μm. Scale bars, 50 μm. Right, quantitative analyses of the numbers of Ly6G$^{+}$ cells in perivascular regions as well as non-perivascular regions (n = 3). More than 10 fields were analyzed in each group. One field was defined as 200 μm x 200 μm. The percentages of the Ly6G$^{+}$ cells in perivascular regions and non-perivascular regions in each group are shown. All data are presented as mean ± SEM. Source data are provided as a Source Data file.

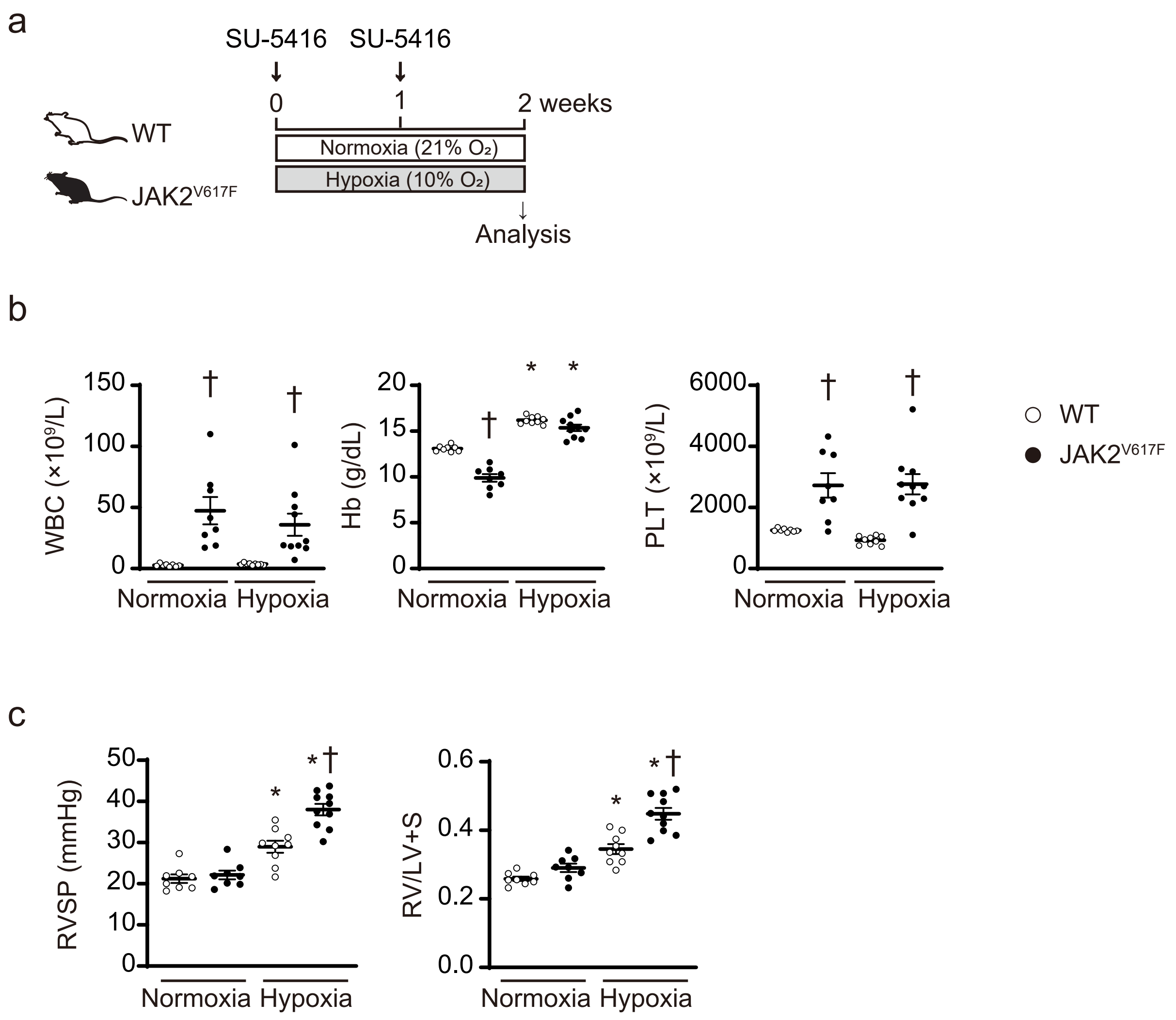


**Supplementary Figure 8. Pulmonary hypertension is accelerated in JAK2$^{V617F}$ mice in a Sugen-hypoxia model.**
**(a)** Experimental design. Single weekly injection of a VEGF inhibitor, SU-5416, at 20 mg/kg followed by 2 weeks of normoxia (21% $O_2$) or hypoxia (10% $O_2$) in WT mice and JAK2$^{V617F}$ mice. **(b)** Peripheral blood cell counts (n = 8, 8, 9, 10, †P = 0.0014 [left], 0.0142 [right] for WBC, n = 8, 8, 9, 10, *P < 0.0001 [left], < 0.0001 [right], †P< 0.0001 for Hb, n = 8, 8, 9, 10, †P = 0.0040 [left], < 0.0001 [right] for PLT). **(c)** Right ventricular systolic pressure (RVSP) and right ventricular hypertrophy determined by the ratio of right ventricle (RV) weight to left ventricle weight plus septum weight (RV/LV+S) (n = 8, 8, 9, 10, *P = 0.0012 [left], < 0.0001 [right], †P < 0.0001 for RVSP, n = 8, 8, 9, 10, *P = 0.0007 [left], < 0.0001 [right], †P < 0.0001 for RV/LV+S). All data are presented as mean ± SEM. *P < 0.05 versus the corresponding normoxia-exposed group and †P < 0.05 versus the corresponding WT mice by the one-way ANOVA with Tukey post-hoc analysis. WBC, white blood cell count; Hb, hemoglobin concentration; PLT, platelet count. WT, wild-type mice; JAK2$^{V617F}$, JAK2V617F-expressing transgenic mice. Source data are provided as a Source Data file.

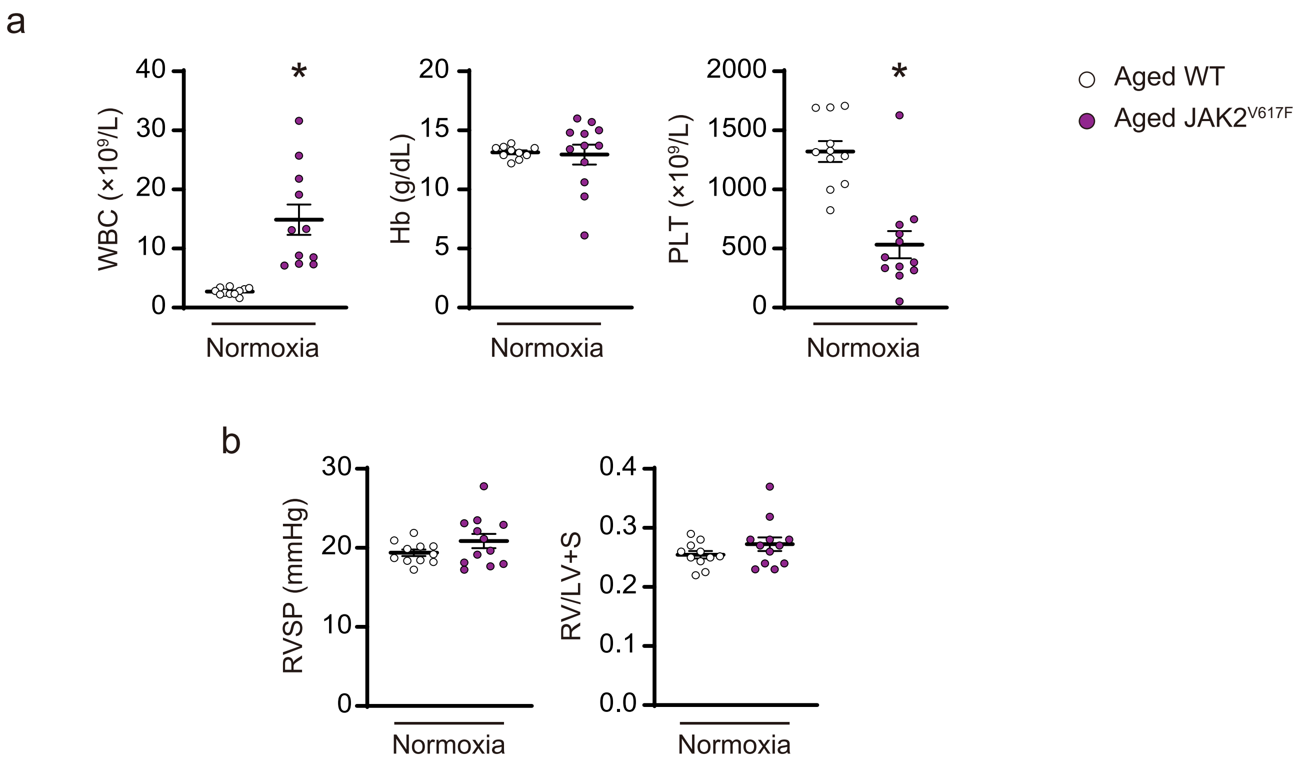


**Supplementary Figure 9. Characterization of aged JAK2$^{V617F}$ mice in the setting of normoxia.**

**(a)** Peripheral blood cell counts in WT and JAK2$^{V617F}$ female mice aged 8- to 9-month-old under normoxia (n = 12 in each group, *P = 0.0001 for WBC, n = 10, 12 for Hb, n = 11, 12, *P < 0.0001 for PLT). **(b)** Right ventricular systolic pressure (RVSP) and right ventricular hypertrophy determined by the ratio of right ventricle (RV) weight to left ventricle weight plus septum weight (RV/LV+S) (n = 11, 12 in each). All data are presented as mean ± SEM. $^{*}$P < 0.05 versus WT mice by the unpaired Student's t-test (two-sided). WBC, white blood cell count; Hb, hemoglobin concentration; PLT, platelet count. WT, wild-type mice; JAK2$^{V617F}$, JAK2V617F-expressing transgenic mice. Source data are provided as a Source Data file.

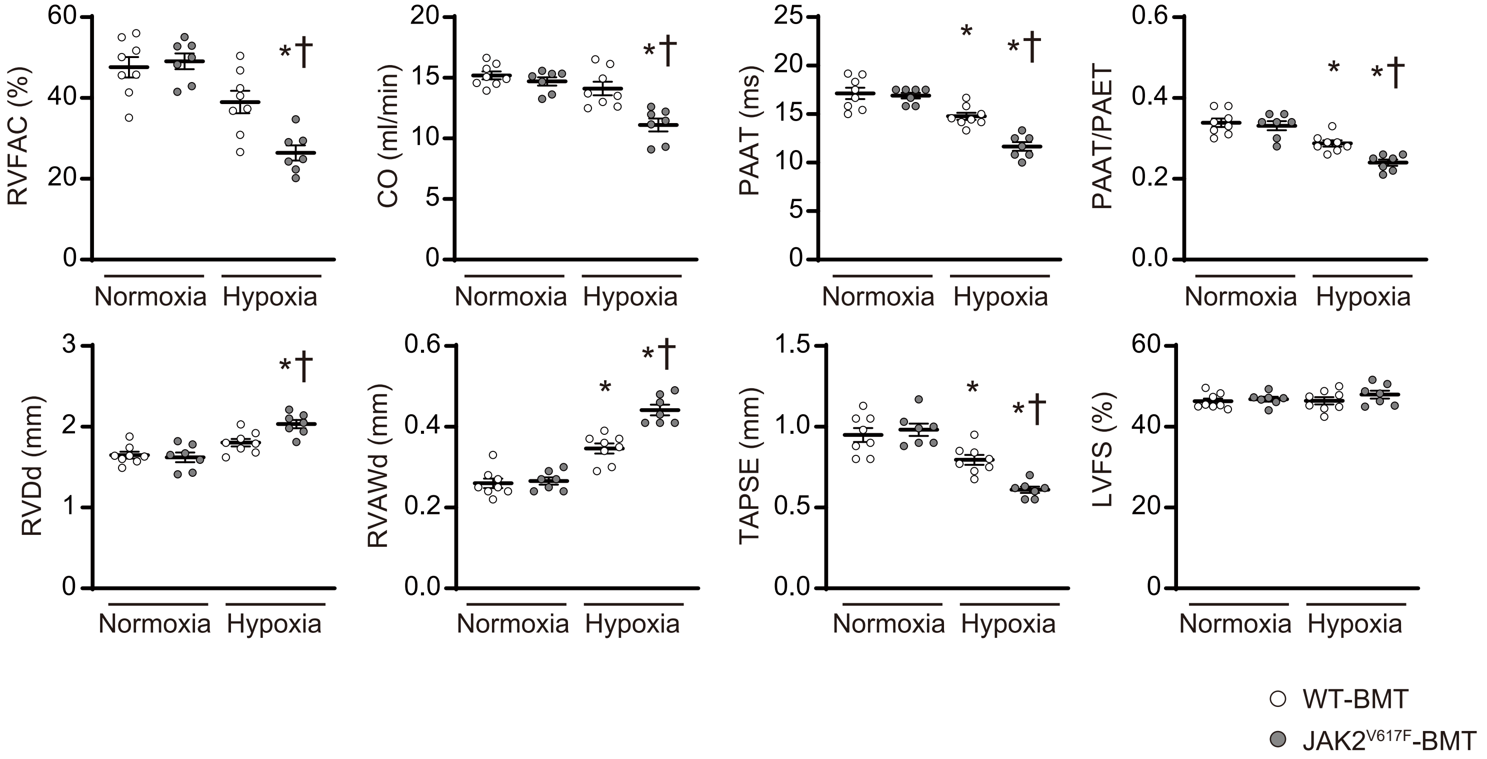


**Supplementary Figure 10. Echocardiographic analyses in JAK2$^{V617F}$-BMT mice after exposure to chronic hypoxia.**

Echocardiography was performed to evaluate pulmonary hemodynamics and cardiac function 3 weeks after normoxia or chronic hypoxia (n = 8, 7, 8, 7, *P < 0.0001, $^{\dagger}$P = 0.0047 for RVFAC, *P < 0.0001, $^{\dagger}$P = 0.0004 for CO, *P = 0.0036 [left], < 0.0001 [right], $^{\dagger}$P = 0.0002 for PAAT, *P = 0.0027 [left], <0.0001 [right], $^{\dagger}$P = 0.0075 for PAAT/PAET, *P < 0.0001, $^{\dagger}$P = 0.0144 for RVDd, *P < 0.0001 [left], < 0.0001 [right], $^{\dagger}$P < 0.0001 for RVAWd, *P = 0.0168 [left], < 0.0001 [right], $^{\dagger}$P = 0.0041 for TAPSE). All data are presented as mean ± SEM. $^{*}$P < 0.05 versus the corresponding normoxia-exposed group and $^{\dagger}$P < 0.05 versus the corresponding WT mice by one-way ANOVA with Tukey post-hoc analysis. RVFAC, right ventricular fractional area change; CO, cardiac output; PAAT, pulmonary artery acceleration time; PAET, pulmonary artery ejection time; RVDd, right ventricular diastolic diameter, RVAWd, right ventricular anterior wall diameter; TAPSE, tricuspid annular plane systolic excursion; LVFS, left ventricular fractional shortening; WT-BMT, recipient WT mice transplanted with WT bone marrow cells; JAK2$^{V617F}$-BMT, recipient WT mice transplanted with JAK2$^{V617F}$ bone marrow cells. Source data are provided as a Source Data file.

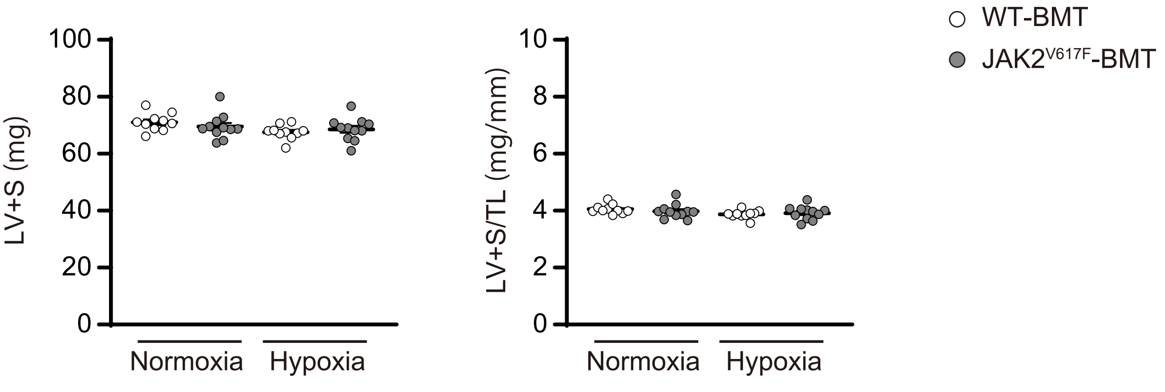


**Supplementary Figure 11. Left ventricular weight in JAK2$^{V617F}$-BMT mice after exposure to chronic hypoxia.**

Left ventricular (LV) weight including septum (S) was measured after exposure to normoxia (21% $O_2$) or chronic hypoxia (10% $O_2$) for 3 weeks (n = 10, 11, 10, 11 in each). LV+S was normalized by tibia length (TL). All data are presented as mean ± SEM. The statistical comparison was performed by the one-way ANOVA. WT-BMT, recipient WT mice transplanted with WT bone marrow cells; JAK2$^{V617F}$-BMT, recipient WT mice transplanted with JAK2$^{V617F}$ bone marrow cells. Source data are provided as a Source Data file.

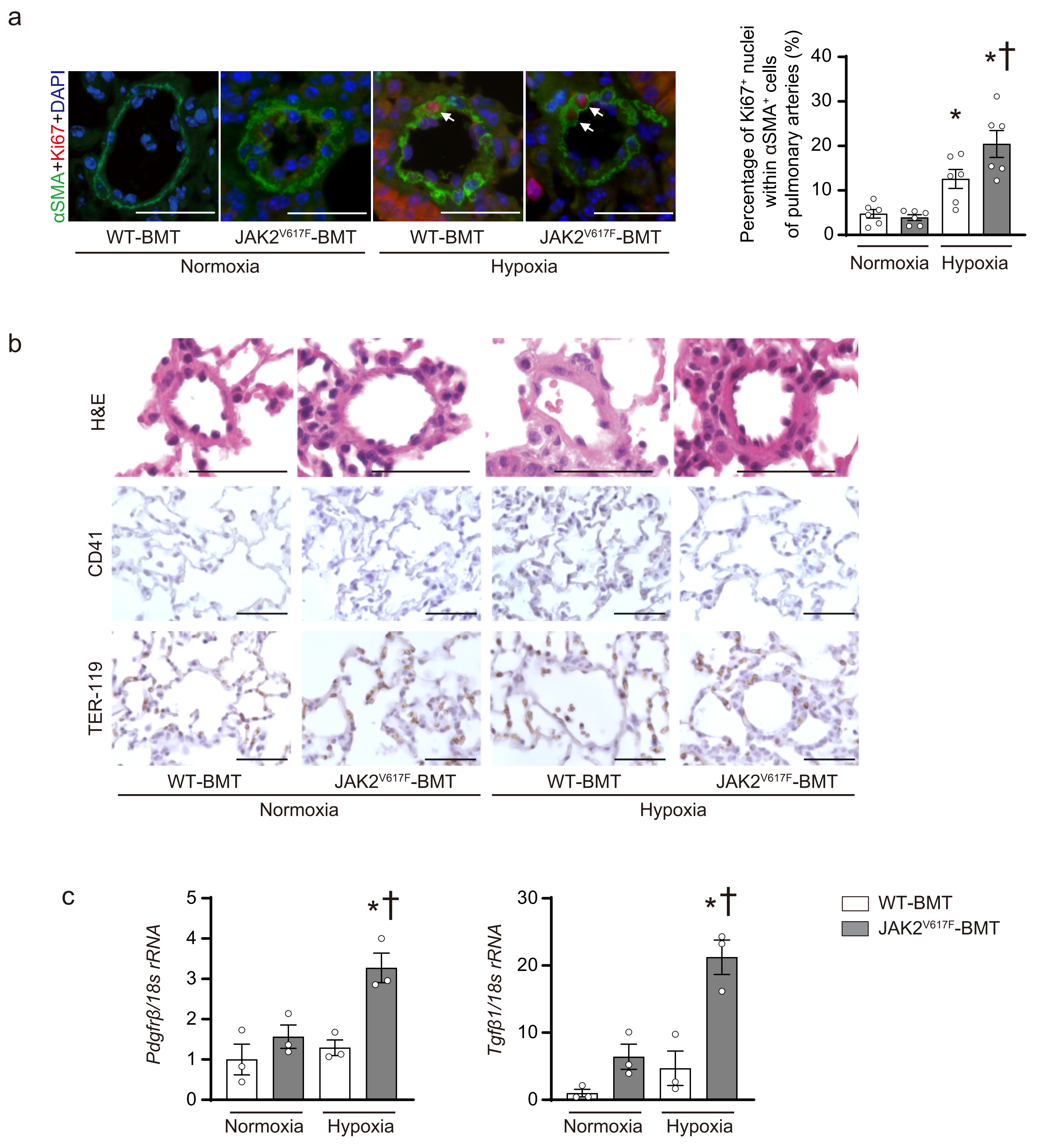


**Supplementary Figure 12. Histological images and mRNA expression of the lungs in JAK2$^{V617F}$-BMT mice after exposure to chronic hypoxia.**

**(a)** Left, triple-labeled immunofluorescent staining (αSMA, green; Ki67, red; DAPI, blue) of the lung sections. Right, quantitative analyses of the percentage of Ki67-positive nuclei within αSMA$^+$ cells of distal pulmonary arteries with a diameter of 50-100 μm (n = 6 in each group, *P = 0.0430 [left], < 0.0001 [right], $^{\dagger}$P = 0.0425). More than 80 αSMA$^+$ cells were counted in each section. White arrows indicate Ki67-positive nuclei within αSMA$^+$ cells. Scale bars, 50 μm. **(b)** Representative images of the lung sections of H&E staining and immunostaining for CD41 and TER-119 from WT-BMT mice and JAK2$^{V617F}$-BMT mice after normoxia and chronic hypoxia. Scale bars, 50 μm. **(c)** mRNA levels of *Pdgfrb* and *Tgfb1* in the lungs. The *18s rRNA* was used for the normalization. Data are presented as mean ± SEM. The average value for the normoxia-WT-BMT mice was set to 1 (n = 3 in each group, *P = 0.0214, $^{\dagger}$P = 0.0095 for *Pdgfrb*, *P = 0.0041, $^{\dagger}$P = 0.0021 for *Tgfb1*). All data are presented as mean ± SEM. $^{*}$P < 0.05 versus the corresponding normoxia-exposed group and $^{\dagger}$P < 0.05 versus the corresponding WT-BMT mice by one-way ANOVA with Tukey post-hoc analysis. WT-BMT, recipient WT mice transplanted with WT bone marrow cells; JAK2$^{V617F}$-BMT, recipient WT mice transplanted with JAK2$^{V617F}$ bone marrow cells. Source data are provided as a Source Data file.

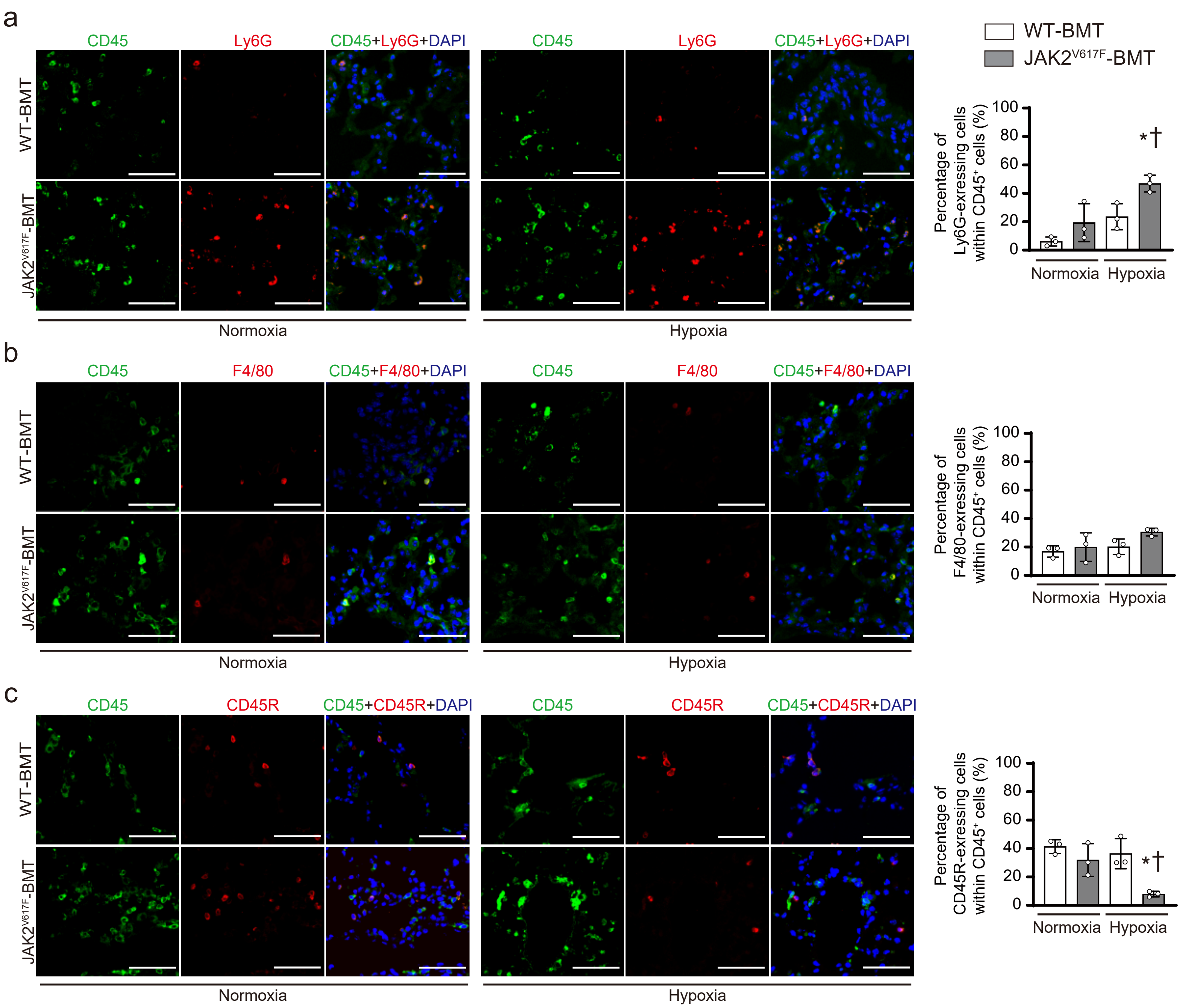


**Supplementary Figure 13. Characterization of the infiltrated leukocytes in the pulmonary arterial regions in JAK2$^{V617F}$-BMT mice after exposure to chronic hypoxia.**

**(a)** Left, triple-labeled immunofluorescent staining (CD45, green; Ly6G, red; DAPI, blue) of the lung sections from WT mice and JAK2$^{V617F}$ mice after normoxia or chronic hypoxia. Right, quantitative analyses of the Ly6G-expressing cells within CD45$^{+}$ cells (n = 3 in each group, *P = 0.0205, $^{†}$P = 0.0459). **(b)** Left, triple-labeled immunofluorescent staining (CD45, green; F4/80, red; DAPI, blue). Right, quantitative analyses of the F4/80-expressing cells within CD45$^{+}$ cells (n = 3 in each group). **(c)** Left, triple-labeled immunofluorescent staining (CD45, green; CD45R, red; DAPI, blue). Right, quantitative analyses of the CD45R-expressing cells within CD45$^{+}$ cells (n = 3 in each group, *P = 0.0309, $^{†}$P = 0.0125). At least 100 CD45$^{+}$ cells were counted in each. All data are presented as mean ± SEM. $^{*}$P < 0.05 versus the corresponding normoxia-exposed group and $^{†}$P < 0.05 versus the corresponding WT-BMT mice by one-way ANOVA with Tukey post-hoc analysis. Scale bars, 50 μm. WT-BMT, recipient WT mice transplanted with WT bone marrow cells; JAK2$^{V617F}$-BMT, recipient WT mice transplanted with JAK2$^{V617F}$ bone marrow cells. Source data are provided as a Source Data file.

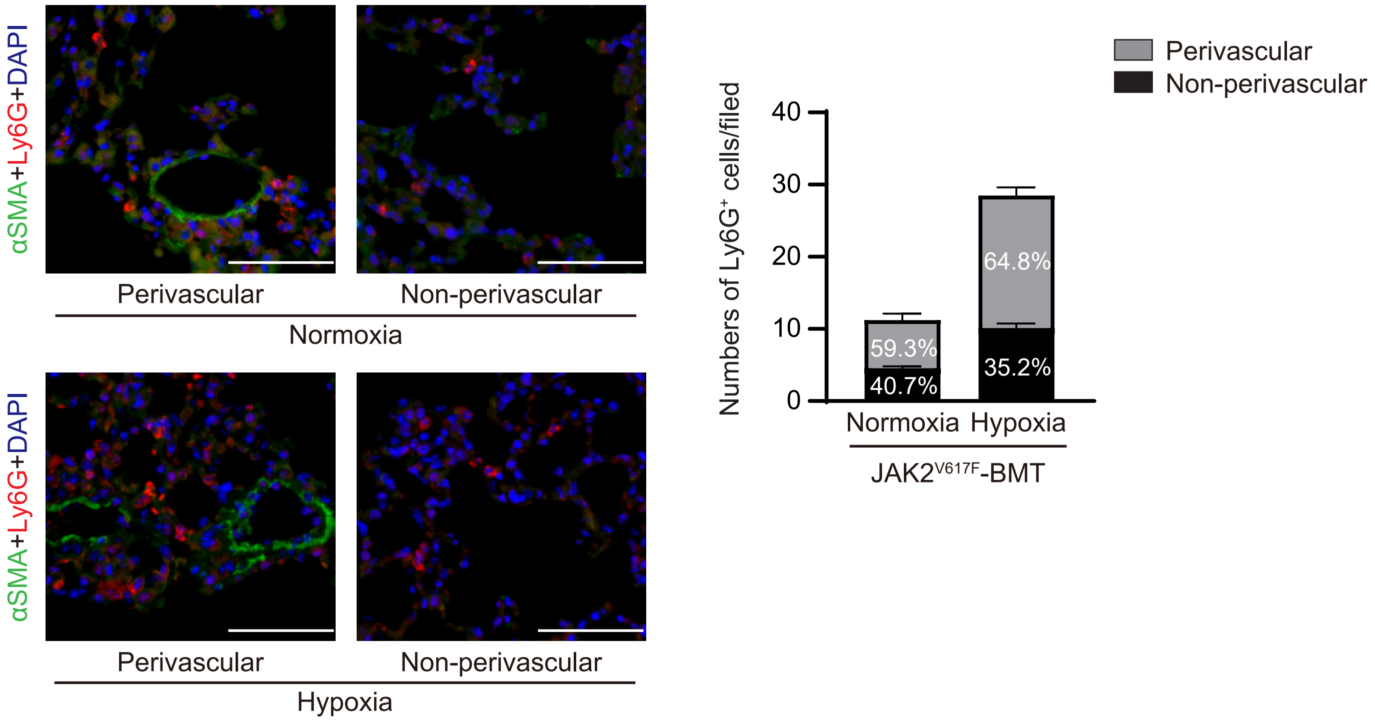


**Supplementary Figure 14. Chronic hypoxia increased Ly6G⁺ neutrophils in perivascular regions as well as non-perivascular regions of the lungs in JAK2$^{V617F}$-BMT mice.**

Left. triple-labeled immunofluorescent staining (αSMA, green; Ly6G, red; DAPI, blue) of the lung sections in JAK2$^{V617F}$-BMT mice. Perivascular regions were determined as the area within 100 μm from distal pulmonary arteries with diameters of 50 μm. Scale bars, 50 μm. Right, quantitative analyses of the numbers of Ly6G⁺ cells in perivascular regions as well as non-perivascular regions (n = 3). More than 10 fields were analyzed in each group. One field was defined as 200 μm x 200 μm. The percentages of the Ly6G⁺ cells in perivascular regions and non-perivascular regions in each group are shown. JAK2$^{V617F}$-BMT, recipient WT mice transplanted with JAK2$^{V617F}$ bone marrow cells. All data are presented as mean ± SEM. Source data are provided as a Source Data file.

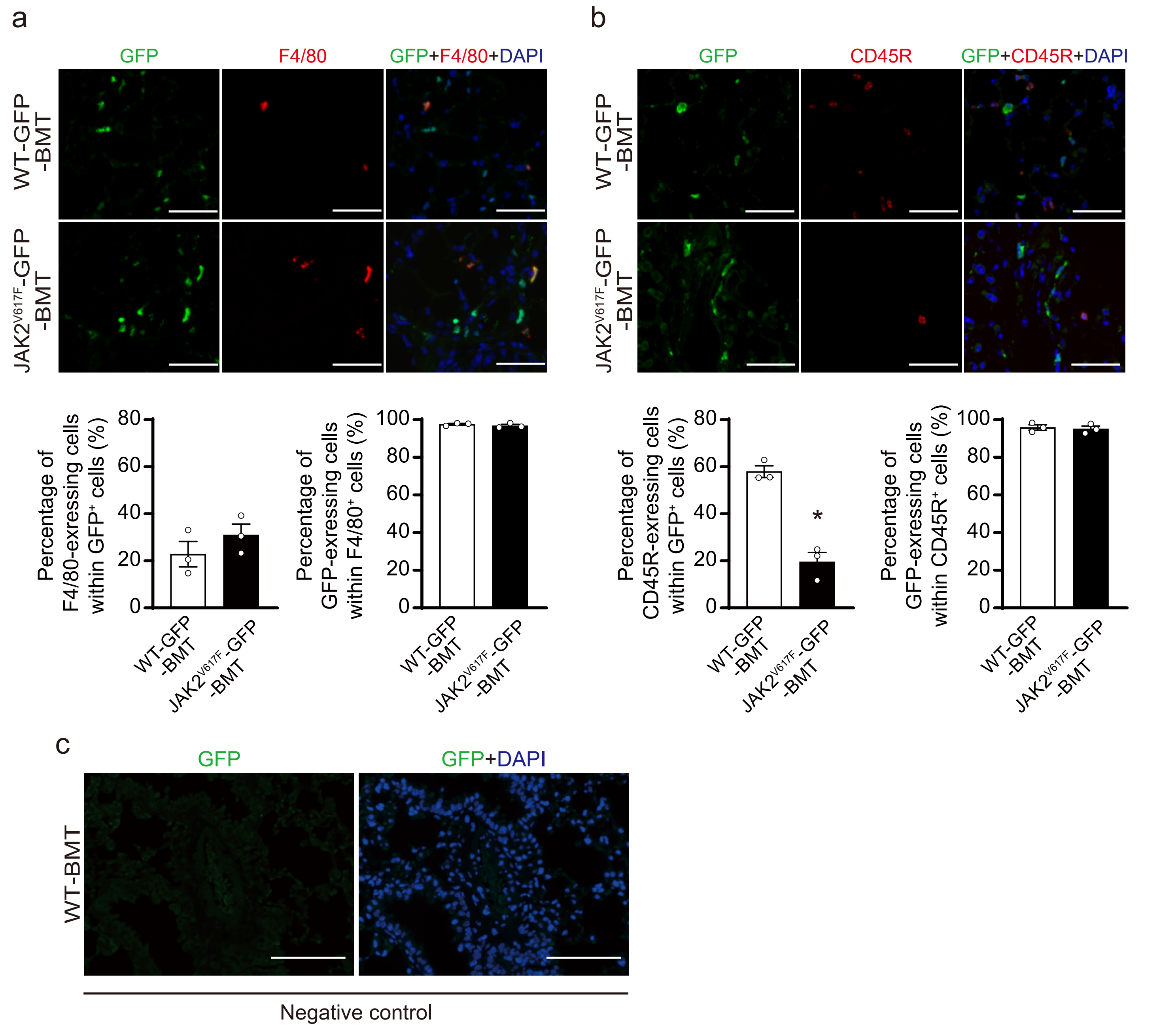


**Supplementary Figure 15. Characterization of bone marrow-derived JAK2V617F hematopoietic cells in the lungs by the use of GFP-transgene.**

**(a)** Lethally irradiated WT mice were transplanted with bone marrow (BM) cells from control WT/CAG-EGFP (WT-GFP) or JAK2V617F/CAG-EGFP (JAK2$^{V617F}$-GFP) double transgenic mice. Five weeks after BM transplantation (BMT), recipients were subjected to chronic hypoxia for 3 weeks, and then the lungs were fixed and stained with the indicated antibodies. Representative immunofluorescence images of lung sections stained with anti-GFP (green) and anti-F4/80 (red) antibodies and DAPI (blue) in WT-GFP-BMT or JAK2$^{V617F}$-GFP-BMT mice are shown in top panels. Scale bars, 50 µm. Quantitative analyses of F4/80-expressing cells within GFP$^+$ cells and GFP-expressing cells within F4/80$^+$ cells are shown in the graphs (n = 3 in each group). **(b)** Representative immunofluorescence images of lung sections stained with anti-GFP (green) and anti-CD45R (red) antibodies and DAPI (blue) in WT-GFP-BMT or JAK2$^{V617F}$-GFP-BMT mice. Scale bars, 50 µm. Quantitative analyses of CD45R-expressing cells within GFP$^+$ cells and GFP-expressing cells within CD45R$^+$ cells are shown in the graphs (n = 3 in each group). More than 100 cells were counted in each. *P = 0.0012 versus WT-GFP-BMT mice by the unpaired t-test (two-sided). All data are presented as mean ± SEM. **(c)** The lung sections from WT recipient mice transplanted with WT BM cells without GFP as a negative control. The sections were stained with an anti-GFP (green) antibody and DAPI (blue). Representative images of three independent experiments are shown. Scale bars, 100 µm. WT-GFP-BMT, recipient WT mice transplanted with WT-GFP BM cells; JAK2$^{V617F}$-GFP-BMT, recipient WT mice transplanted with JAK2$^{V617F}$-GFP BM cells. Source data are provided as a Source Data file.

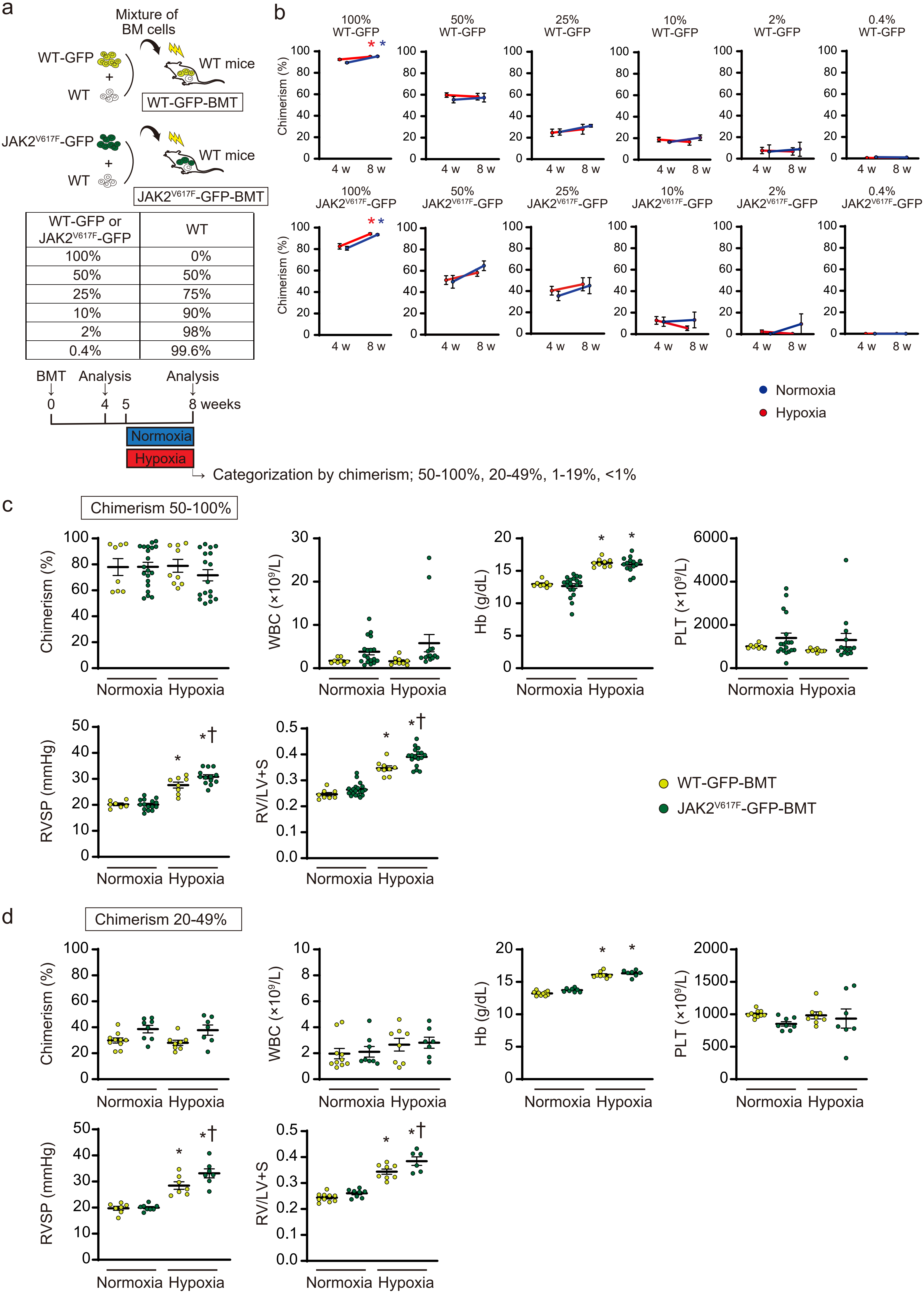

a
Mixture of BM cells
WT-GFP
+
WT
WT mice
WT-GFP-BMT
JAK2V617F-GFP
+
WT
WT mice
JAK2V617F-GFP-BMT
WT-GFP or JAK2V617F-GFP | WT
100% | 0%
50% | 50%
25% | 75%
10% | 90%
2% | 98%
0.4% | 99.6%
BMT
Analysis
Analysis
0
4
5
8 weeks
Normoxia
Hypoxia
Categorization by chimerism; 50-100%, 20-49%, 1-19%, <1%
b
100% WT-GFP
50% WT-GFP
25% WT-GFP
10% WT-GFP
2% WT-GFP
0.4% WT-GFP
100% JAK2V617F-GFP
50% JAK2V617F-GFP
25% JAK2V617F-GFP
10% JAK2V617F-GFP
2% JAK2V617F-GFP
0.4% JAK2V617F-GFP
Chimerism (%)
4 w
8 w
Normoxia
Hypoxia
c
Chimerism 50-100%
Chimerism (%)
WBC (×10⁹/L)
Hb (g/dL)
PLT (×10⁹/L)
RVSP (mmHg)
RV/LV+S
Normoxia
Hypoxia
WT-GFP-BMT
JAK2V617F-GFP-BMT
d
Chimerism 20-49%
Chimerism (%)
WBC (×10⁹/L)
Hb (g/dL)
PLT (×10⁹/L)
RVSP (mmHg)
RV/LV+S
Normoxia
Hypoxia

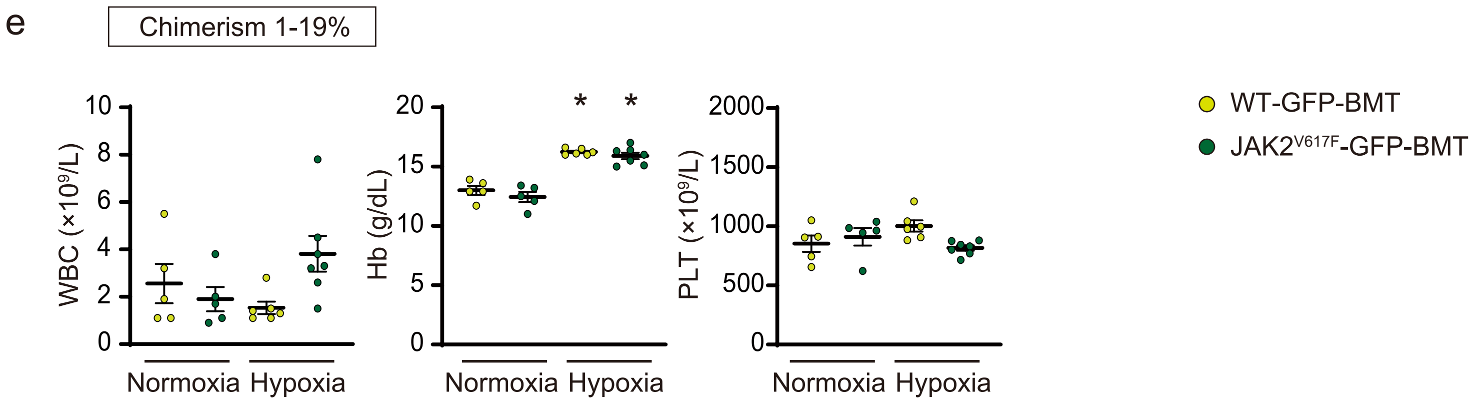


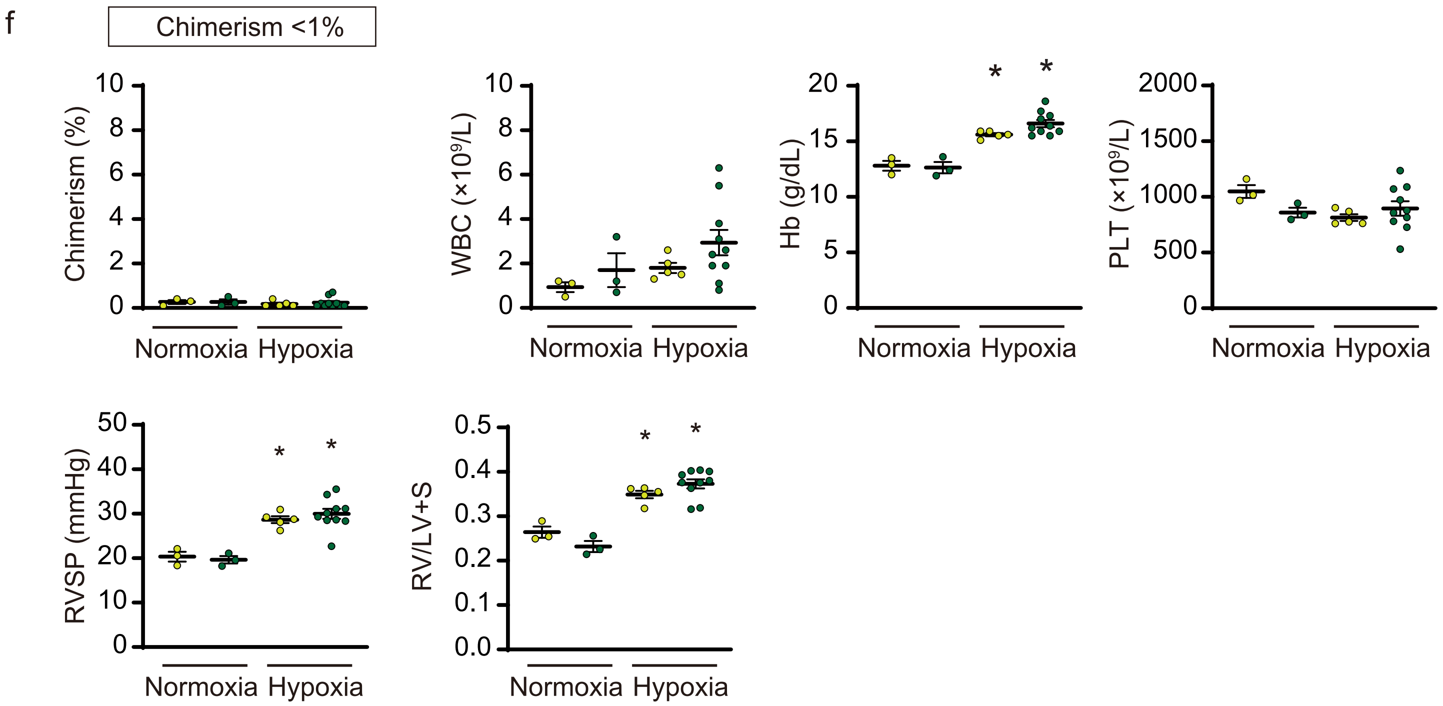


**Supplementary Figure 16. A competitive transplantation model using $JAK2^{V617F}$-GFP bone marrow cells.**

**(a)** Schematic depiction of the competitive bone marrow transplantation (BMT). The different ratio of WT-GFP or $JAK2^{V617F}$-GFP and WT without GFP competitor was transplanted into the lethally irradiated recipient WT mice. The BMT mice at 8 weeks were categorized according to the chimerism; 50-100%, 20-49%, 1-19%, <1%. **(b)** The donor chimerism in the blood after BMT. The percentage of GFP$^+$ cells within CD45$^+$ cells was determined by flow cytometry at 4 and 8 weeks after BMT after normoxia or chronic hypoxia exposure (n= 9, 9, *P = 0.0001 [normoxia], 0.0211 [hypoxia] for 100% WT-GFP, n = 5, 5 for 50% WT-GFP, n = 5, 5 for 25% WT-GFP, n = 5, 5 for 10% WT-GFP; n = 5, 6 for 2% WT-GFP, n = 5, 5 for 0.4% WT-GFP, n = 11, 9, *P < 0.0001 [normoxia], 0.0025 [hypoxia] for 100% $JAK2^{V617F}$-GFP, n = 10, 6 for 50% $JAK2^{V617F}$-GFP, n = 8, 14 for 25% $JAK2^{V617F}$-GFP, n = 5, 4 for 10% $JAK2^{V617F}$-GFP, n = 5, 8 for 2% $JAK2^{V617F}$-GFP, n = 5, 7 for 0.4% $JAK2^{V617F}$-GFP). ˙P < 0.05 versus the corresponding 4-week group by the two-sided paired Student's t-test (blue, normoxia group; red, hypoxia group). **(c, d)** The recipients with donor chimerism of 50-100% (**c**, n = 8, 20, 9, 17) and 20-49% (**d**, n = 10, 8, 8, 7) at 8 weeks after BMT were enrolled for statistical comparison. Peripheral blood cell counts, RVSP, and RV/LV+S are shown (**c**, n = 8, 18, 9, 14 for WBC, n = 8, 19, 9, 14, *P < 0.0001 [left], < 0.0001 [right] for Hb, n = 8, 19, 9, 14 for PLT, n = 7, 15, 8, 13, *P < 0.0001 [left], < 0.0001 [right], †P = 0.0339 for RVSP, n = 8, 20, 9, 17, *P < 0.0001 [left], < 0.0001 [right], †P < 0.0021 for RV/LV+S; **d**, n = 10, 8, 8, 7 for WBC, n = 10, 8, 8, 7, *P < 0.0001 [left], < 0.0001 [right] for Hb, n = 10, 8, 8, 7 for PLT, n = 7, 8, 7, 7, *P = 0.0001 [left], < 0.0001 [right], †P = 0.0445 for RVSP, n = 10, 8, 8, 6, *P < 0.0001 [left], < 0.0001 [right], †P = 0.0291 for RV/LV+S). **(e)** Peripheral blood cell counts in the BMT mice with donor chimerism of 1-19% at 8 weeks after BMT (n = 5, 5, 6, 7 for WBC, n = 5, 5, 6, 7, *P < 0.0001 [left], < 0.0001 [right] for Hb, n = 5, 5, 6, 7 for PLT). The data of chimerism, RVSP, and RV/LV+S are shown in main Fig. 4. **(f)** The donor chimerism, peripheral blood cell counts, RVSP and RV/LV+S in the BMT mice with donor chimerism with <1% (n = 3, 3, 5, 10 for chimerism, n = 3, 3, 5, 10 for WBC, n = 3, 3, 5, 10, *P = 0.0014 [left], < 0.0001 [right] for Hb, n = 3, 3, 5, 10 for PLT, n = 3, 3, 5, 10, *P = 0.0043 [left], 0.0002 [right] for RVSP, n = 3, 3, 5, 10, *P = 0.0027 [left], < 0.0001 [right] for RV/LV+S). All data are presented as mean ± SEM. ˙P < 0.05 versus the corresponding normoxia-exposed group and †P < 0.05 versus the corresponding WT-GFP-BMT mice by the one-way ANOVA with Tukey post-hoc analysis. WBC, white blood cell count; Hb, hemoglobin concentration; PLT, platelet count; WT-GFP-BMT, recipient WT mice transplanted with WT-GFP BM cells; $JAK2^{V617F}$-GFP-BMT, recipient WT mice transplanted with $JAK2^{V617F}$-GFP BM cells. Source data are provided as a Source Data file.

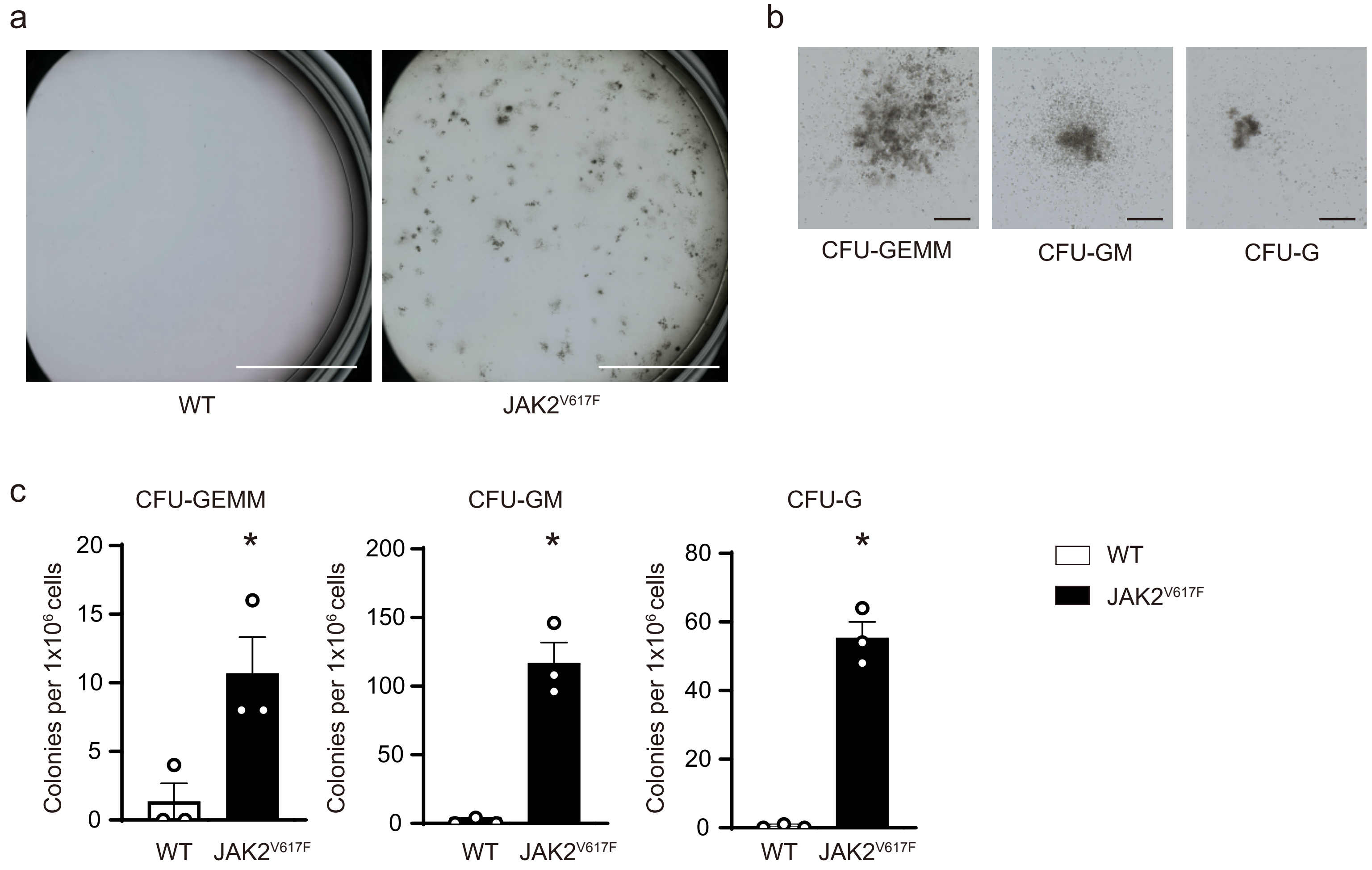


**Supplementary Figure 17. Colony-forming assay to estimate the presence of hematopoietic progenitor cells in the JAK2$^{V617F}$ lungs for the myeloid lineage.**

CD117 (c-kit)$^{+}$ cells were sorted from the mouse lung tissue in WT and JAK2$^{V617F}$ mice using a magnetic bead method. On a 35-mm plate, 5x10$^{5}$ were grown in Methocult GF M3434. After 7 days, the colonies derived from colony-forming unit (CFU)-granulocyte, -erythroid, -macrophage, -megakaryocyte (CFU-GEMM), CFU-granulocyte, -monocyte (CFU-GM), CFU-granulocyte (CFU-G) were counted according to the morphology. **(a, b)** Representative images of the plates and colonies. Scale bars, 10 mm (a) and 300 µm (b). **(c)** Quantification of numbers of the colonies (n = 3 in each grooup, *P = 0.0352 for CFU-GEMM, *P = 0.0016 for CFU-GM, *P = 0.0003 for CFU-G). All data are presented as mean ± SEM. $^{*}$P < 0.05 versus WT by the unpaired t-test (two-sided). WT, wild-type mice; JAK2$^{V617F}$, JAK2V617F-expressing transgenic mice. Source data are provided as a Source Data file.

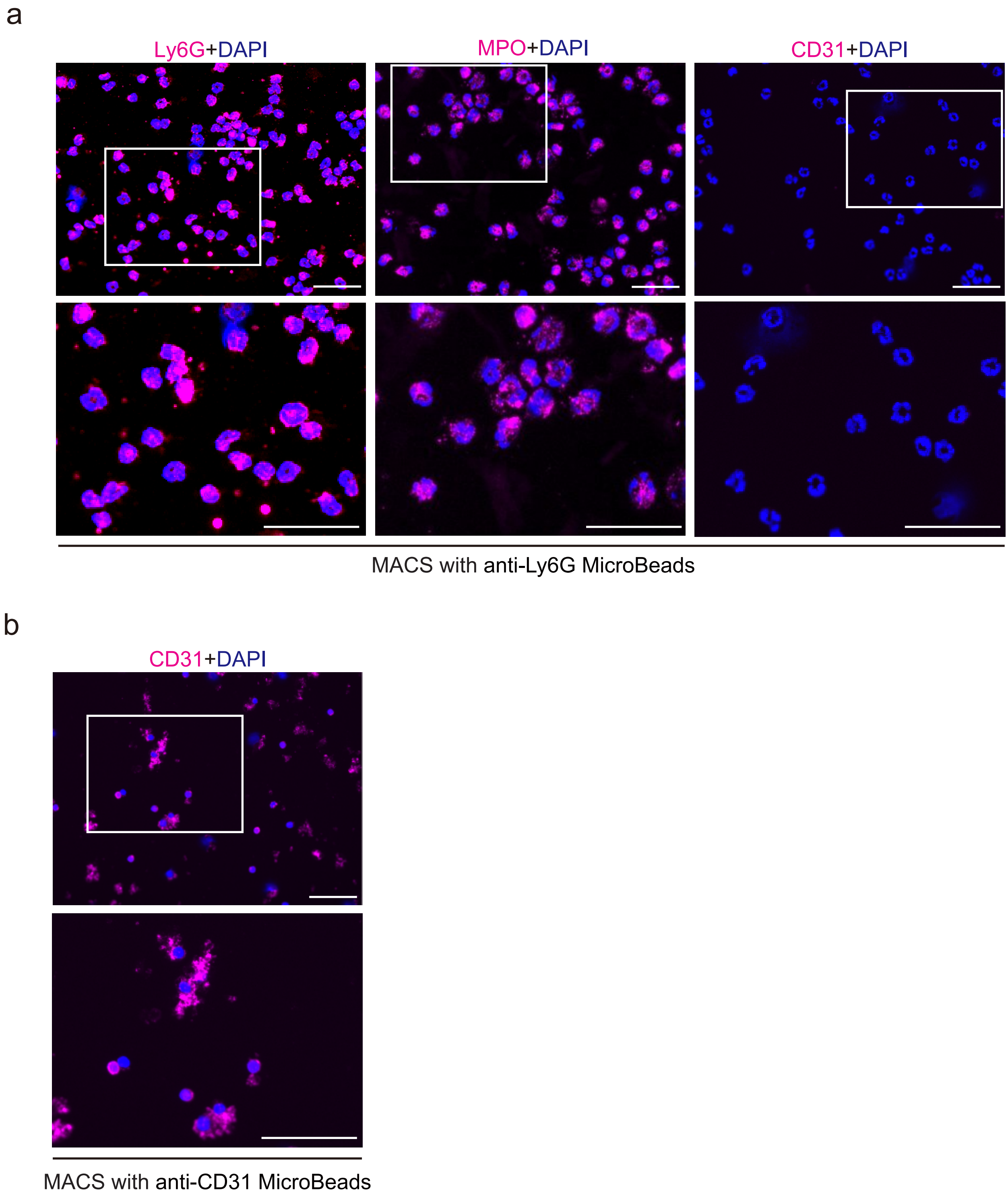


**Supplementary Figure 18. Immunofluorescence of sorted cells from the mouse lung.**

The cell suspensions from the lungs in wild-type mice were subjected to MACS with anti-Ly6G MicroBeads (a) or anti-CD31 MicroBeads (b). The sorted cells were fixed and stained with indicated antibodies (magenta) with DAPI (blue). Images in boxed areas at higher magnification are shown in bottom panels. Scale bars, 50 um. Nearly 100% of the MACS-isolated Ly6G$^{+}$ cells were stained with anti-Ly6G and anti-myeloperoxidase (MPO) antibodies, a specific marker for neutrophils, while these cells were not stained with an anti-CD31 antibody, a specific marker for endothelial cells. Representative images of three independent experiments are shown. MACS, Magnetic-activated cell sorting.

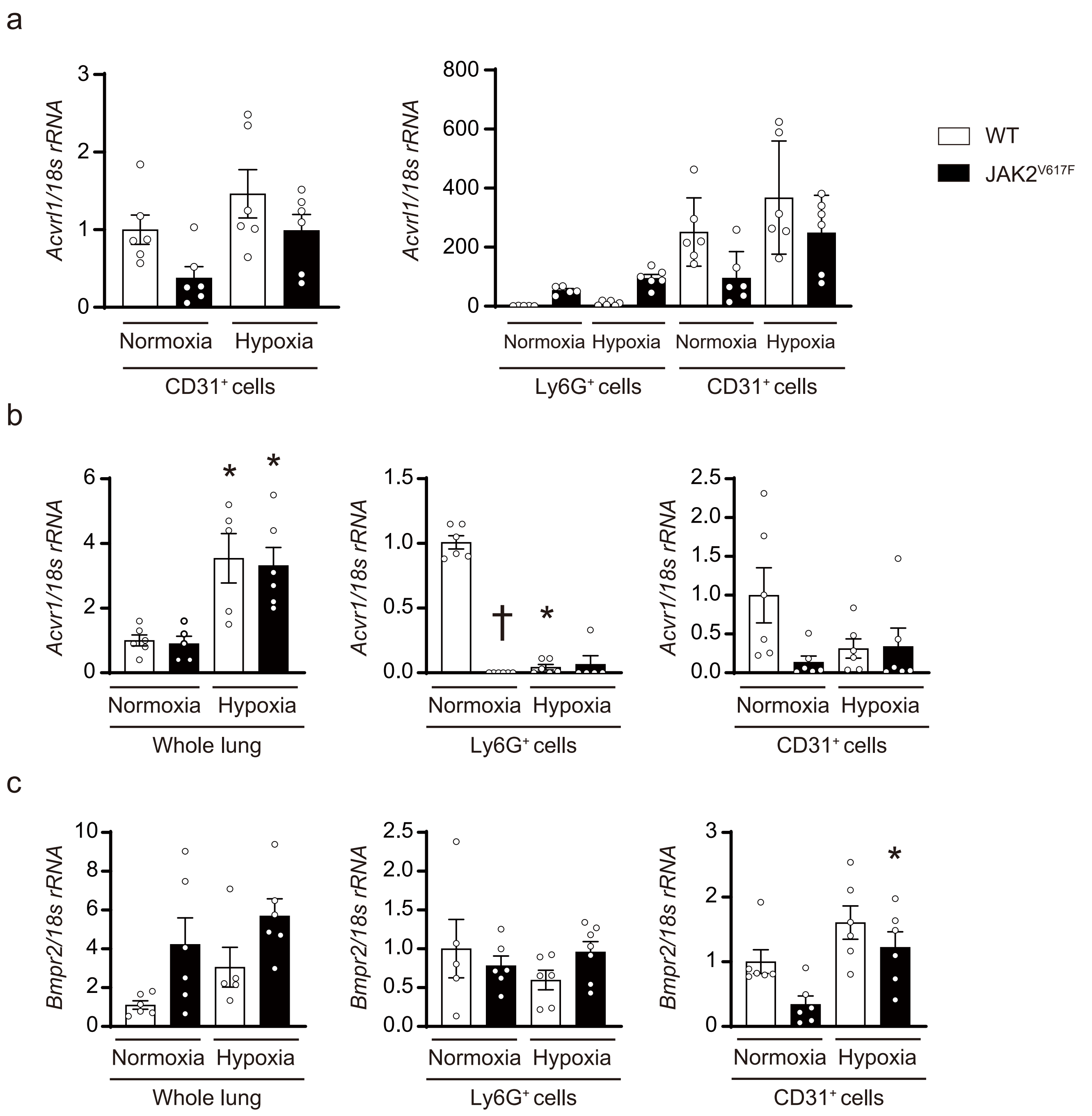


**Supplementary Figure 19. *Acvrl1*, *Acvr1,* and *Bmpr2* mRNA expressions in the lung homogenates or sorted cells from the lungs after exposure to chronic hypoxia.**

**(a)** Left, mRNA expression of *Acvrl1* in the sorted cells by MACS with anti-CD31 MicroBeads from the lungs of WT mice and JAK2$^{V617F}$ mice after normoxia or hypoxia (n = 6 in each group). Right, The comparison of *Acvrl1* mRNA expression levels between Ly6G$^+$ and CD31$^+$ cells from the lungs (n = 5, 5, 6, 6, 6, 6, 6, 6). The data of Ly6G$^+$ cells are from main Figure 6. **(b)** mRNA expression of *Acvr1* in the whole lung extracts (left graph, n = 6, 5, 5, 6, *P = 0.0079 [left], 0.0116 [right]), Ly6G$^+$ cells from the lungs (middle graph, n = 6, 6, 6, 5, $^†$P < 0.0001), CD31$^+$ cells from the lungs (right graph, n = 6 in each group). **(c)** mRNA levels of *Bmpr2* in the whole lung extracts (left graph, n = 6, 6, 5, 6), Ly6G$^+$ cells from the lungs (middle graph, n = 5, 5, 6, 7), CD31$^+$ cells from the lungs (right graph, n = 6 in each group, *P = 0.0338). The *18s rRNA* was used for the normalization. Data are presented as mean ± SEM. The average value for the normoxia-WT mice was set to 1. *P < 0.05 versus the corresponding normoxia-exposed group and $^†$P < 0.05 versus the corresponding WT mice by the one-way ANOVA with Tukey post-hoc analysis. WT, wild-type mice; JAK2$^{V617F}$, JAK2V617F-expressing transgenic mice. Source data are provided as a Source Data file.

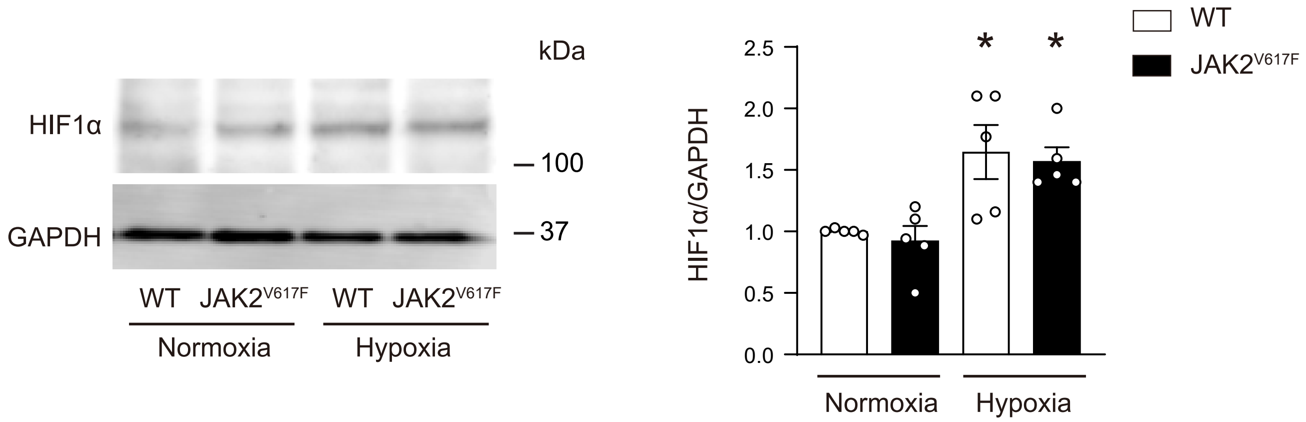


**Supplementary Figure 20. HIF1α expression in the lungs of JAK2$^{V617F}$ mice in response to exposure to chronic hypoxia.** Immunoblotting of HIF1α in WT mice and JAK2$^{V617F}$ mice after normoxia and chronic hypoxia. Left panels show representative blots. Densitometric analysis is shown in the right graph (n = 5 in each group, *P = 0.0200 [left], 0.0200 [right]). GAPDH was used as the loading control. The average value for the normoxia-WT mice was set to 1. Data are presented as mean ± SEM. *P < 0.05 versus the corresponding normoxia-exposed group by the one-way ANOVA with Tukey post-hoc analysis. WT, wild-type mice; JAK2$^{V617F}$, JAK2V617F-expressing transgenic mice. Source data are provided as a Source Data file.

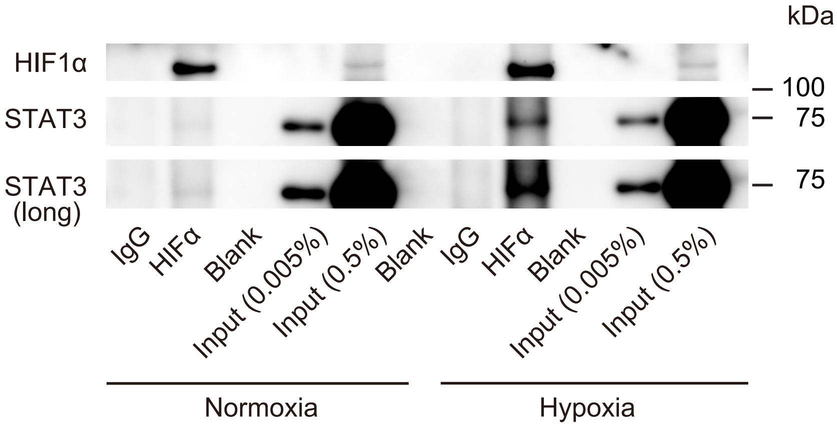


**Supplementary Figure 21. Binding of HIF1α and STAT3 in the lungs.**
Co-immunoprecipitation of STAT3 and HIF1α in the lung tissue of JAK2$^{V617F}$ mice after exposure to normoxia and chronic hypoxia for 2 weeks. The lung homogenates were immunoprecipitated with Rabbit IgG or an anti-HIF1α antibody and subjected to immunoblotting with anti-HIF1α and anti-STAT3 antibodies. The two blots from the bottom were originated from the same membrane, and the longer exposure time was used in the bottom blot for clarity (long). Representative images of two independent experiments are shown. Source data are provided as a Source Data file.

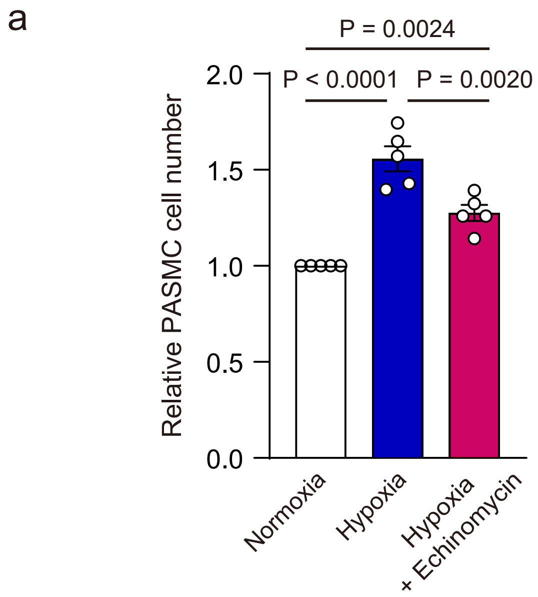


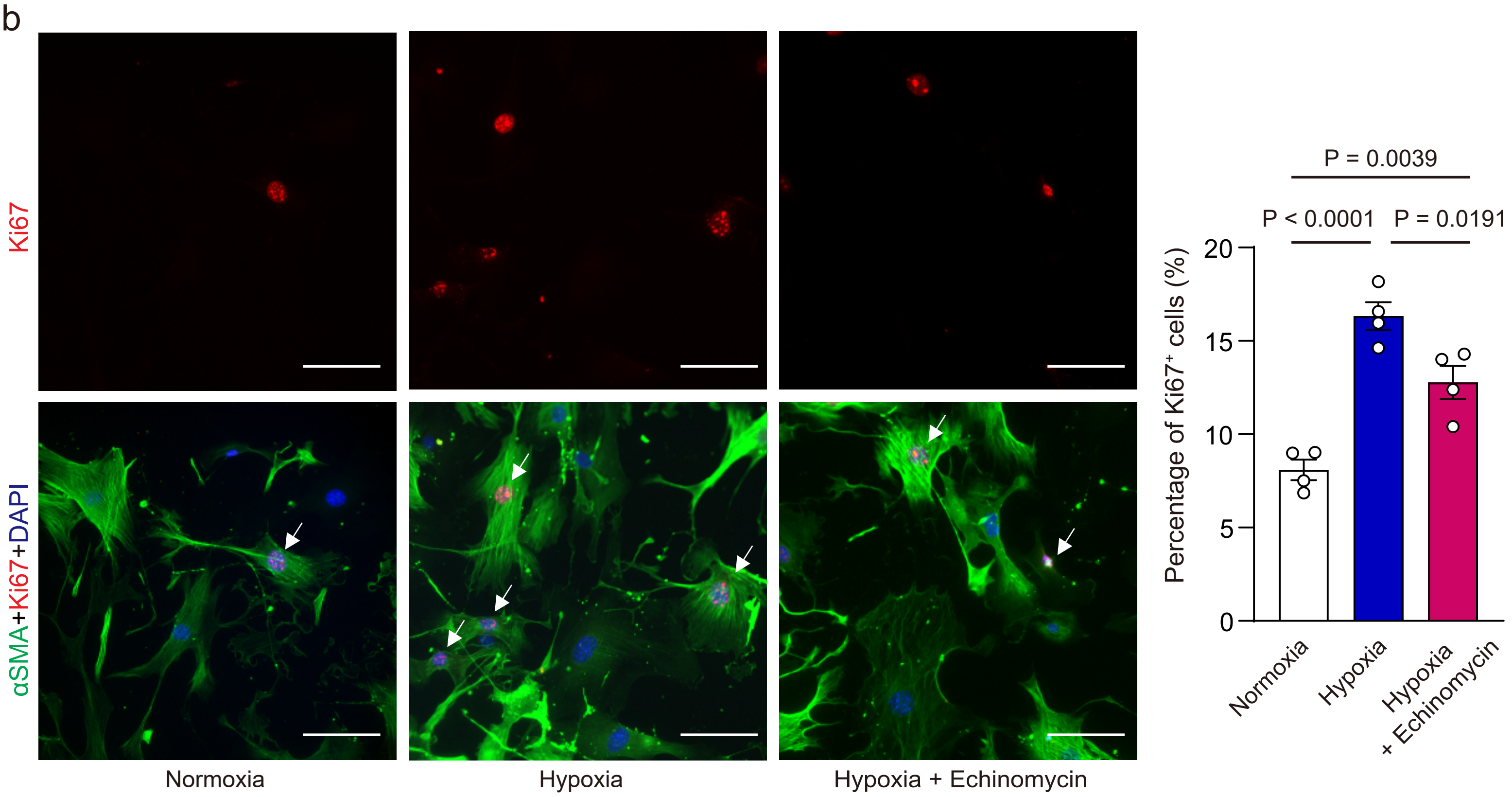


**Supplementary Figure 22. Inhibition of HIF1α in JAK2V617F-expressing neutrophils reduced the mouse pulmonary artery smooth muscle cell (PASMC) proliferation.**

**(a)** The neutrophils were collected from peripheral blood of $JAK2^{V617F}$ mice by MACS with $Ly6G^{+}$ MicroBeads. Cells were starved and then incubated in a hypoxia incubator chamber (10% $O_2$) for 3 h. The neutrophils were pretreated with Echinomycin (1 nM), an anti-HIF1α inhibitor, prior to hypoxia for 1 h, and then the medium was freshly changed just before hypoxia stimulation. Control neutrophils were cultured in normoxic conditions for 3 h. The conditioned medium was collected and centrifuged to remove the cell debris. PASMC were incubated with the neutrophil-derived conditioned medium for 48 h. Cell numbers were determined by cell proliferation assay and expressed as a relative ratio over the group of PASMC incubated with conditioned medium from normoxic conditions from 5 independent experiments. **(b)** Left, representative immunofluorescent images of Ki67 staining and triple staining (αSMA, green; Ki67, red; DAPI, blue) of PASMC 48 h after neutrophil-derived conditioned medium stimulation. White arrows indicate Ki67-positive nuclei. Right, quantitative analyses of the Ki67-positive cells. More than 100 cells were counted (n = 4 independent experiments). Scale bars, 100 μm. Data are presented as mean ± SEM. Statistical significance was determined by the one-way ANOVA with Tukey post-hoc analysis. Source data are provided as a Source Data file.

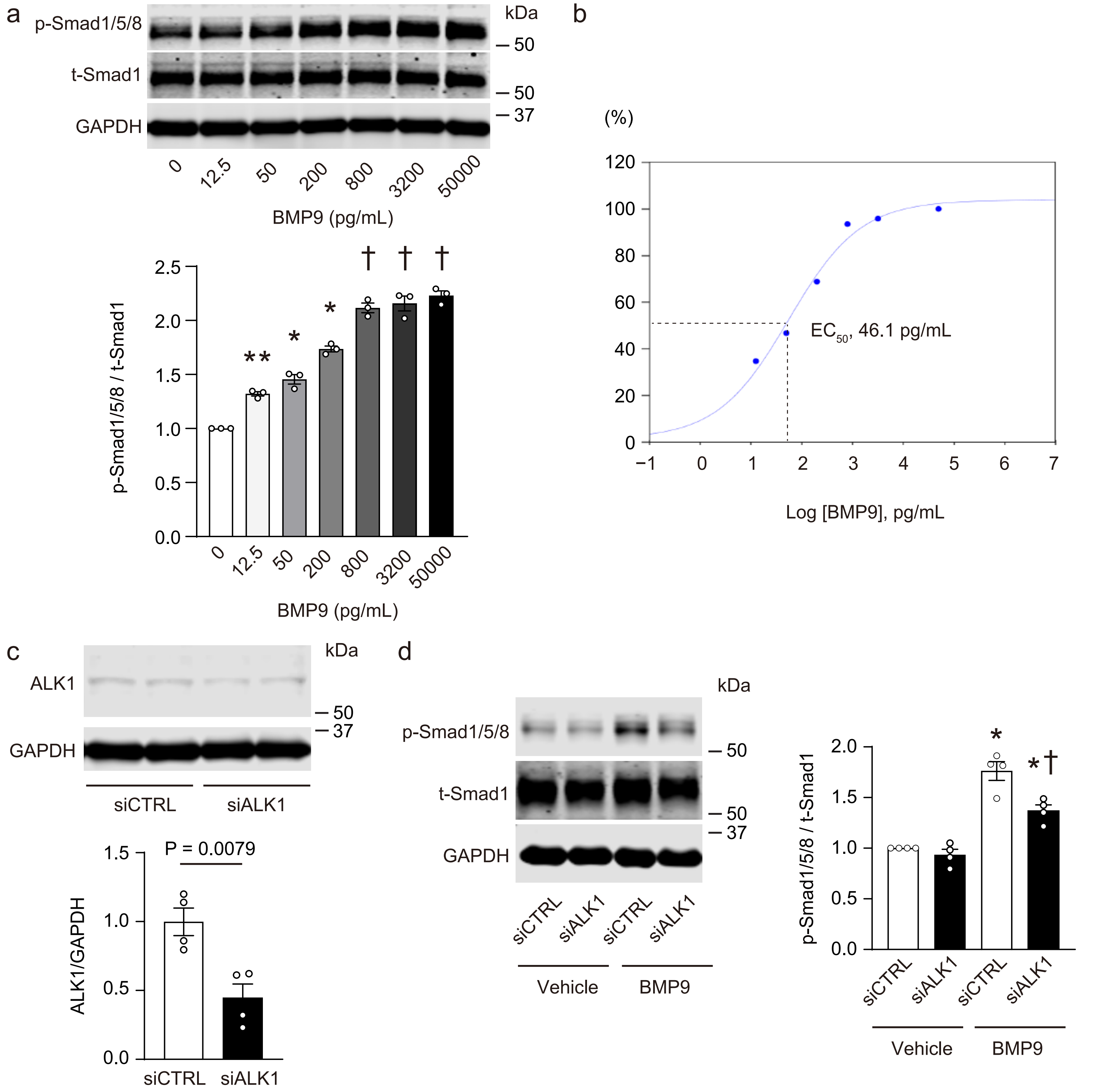


**Supplementary Figure 23. Smad1/5/8 phosphorylation in response to BMP9 stimulation in HCT116 cells to express an active ALK1 receptor.**

**(a)** Immnoblots of Smad1/5/8 phosphorylation in HCT116 cells. HCT116 cells were incubated with BMP9, a high-affinity ALK1 ligand, at the indicated concentration for 3 h, and then the cell lysates were subjected to immunoblotting. p-Smad1/5/8 and t-Smad1 indicate phosphorylated Smad1/5/8 and total Smad1, respectively. Data are presented as mean ± SEM (n = 3 independent experiments). *P < 0.05 versus all other groups, **P < 0.05 versus all other groups except 50 pg/mL of BMP9 and †P < 0.05 versus all other groups except 800, 3200, 5000 pg/mL of BMP9 by the one-way ANOVA with Tukey post-hoc analysis. P = 0.0011 (0 vs. 12.5), < 0.0001 (0 vs. 50), < 0.0001 (0 vs. 200), < 0.0001 (0 vs. 800), < 0.0001 (0 vs. 3200), < 0.0001 (0 vs. 50000), 0.2984 (12.5 vs. 50), < 0.0001 (12.5 vs. 200), < 0.0001 (12.5 vs. 800), < 0.0001 (12.5 vs. 3200), < 0.0001 (12.5 vs. 50000), 0.0038 (50 vs. 200), < 0.0001 (50 vs. 800), < 0.0001 (50. vs. 3200), < 0.0001 (50 vs. 50000), 0.0002 (200 vs. 800), < 0.0001 (200 vs. 3200), < 0.0001 (200 vs. 50000), 0.9902 (800 vs. 3200), 0.4820 (800 vs. 50000), 0.8638 (3200 vs. 50000). **(b)** Concentration responses of BMP9 were calculated from (a). The BMP9 $EC_{50}$ value was estimated to be 46.1 pg/mL (AAT Bioquest, Inc. Quest Graph™ EC50 Calculator). **(c)** HCT116 cells were transfected with ALK1-specific siRNA (siALK1) or non-targeting control siRNA (siCTRL) with 40 nM for 48 h. Data are presented as the mean ± SEM (n = 4). Statistical comparisons were performed by the unpaired Student's t test (two-sided). **(d)** Transfected cells were incubated with BMP9 of 200 pg/mL or vehicle for 3 h, and then Smad1/5/8 phosphorylation was determined by immunoblotting. Data are presented as mean ± SEM (n = 4, *P < 0.0001 [left], 0.0015 [right], †P = 0.0033). *P < 0.05 versus the corresponding vehicle groups and †P < 0.05 versus BMP9-stimulated siCTRL by the one-way ANOVA with Tukey post-hoc analysis. Source data are provided as a Source Data file.

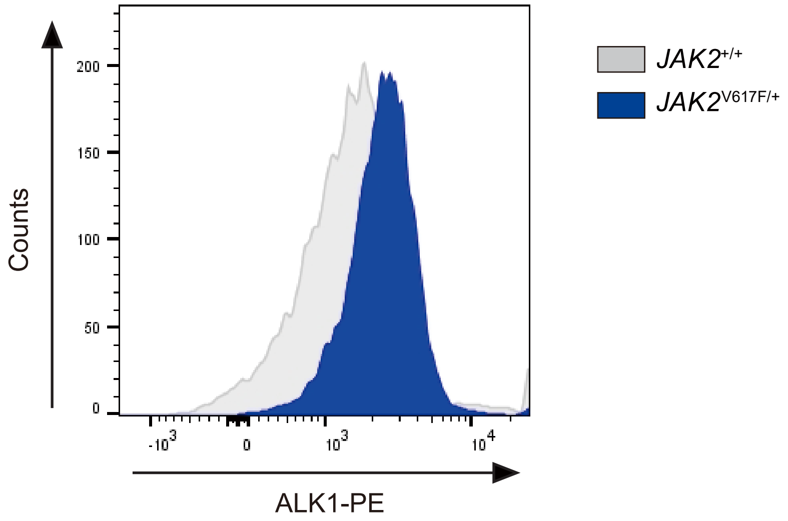


**Supplementary Figure 24. ALK1 expression in *JAK2*$^{V617F/+}$ HCT116 cells by flow cytometry.**
Flow cytometry analysis for ALK1 expression in *JAK2*$^{+/+}$ and *JAK2*$^{V617F/+}$ HCT116 cells. The cells were trypsinized and collected as a single cell suspension, and then stained with an anti-ALK1 antibody followed by a secondary anti-rabbit PE antibody and subjected to the flow cytometry. The representative histogram is shown.

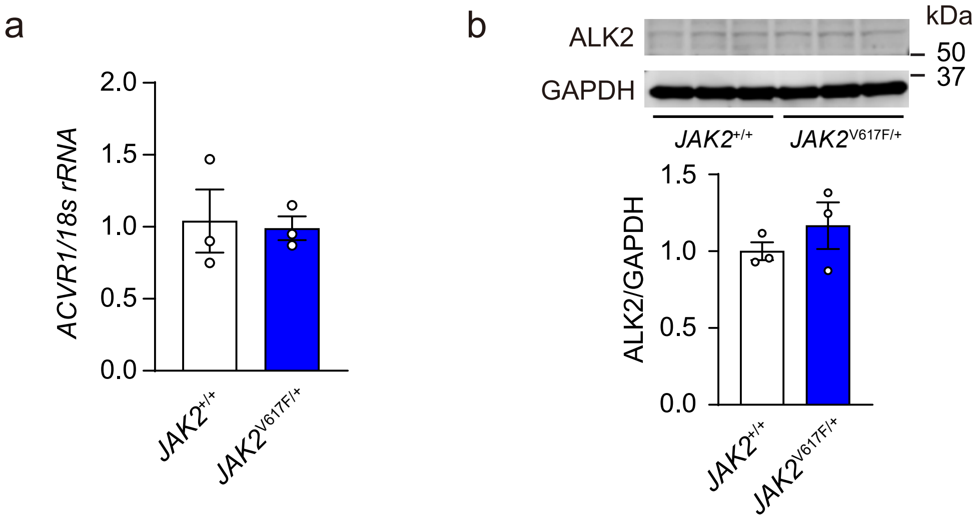


**Supplementary Figure 25. *ACVR1* (ALK2) expressions in *JAK2*$^{V617F/+}$ HCT116 cells.**
**(a)** mRNA expression of *ACVR1* in *JAK2*$^{V617F/+}$ knock-in HCT116 cells. The data were normalized to *18s rRNA* and the average value of *JAK2*$^{+/+}$ cells was set to 1 (n = 3). **(b)** ALK2 protein expression in *JAK2*$^{V617F/+}$ HCT116 cells by Western blot analysis. GAPDH was used for the normalization and the average value of *JAK2*$^{+/+}$ cells was set to 1 (n = 3). The data are presented as mean ± SEM (n = 3). Statistical comparisons were performed by the unpaired Student's t test (two-sided). Source data are provided as a Source Data file.

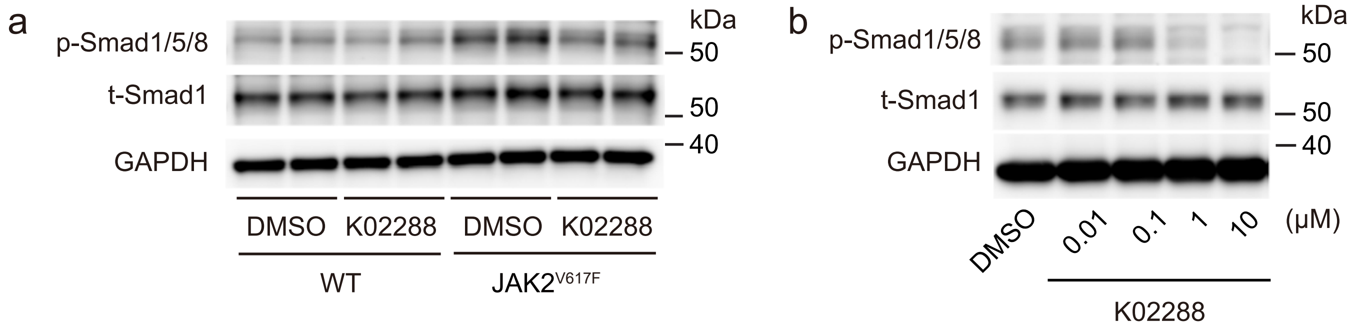


**Supplementary Figure 26. Effects of K02288, an ALK1/2 inhibitor, on the phosphorylation of Smad1/5/8 in the mouse lung and HCT116 cells.**
**(a)** Lung homogenates from the DMSO- or K02288-treated WT mice and JAK2$^{V617F}$ mice 2 weeks after chronic hypoxia (10% $O_2$) were analyzed by immunoblotting with p-Smad1/5/8 and t-Smad1 antibodies. Representative images of two independent experiments are shown. **(b)** *JAK2*$^{V617/+}$ HCT116 cells were incubated with K02288 at the indicated concentrations for 6 h. The cell lysates were subjected to immunoblotting on p-Smad1/5/8 and t-Smad1. p-Smad1/5/8 and t-Smad1 indicate phosphorylated Smad1/5/8 and total Smad1, respectively. Representative images of two independent experiments are shown. GAPDH was used as the loading control. WT, wild-type mice; JAK2$^{V617F}$, JAK2V617F-expressing transgenic mice. Source data are provided as a Source Data file.

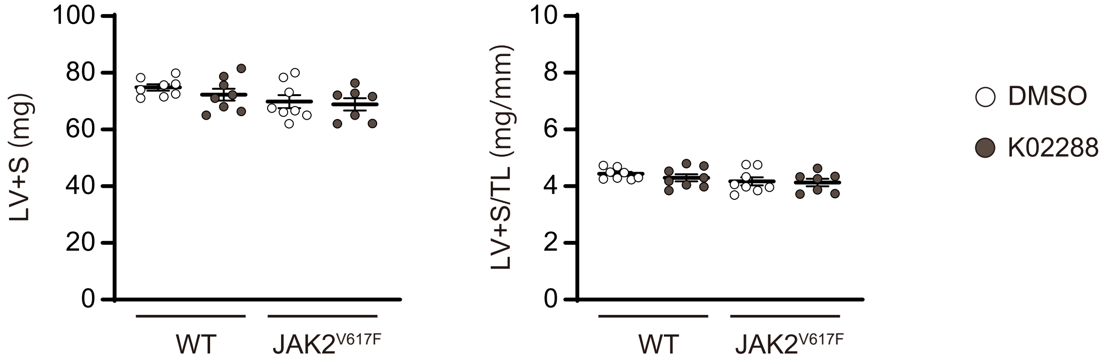


**Supplementary Figure 27. Left ventricular weight in K02288-treated JAK2$^{V617F}$ mice after exposure to chronic hypoxia.** Left ventricular (LV) weight including septum (S) was measured after exposure to normoxia (21% $O_2$) or chronic hypoxia (10% $O_2$) for 2 weeks (n = 8, 8, 8, 7 in each). LV+S was normalized by tibia length (TL). All data are presented as mean ± SEM. The statistical comparison was performed by the one-way ANOVA. Source data are provided as a Source Data file.

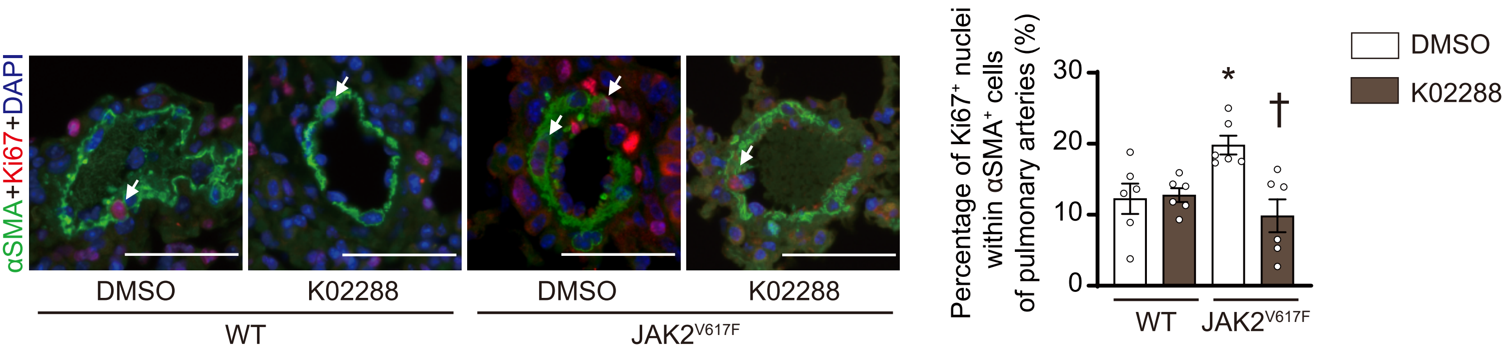


**Supplementary Figure 28. K02288, an ALK1/2 inhibitor, attenuates chronic hypoxia-induced proliferation of pulmonary arterial smooth muscle cells in JAK2$^{V617F}$ mice.**

Left, triple-labeled immunofluorescent staining (αSMA, green; Ki67, red; DAPI, blue) of the lung sections in DMSO- or K02288-treated WT mice and JAK2$^{V617F}$ mice 2 weeks after chronic hypoxia. White arrows indicate Ki67-positive nuclei within αSMA$^+$ cells. Scale bars, 50 μm. Right, quantitative analyses of the percentage of Ki67-positive nuclei within αSMA$^+$ cells of distal pulmonary arteries with a diameter of 50-100 μm (n = 6). More than 80 αSMA$^+$ cells were counted in each section. Data are presented as mean ± SEM. $^*$P = 0.0330 versus the corresponding WT mice and $^†$P = 0.0040 versus DMSO-treated JAK2$^{V617F}$ mice by the one-way ANOVA with Tukey post-hoc analysis. Source data are provided as a Source Data file.

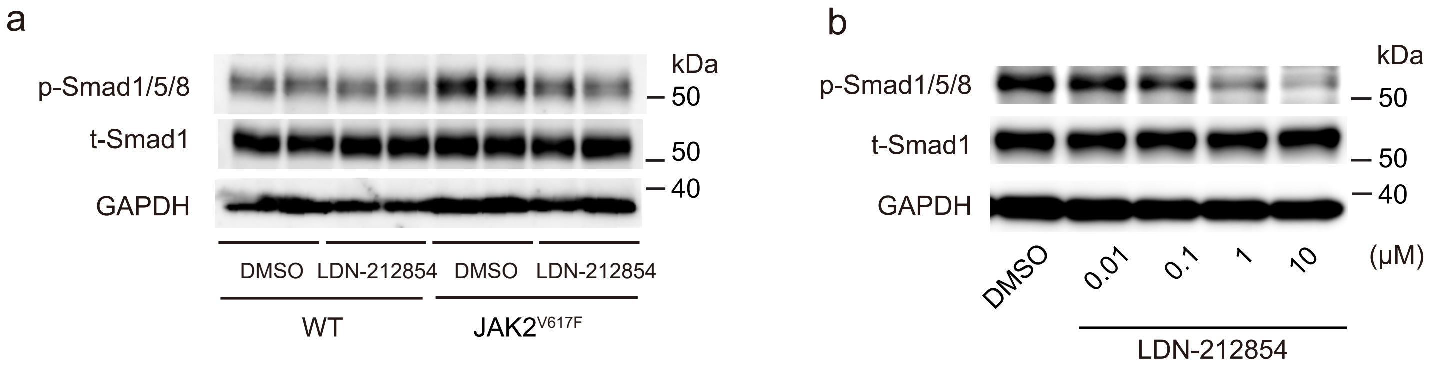


**Supplementary Figure 29. Effects of LDN-212854, an ALK1/2 inhibitor, on the phosphorylation of Smad1/5/8 in the mouse lungs and HCT116 cells.**

**(a)** Lung homogenates from the DMSO- or LDN-212854-treated WT mice and JAK2$^{V617F}$ mice after chronic hypoxia (10% $O_2$) for 2 weeks were analyzed by immunoblotting with p-Smad1/5/8 and t-Smad1 antibodies. Representative images of two independent experiments are shown. **(b)** *JAK2*$^{V617/+}$ HCT116 cells were incubated with LDN-212854 at the indicated concentrations for 6 h. The cell lysates were subjected to immunoblotting on p-Smad1/5/8 and t-Smad1. p-Smad1/5/8 and t-Smad1 indicate phosphorylated Smad1/5/8 and total Smad1, respectively. Representative images of two independent experiments are shown. GAPDH was used as the loading control. WT, wild-type mice; JAK2$^{V617F}$, JAK2V617F-expressing transgenic mice. Source data are provided as a Source Data file.

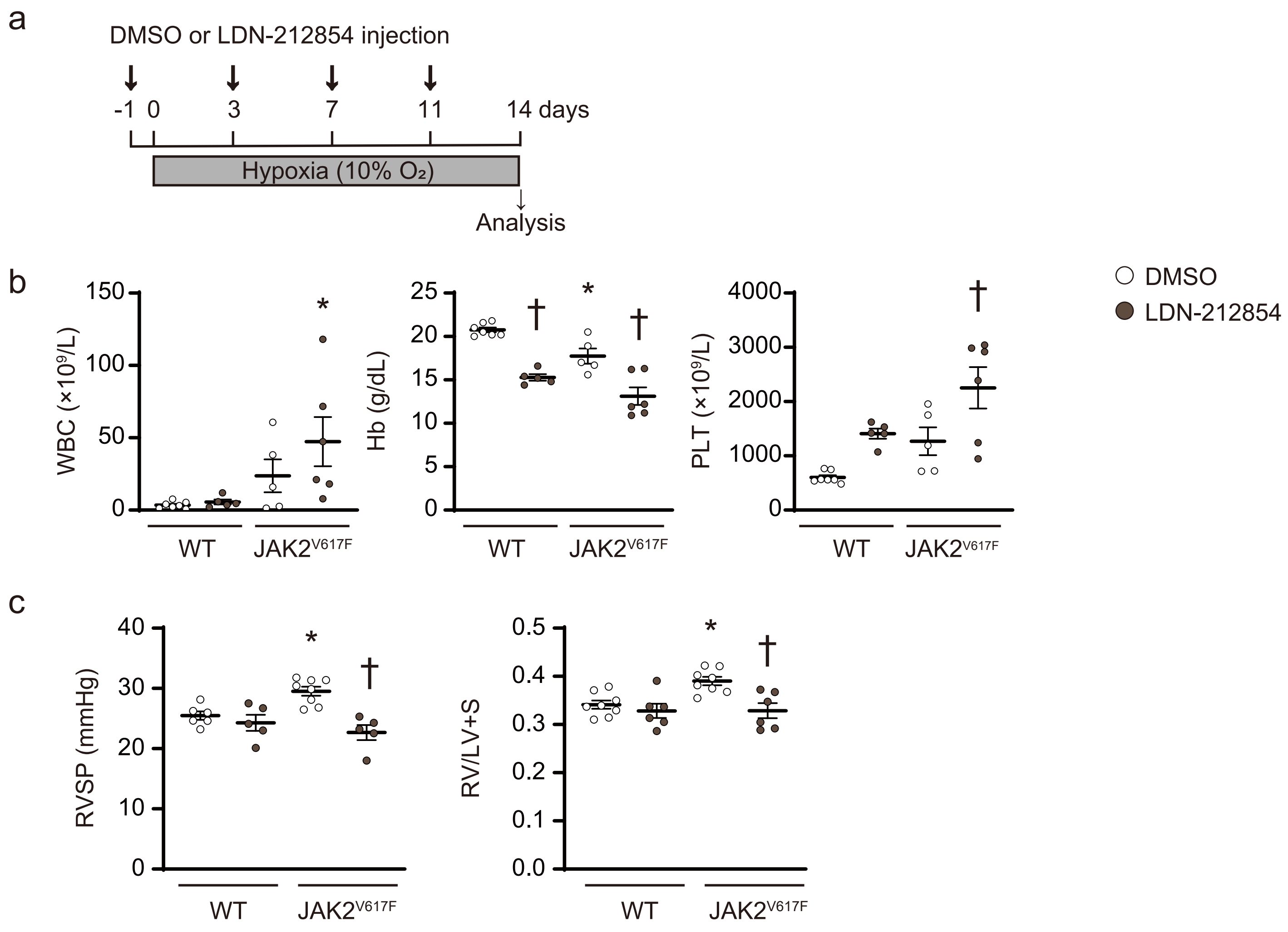


**Supplementary Figure 30. LDN-212854, an ALK1/2 inhibitor, improves chronic hypoxia-induced pulmonary hypertension in JAK2$^{V617F}$ mice.**

**(a)** Schematic protocol. Vehicle (DMSO) or LDN-212854 was administered via an intraperitoneal injection of 9 mg/kg body weight during 2-week chronic hypoxia-exposure as indicated. **(b)** Peripheral blood cell counts in DMSO- or LDN-212854-treated WT mice and JAK2$^{V617F}$ mice after exposure to chronic hypoxia for 2 weeks (n = 7, 5, 5, 6, *P = 0.0496 for WBC, n = 7, 5, 5, 6, *P < 0.0001, †P = 0.0261 [left], 0.0010 [right] for Hb, n = 7, 5, 5, 6, †P = 0.0403 for PLT). **(c)** RVSP and RV hypertrophy determined by RV/LV+S in DMSO- or LDN-212854-treated WT mice and JAK2$^{V617F}$ mice (n = 6, 5, 8, 5, *P = 0.0249, †P = 0.0003 for RVSP, n =8, 6, 8, 6, *P = 0.0197, †P = 0.0054 for RV/LV+S). Data are presented as mean ± SEM. *P < 0.05 versus the corresponding WT mice and †P < 0.05 versus DMSO-treated JAK2$^{V617F}$ mice by the one-way ANOVA with Tukey post-hoc analysis. Source data are provided as a Source Data file.

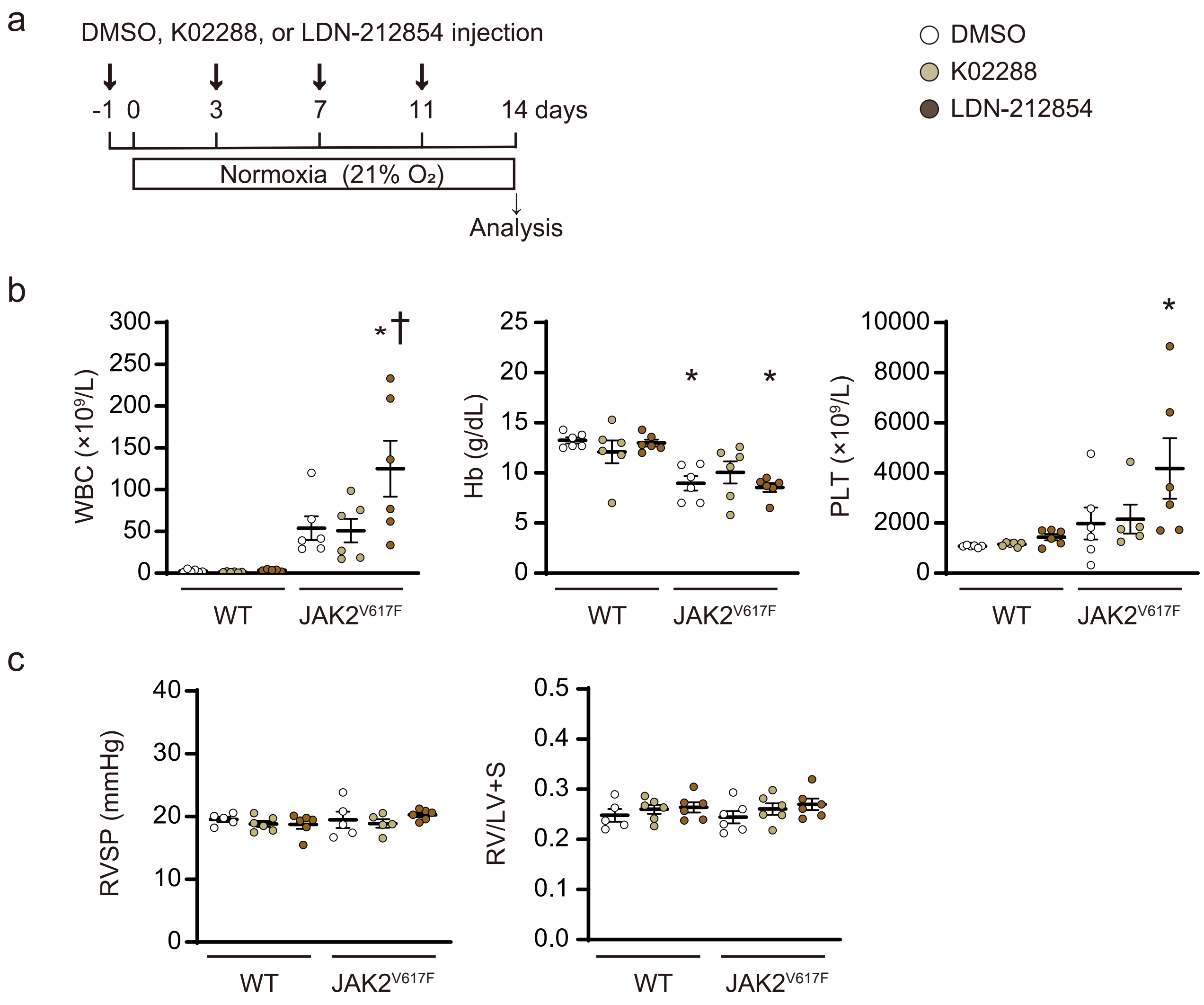


**Supplementary Figure 31. No effects of ALK1/2 inhibitors of K02288 or LDN-212854 on pulmonary hypertension in WT mice and JAK2$^{V617F}$ mice under normoxia.**

**(a)** Schematic protocol. Vehicle (DMSO, 12 mg/kg), K02288 (12 mg/kg), or LDN-212854 (9 mg/kg) was administered via an intraperitoneal injection during 2-week normoxia-exposure as indicated. **(b)** Peripheral blood cell counts in DMSO-, K02288-, or LDN-212854-treated WT mice and JAK2$^{V617F}$ mice after exposure to normoxia for 2 weeks (n = 6, 6, 5, 6, 6, 6, *P = 0.0003, $^{\dagger}$P = 0.0440 for WBC, n =6 in each group, *P = 0.0045 [left], 0.0031 [right] for Hb, n = 6, 6, 6, 6, 5, 6, *P = 0.0340 for PLT). **(c)** RVSP and RV hypertrophy determined by RV/LV+S in DMSO-, K02288-, or LDN-212854-treated WT mice and JAK2$^{V617F}$ mice (n = 5, 6, 6, 5, 5, 6 for RVSP, n = 5, 6, 6, 6, 6, 6 for RV/LV+S). Data are presented as mean ± SEM. *P < 0.05 versus the corresponding WT mice and $^{\dagger}$P < 0.05 versus DMSO-treated JAK2$^{V617F}$ mice by the one-way ANOVA with Tukey post-hoc analysis. WT, wild-type mice; JAK2$^{V617F}$, JAK2V617F-expressing transgenic mice. Source data are provided as a Source Data file.

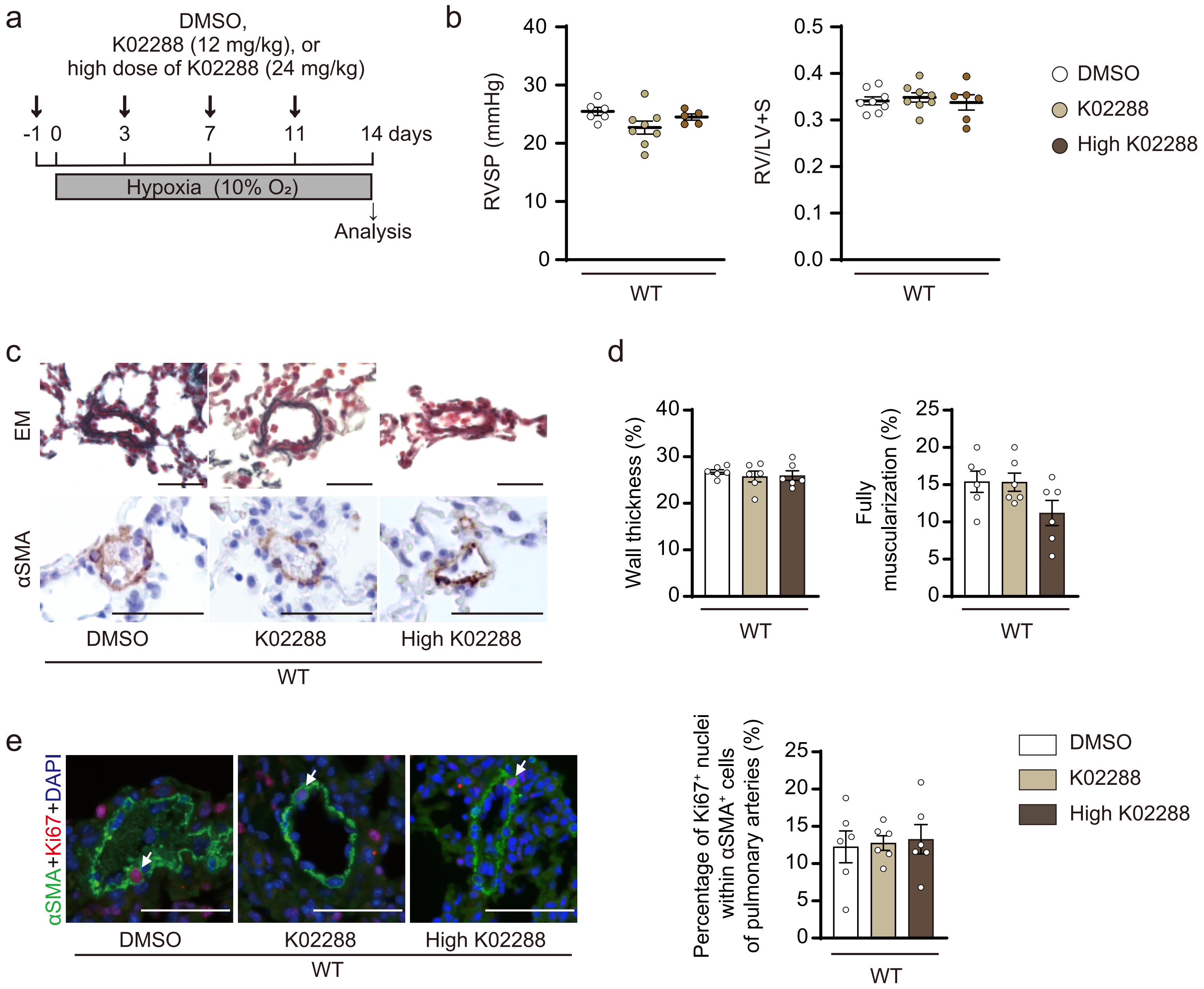


**Supplementary Figure 32. Effects of a high dose of K02288, an ALK1/2 inhibitor, on pulmonary hypertension in WT mice after chronic hypoxia.**

**(a)** Schematic representation. Vehicle (DMSO, 12 mg/kg), K02288 (12 mg/kg), or high dose of K02288 (24 mg/kg) was administered via an intraperitoneal injection during 2-week chronic hypoxia-exposure as indicated. The data of DMSO- and K02288 (12 mg/kg)-treated WT mice are from the main Fig. 8 for comparison. **(b)** RVSP (n = 6, 8, 5) and RV hypertrophy determined by RV/LV+S (n = 8, 8, 6). **(c)** Representative images of EM-stained sections and sections immunostained with an anti-αSMA antibody. Scale bar, 25 μm. **(d)** Quantitative analysis of medial wall thickness in EM-stained sections (left, n = 6 in each group) and the percentage of muscularized distal pulmonary arteries in αSMA-immunostained sections (right, n = 6 in each group). **(e)** Left, representative images of triple-labeled immunofluorescent staining (αSMA, green; Ki67, red; DAPI, blue). White arrows indicate Ki67-positive nuclei within αSMA$^+$ cells. Scale bars, 50 μm. Right, quantitative analyses of the percentage of Ki67-positive nuclei within αSMA$^+$ cells of distal pulmonary arteries with a diameter of 50-100 μm (n = 6 in each group). More than 80 αSMA$^+$ cells were counted in each section. All data are presented as mean ± SEM. The statistical comparison was performed by the one-way ANOVA. Source data are provided as a Source Data file.

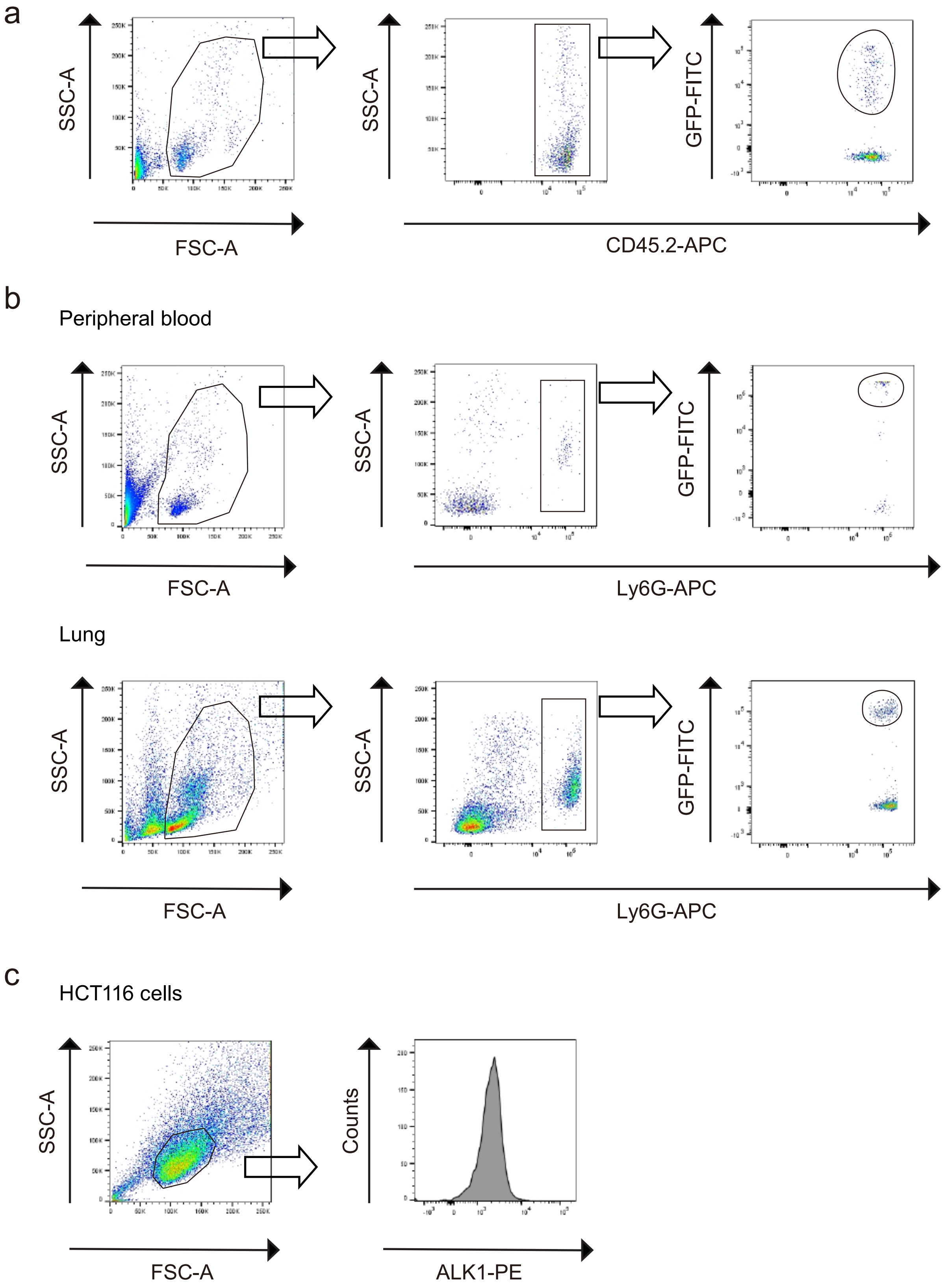


**Supplementary Figure 33. Gating strategy used in the present study.**

**(a)** Gating strategy used to analyze the chimerism in the peripheral blood. The percentages of $GFP^+$ cells in the circulating $CD45.2^+$ cells are shown in Figure 4b and Supplementary Figure 16. **(b)** Gating strategy used to analyze the percentages of $GFP^+$ cells in $Ly6G^+$ cells in comparison to the peripheral blood and lungs. Data are shown in Figure 4c and 4d. **(c)** Gating strategy used to analyze ALK1 expressions in HCT116 cells. Data are shown in Supplementary Figure 24.

**Supplementary Table 1. Comparisons of the presence of *JAK2*V617F between control subjects and patients with PH.**

| | Control subjects (n = 83) | Patients with PH (n = 70) | P value |
|---|---|---|---|
| Age, years | 59 ± 17 | 59 ± 15 | 0.904 |
| Female, n (%) | 45 (54) | 47 (67) | 0.104 |
| Presence of *JAK2*V617F, n (%) | 0 | 5 (7.1) | 0.019 |

Values are mean ± SD or number (%). PH, pulmonary hypertension. Comparisons of means between the two groups were performed by the unpaired Student's t-test (two-sided). Categorical variables were compared using Chi-square test (two-sided) or Fisher's exact test (two-sided).

**Supplementary Table 2. The cases of PH patients with *JAK2*V617F-positive clonal hematopoiesis.**

| Case | Age (years) | Gender (M/F) | Group of PH | *JAK2*V617F allele frequency (%) | WBC ($\times 10^9$/L) | Hb (g/dL) | PLT ($\times 10^9$/L) | Mean PAP (mmHg) | PVR (wood, unit) |
|---|---|---|---|---|---|---|---|---|---|
| 1 | 60s | F | IV | 14.90 | 8.1 | 12.7 | 360 | 25 | 3.6 |
| 2 | 50s | F | I | 0.54 | 5.6 | 12.9 | 212 | 58 | 8.1 |
| 3 | 30s | F | I | 0.06 | 7.8 | 12.8 | 236 | 64 | 21.1 |
| 4 | 60s | F | IV | 16.08 | 7.2 | 13.6 | 339 | 57 | 14.9 |
| 5 | 70s | M | IV | 70.96 | 8.7 | 11.4 | 206 | 41 | 4.2 |

Group category is defined by the WHO classification of PH; Group I, pulmonary arterial hypertension; Group IV, chronic thromboembolic pulmonary hypertension. WBC, white blood cell count; Hb, hemoglobin concentration; PLT, platelet count; PAP, pulmonary arterial pressure; PVR, pulmonary vascular resistance.

**Supplementary Table 3. Comparison of the PH patients with and without *JAK2*V617F.**

| | *JAK2*V617F positive (n = 5) | *JAK2*V617F negative (n = 65) | P value |
|---|---|---|---|
| Age, years | 58 ± 15 | 59 ± 15 | 0.902 |
| Female, n (%) | 4 (80) | 43 (66) | 1.000 |
| Group of PH, I/II/III/VI/V, n (%) | 2 (40) / 0 (0) / 0 (0) / 3 (60) / 0 (0) | 30 (46) / 0 (0) / 9 (14) / 24 (37) / 2 (3) | NA |
| NYHA functional class, I/II/III/VI, n (%) | 0 (0) / 3 (60) / 2 (40) / 0 (0) | 9 (14) / 29 (45) / 23 (35) / 4 (6) | NA |
| Laboratory data | | | |
| WBC, $\times 10^9$/L | 7.4 ± 1.1 | 6.4 ± 2.2 | 0.287 |
| Hb, g/dL | 12.6 ± 0.7 | 13.6 ± 2.2 | 0.331 |
| Hematocrit, % | 38.7 ± 7.9 | 41.4 ± 6.4 | 0.384 |
| PLT, $\times 10^9$/L | 270 ± 73 | 221 ± 78 | 0.118 |
| Total bilirubin, mg/dL | 0.88 ± 0.43 | 0.97 ± 0.62 | 0.767 |
| Aspartate aminotransferase, IU/L | 25.8 ± 11.7 | 27.6 ± 13.6 | 0.779 |
| Lactate dehydrogenase, IU/L | 262 ± 82 | 246 ± 91 | 0.697 |
| Creatinine | 0.83 ± 0.27 | 0.82 ± 0.29 | 0.917 |
| Estimated GFR, mL/min/1.73 $m^2$ | 67.0 ± 26.7 | 70.7 ± 26.6 | 0.763 |
| Serum iron, μg/dL | 57 ± 29 | 85 ± 57 | 0.276 |
| Ferritin, ng/mL | 64 ± 64 | 117 ± 180 | 0.518 |
| Uric acid, mg/dL | 6.3 ± 2.5 | 6.1 ± 1.8 | 0.785 |
| C-reactive protein, mg/dL | 1.2 ± 1.8 | 0.7 ± 1.4 | 0.484 |
| B-type natriuretic peptide, pg/mL | 44.9 (33.1 – 542.7) | 135 (40.8 – 291.6) | 0.511 |
| Echocardiography | | | |
| Left ventricular ejection fraction, % | 68.2 ± 5.4 | 63.1 ± 12.0 | 0.348 |
| RV end-diastolic area, $cm^2$ | 25.6 ± 10.3 | 25.1 ± 12.0 | 0.931 |
| RV fractional area change, % | 27.2 ± 9.1 | 32.4 ± 14.2 | 0.429 |
| TR-PG, mmHg | 66.4 ± 16.0 | 64.3 ± 25.6 | 0.864 |
| Hemodynamics | | | |
| Mean PAP, mmHg | 49 ± 15 | 44 ± 13 | 0.422 |
| Mean PAWP, mmHg | 16 ± 8 | 11 ± 5 | 0.261 |
| Cardiac index, L/min/$m^2$ | 2.7 ± 0.4 | 2.6 ± 0.8 | 0.839 |
| PVR, wood unit | 10.3 ± 7.4 | 9.4 ± 5.3 | 0.688 |

Data are presented as mean ± SD, number (%) or median (inter-quartile range). The patients were classified into 5 groups according to the WHO clinical classification of PH; Group I, pulmonary arterial hypertension; Group II, pulmonary hypertension due to left heart disease; Group III, pulmonary hypertension due to lung diseases and/or hypoxia; Group IV, chronic thromboembolic pulmonary hypertension; Group V, pulmonary hypertension with unclear multifactorial mechanisms. NA, not applicable; NYHA, New York Heart Association; WBC, white blood cell count; Hb, hemoglobin concentration; PLT, platelet count; GFR, glomerular filtration rate; RV, right ventricular; TR-PG, tricuspid regurgitation pressure gradient; PAP, pulmonary arterial pressure; PAWP, pulmonary arterial wedge pressure; PVR, pulmonary vascular resistance. Comparisons of values between the two groups were performed by the unpaired Student's t-test (two-sided) or Mann-Whitney U-test (two-sided). Categorical variables were compared using Fisher's exact test (two-sided).

**Supplementary Table 4. Primers used for RT-qPCR.**

| | Gene | | | Sequences | |
|---|---|---|---|---|---|
| Mouse | *Ccl2* | Forward | 5’- | GGCTCAGCCAGATGCAGTTAAC | -3’ |
| | | Reverse | 5’- | GCCTACTCATTGGGATCATCTTG | -3’ |
| | *Cxcl1* | Forward | 5’- | ACTCAAGAATGGTCGCGAGG | -3’ |
| | | Reverse | 5’- | ACTTGGGGACACCTTTTAGCA | -3’ |
| | *Ccr1* | Forward | 5’- | TTAGCTTCCATGCCTGCCTTATA | -3’ |
| | | Reverse | 5’- | TCCACTGCTTCAGGCTCTTGT | -3’ |
| | *Cxcr2* | Forward | 5’- | TCGTAGAACTACTGCAGGATTAAG | -3’ |
| | | Reverse | 5’- | GGGACAGCATCTGGCAGAATA | -3’ |
| | *Pdgfrb* | Forward | 5’- | ACTACATCTCCAAAGGCAGCACCT | -3’ |
| | | Reverse | 5’- | TGTAGAACTGGTCGTTCATGGGCA | -3’ |
| | *Tgfb1* | Forward | 5’- | AGCTGCGCTTGCAGAGATTA | -3’ |
| | | Reverse | 5’- | AGCCCTGTATTCCGTCTCCT | -3’ |
| | *Acvrl1* | Forward | 5’- | GGCCTTTGGCCTAGTGCTAT | -3’ |
| | | Reverse | 5’- | GGAGAGGACCGGATCTGC | -3’ |
| | *Acvr1* | Forward | 5’- | CGCTTCAGACATGACCTCCA | -3’ |
| | | Reverse | 5’- | CCGAAGGCAGCTAACCGTAT | -3’ |
| | *Bmpr2* | Forward | 5’- | GGATGGCAGCAGTATACAGATAGG | -3’ |
| | | Reverse | 5’- | CGCCACCGCTTAAGAGAGTAT | -3’ |
| | *18s rRNA* | Forward | 5’- | GTCTGTGATGCCCTTAGATG | -3’ |
| | | Reverse | 5’- | AGCTTATGACCCGCACTTAC | -3’ |
| Human | *ACVRL1* | Forward | 5’- | CCATCGTGAATGGCATCGTG | -3’ |
| | | Reverse | 5’- | GAGGGGTTTGGGTACCAGCA | -3’ |
| | *ACVR1* | Forward | 5’- | GAAGGGCTCATCACCACCAA | -3’ |
| | | Reverse | 5’- | CCATACCTGCCTTTCCCGAC | -3’ |
| | *18s rRNA* | Forward | 5’- | GTAACCCGTTGAACCCCATT | -3’ |
| | | Reverse | 5’- | CCATCCAATCGGTAGTAGCG | -3’ |

**Supplementary Table 5. Primers used for ChIP-qPCR.**

| Gene | Sequences | | | |
|---|---|---|---|---|
| *ACVRL1* TSS-875bp | Forward | 5’- | CCTGCCGGTATGAAGCCATT | -3’ |
| | Reverse | 5’- | ACAGTCAGGATGGAGGGACA | -3’ |
| *ACVRL1* TSS-1660bp | Forward | 5’- | TTGGGTGTGTCAGGGTTCTG | -3’ |
| | Reverse | 5’- | AGGAATAGAGGCTGGGGGAG | -3’ |

**Supplementary Table 6. Primers and probes used for allele-specific qPCR.**

| Primers and probes | | Sequences | | |
|---|---|---|---|---|
| *JAK2* | Forward | 5’- | CTTTCTTTGAAGCAGCAAGTATGA | -3’ |
| *JAK2*wild-type | Reverse | 5’- | GTAGTTTTACTTACTCTCGTCTCCACATAC | -3’ |
| *JAK2*V617F | Reverse | 5’- | GTAGTTTTACTTACTCTCGTCTCCACATAA | -3’ |
| *JAK2* | Probe | 5’- | FAM-TGAGCAAGCTTTCTCACAAGCATTTGGTTT-TAMRA | -3’ |
