# Supplementary material for: Clonal hematopoiesis with JAK2V617F promotes pulmonary hypertension with ALK1 upregulation in lung neutrophils
Source: Nat Commun. 2021 Oct 26;12:6177. doi: 10.1038/s41467-021-26435-0 (PMC8548396; doi:10.1038/s41467-021-26435-0)
Supplement: Supplementary file 3 — Description of Additional Supplementary Files [file 41467_2021_26435_MOESM3_ESM.docx]

**Description of Additional Supplementary Files**

File Name: Supplementary Data 1

Description: RNA sequence data from lineage^-^Sca1^+^Kit^+^ (LSK) cells in the bone marrow (BM), Ly6G^+^ cells in the BM, Ly6G^+^ cells in the peripheral blood (PB), and Ly6G^+^ cells in the lung in JAK2^V617F^ mice in comparison to wild-type (WT) mice.
