# Supplementary material for: Clonal hematopoiesis with JAK2V617F promotes pulmonary hypertension with ALK1 upregulation in lung neutrophils
Source: Nat Commun. 2021 Oct 26;12:6177. doi: 10.1038/s41467-021-26435-0 (PMC8548396; doi:10.1038/s41467-021-26435-0)
Supplement: Supplementary file 4 — Supplementary Data 1 [file 41467_2021_26435_MOESM4_ESM.docx]

**Supplemental data of RNA sequence**

**Upregulated genes in the Ly6G^+^ neutrophils from JAK2^V617F^ mice (>1.5 fold in the lung in comparison to WT mice) in RNA sequence.**

| Symbol | Lung | PB | BM | LSK |
| --- | --- | --- | --- | --- |
| GGT1 | 35.223 | 26.69 | 6.642 | -1.226 |
| Ngp | 28.732 | 7.899 | 1.045 | 1.96 |
| CAMP | 25.887 | 5.197 | -1.042 | 1.634 |
| GPR27 | 21.239 | 7.72 | 1.105 | -1.119 |
| LTF | 20.574 | 7.377 | 1.007 | 1.512 |
| LIPG | 17.288 | 16.129 | 19.897 | 1.108 |
| CD177 | 17.144 | 6.414 | 1.18 | 1.775 |
| ADPGK | 14.165 | 6.585 | 1.247 | 1.022 |
| Orm1 (includes others) | 14 | 6.141 | -1.39 | 1.603 |
| Ifitm6 | 11.792 | 5.467 | 1.915 | 2.968 |
| Itgb2l | 11.736 | 5.062 | -1.068 | 1.417 |
| QSOX1 | 10.235 | 4.906 | 3.175 | 1.212 |
| UPP1 | 8.577 | 13.342 | 5.847 | 1.001 |
| FLOT2 | 8.504 | 7.117 | 1.6 | 1.717 |
| Ppbp | 8.501 | -1.425 | -4.139 | -1.133 |
| LCN2 | 8.268 | 3.46 | 1.163 | 4.977 |
| MGST2 | 7.999 | 3.126 | -1.33 | 1.079 |
| SERPINB1 | 7.403 | 3.737 | 1.402 | 1.343 |
| FCN1 | 7.155 | 1.723 | 1.313 | 1.646 |
| Stfa3 | 7.123 | 2.677 | 1.564 | 1.936 |
| Ifitm1 | 7.06 | 7.194 | 11.561 | 3.014 |
| ACVRL1 | 6.395 | 4.777 | 1.458 | 1.196 |
| TGM1 | 6.357 | 5.918 | 3.849 | 2.775 |
| CLDN15 | 6.328 | 2.65 | 1.143 | 1.761 |
| TSPO | 5.934 | 4.631 | 3.133 | 2.059 |
| MCAM | 5.655 | 4.822 | 2.571 | -1.048 |
| GNB5 | 5.65 | 6.465 | 4.616 | 1.321 |
| Ly6a (includes others) | 5.189 | 7.038 | 1.305 | 2.406 |
| GYG1 | 5.13 | 5.998 | 1.674 | 1.05 |
| GCLM | 5.079 | -4.765 | 1.973 | 1.042 |
| BCL2L1 | 5.04 | -6.062 | 2.643 | 1.024 |
| ATXN10 | 5.025 | 4.01 | 1.337 | -1.106 |
| FAM20C | 5.017 | 10.343 | 15.518 | -1.005 |
| ATP11A | 4.897 | 3.651 | 1.507 | 1.255 |
| MMP8 | 4.848 | 2.496 | -1.173 | 2.285 |
| Retnlg | 4.754 | 2.41 | -1.129 | 2.482 |
| ANKRD22 | 4.726 | 2.784 | -1.415 | 1.063 |
| TMEM40 | 4.682 | 2.067 | 1.039 | -1.084 |
| OAS1 | 4.662 | 4.814 | 2.917 | -1.601 |
| GP1BB | 4.653 | 1.35 | -1.607 | 1.553 |
| TLR1 | 4.633 | 5.494 | 6.103 | 1.226 |
| MMP25 | 4.598 | 3.215 | 1.797 | 1.061 |
| EPS8 | 4.572 | 11.365 | 12.087 | 2.227 |
| STEAP4 | 4.552 | 5.219 | 13.462 | 1.033 |
| MOV10 | 4.552 | 3.67 | 1.805 | -1.497 |
| MAPK13 | 4.512 | 3.518 | 1.166 | 1.181 |
| GALNT6 | 4.441 | 6.566 | 4.116 | 1.355 |
| ZBP1 | 4.419 | 3.899 | 2.366 | -3.415 |
| S100A8 | 4.415 | 2.358 | 1.183 | 1.565 |
| AMBP | 4.402 | 5.826 | 3.33 | 1.094 |
| IGFBP6 | 4.399 | 7.805 | 16.406 | 1.268 |
| ASNSD1 | 4.379 | 3.438 | 1.596 | 1.003 |
| LBP | 4.323 | 2.546 | 1.135 | 1.342 |
| SCAMP1 | 4.227 | 2.137 | 1.842 | -1.05 |
| ANXA1 | 4.047 | 3.065 | 1.129 | -1.048 |
| CDC25B | 4.045 | -5.888 | 2.871 | 1.202 |
| Gm19705 | 4.013 | 3.935 | 1.03 | 1.47 |
| HMOX1 | 4.008 | 1.312 | -3.217 | -3.643 |
| PLSCR1 | 3.856 | 4.033 | 2.224 | 2.153 |
| FPR2 | 3.838 | 2.553 | 1.547 | -1.102 |
| B230208H11Rik | 3.81 | 1.967 | -1.313 | -1.386 |
| FMO5 | 3.796 | 1.858 | 1.586 | -1.478 |
| CD38 | 3.787 | 2.158 | 3.293 | 1.714 |
| TINAGL1 | 3.779 | 2.401 | -1.238 | -1.476 |
| KIT | 3.758 | 7.531 | 1.647 | -1.419 |
| SYNE1 | 3.753 | 3.15 | -1.459 | -1.199 |
| G6PD | 3.708 | 2.64 | 1.781 | 1.395 |
| S100A9 | 3.697 | 2.183 | 1.081 | 1.757 |
| DSTN | 3.677 | 3.26 | 1.034 | -1.143 |
| ANXA3 | 3.673 | 2.561 | -1.067 | 1.725 |
| TNFRSF18 | 3.647 | 3.635 | 2.431 | -1.523 |
| S100A6 | 3.637 | 2.351 | 1.768 | 3.299 |
| KLHDC4 | 3.572 | 2.756 | 1.394 | 1.106 |
| CIB2 | 3.561 | 3.257 | 1.343 | -1.725 |
| CKAP4 | 3.553 | 2.787 | 1.171 | -1.14 |
| LTB4R | 3.519 | 2.949 | 1.147 | 1.426 |
| SPP1 | 3.493 | -1.19 | 1.652 | 1.396 |
| PGLYRP1 | 3.469 | 2.11 | 1.282 | 2.062 |
| ANKRD37 | 3.428 | 3.84 | 9.409 | -1.312 |
| RAB3D | 3.413 | 2.007 | -1.09 | 1.187 |
| LY6G5B | 3.378 | 1.847 | -1.415 | -1.366 |
| DGAT1 | 3.374 | 2.303 | 1.151 | 1.374 |
| HSD11B1 | 3.362 | 3.03 | -1.069 | 1.188 |
| LMO1 | 3.349 | -1.04 | -1.001 | 1.233 |
| GOLIM4 | 3.325 | 1.644 | -1.413 | 1.086 |
| PDK3 | 3.249 | 2.364 | 1.131 | 1.393 |
| SLC25A24 | 3.187 | 2.321 | 1.165 | -1.073 |
| DEPDC1B | 3.176 | 2.002 | -1.185 | 1.037 |
| WIPI1 | 3.174 | -1.642 | 1.115 | 1.772 |
| CASP6 | 3.171 | 3.678 | 1.916 | -1.18 |
| C1RL | 3.169 | 3.824 | 1.459 | 1.117 |
| PPP1R42 | 3.157 | 1.286 | -1.412 | -1.092 |
| ZDHHC3 | 3.156 | 2.435 | 1.37 | 1.291 |
| SRI | 3.15 | 2.898 | 1.371 | -1.124 |
| ENO1 | 3.149 | 2.835 | 2.181 | -1.113 |
| LRG1 | 3.128 | 2.8 | 3.396 | 2.44 |
| TNNT1 | 3.115 | 1.197 | -1.17 | 1.171 |
| FPR1 | 3.111 | 2.139 | 1.201 | 1.087 |
| SLC17A9 | 3.111 | 1.419 | 1.511 | -1.016 |
| TARM1 | 3.108 | 2.448 | 2.089 | 1.387 |
| PGK1 | 3.102 | 2.605 | 1.102 | 1.3 |
| LTA4H | 3.093 | 2.407 | 1.007 | 1.469 |
| ESD | 3.056 | 2.1 | 1.506 | -1.116 |
| TST | 3.052 | 1.373 | -1.494 | -1.246 |
| CDADC1 | 3.051 | 1.29 | -1.826 | 1.012 |
| ARL2BP | 3.041 | 1.111 | 1.205 | 1.09 |
| C19orf12 | 3.019 | 2.988 | 1.792 | 1.019 |
| DEF8 | 3.018 | 2.245 | 1.727 | -1.146 |
| TFDP1 | 3.009 | 2.625 | -1.156 | 1.438 |
| TREML2 | 3.003 | 1.351 | 1.422 | -1.103 |
| TREML1 | 2.993 | -1.473 | -2.42 | 1.074 |
| WI2-2373I1.2 | 2.99 | 3.003 | 3.84 | 1.435 |
| Gm15350 | 2.975 | 2.169 | 1.892 | -1.753 |
| ZMPSTE24 | 2.963 | 1.499 | 1.016 | 1.221 |
| METTL9 | 2.963 | 2.342 | 1.111 | -1.21 |
| SLC25A38 | 2.957 | 3.695 | 1.554 | 1.034 |
| EIF4E3 | 2.956 | 2.409 | 1.217 | 1.18 |
| Prr13 | 2.947 | 2.312 | 1.602 | -1.043 |
| GLRX | 2.945 | 2.303 | 1.297 | -1.213 |
| PLIN2 | 2.931 | 2.405 | 1.609 | 1.023 |
| APP | 2.915 | 3.01 | 1.857 | 2.096 |
| PRELID1 | 2.912 | 2.597 | 1.859 | 1.142 |
| Neat1 | 2.908 | -1.83 | 1.12 | 1.561 |
| GAPDH | 2.9 | 2.492 | 1.386 | 1.217 |
| C130026I21Rik (includes others) | 2.898 | 1.971 | 1.151 | -1.236 |
| GYS1 | 2.871 | 2.078 | 1.231 | 1.008 |
| OAZ2 | 2.869 | 2.207 | 1.756 | -1.1 |
| MRGPRX3 | 2.868 | 2.73 | 1.477 | 3.365 |
| TBC1D2 | 2.859 | 2.57 | 1.771 | 1.13 |
| TMEM120A | 2.856 | 2.19 | 1.203 | 1.403 |
| PRDX5 | 2.845 | 2.451 | 1.081 | 1.01 |
| LDHA | 2.841 | 2.195 | 1.569 | 1.522 |
| ASB7 | 2.84 | 2.455 | 1.619 | 1.166 |
| BLCAP | 2.839 | 1.869 | 1.558 | -1.27 |
| IL18BP | 2.833 | 2.817 | -1.166 | 1.26 |
| S1PR4 | 2.833 | 1.663 | -1.011 | -1.221 |
| DHRS1 | 2.822 | 2.218 | -1.03 | 1.063 |
| C15orf48 | 2.815 | 2.203 | 1.21 | 2.464 |
| SLFN12L | 2.792 | 2.076 | 1.307 | 1.13 |
| OLFML2B | 2.778 | 2.094 | -1.103 | 1.008 |
| VIM | 2.772 | 3.141 | 1.261 | -1.03 |
| RHOV | 2.771 | 1.865 | 2.669 | 1.073 |
| ANO10 | 2.768 | 1.915 | -1.12 | -1.444 |
| GCA | 2.758 | 2.385 | 1.765 | 1.198 |
| MIF | 2.755 | 3.275 | 3.693 | 1.103 |
| TKT | 2.748 | 2.072 | -1.066 | -1.046 |
| TDG | 2.747 | 1.378 | 1.056 | -1.055 |
| FOSL1 | 2.745 | 1.152 | 1.042 | -1.029 |
| LGALS3 | 2.734 | 2.68 | 1.147 | 1.068 |
| KIF9 | 2.734 | 2.946 | 1.247 | -1.128 |
| NT5C3A | 2.733 | -3.548 | 1.613 | 1.342 |
| CCND3 | 2.73 | 2.378 | 1.425 | 1.043 |
| UACA | 2.73 | 1.027 | 1.122 | -1.034 |
| CDK18 | 2.727 | 1.041 | 1.182 | 1.004 |
| XKRX | 2.725 | 6.494 | -1.928 | -1.127 |
| GBP2 | 2.706 | 3.502 | 1.683 | -1.237 |
| ALOX5AP | 2.69 | 2.156 | 1.68 | 1.489 |
| DAP | 2.689 | 1.118 | 1.724 | -1.172 |
| DACH1 | 2.689 | 2.516 | 1.117 | -1.207 |
| LIN28A | 2.684 | 2.25 | -1.063 | 1.059 |
| CNNM2 | 2.678 | 2.877 | 1.157 | 1.167 |
| PFKL | 2.668 | 3.411 | 3.515 | 1.049 |
| Gm20604 | 2.643 | 3.013 | -1.601 | 1.522 |
| TMEM216 | 2.636 | 1.585 | 1.157 | 1.156 |
| TLR4 | 2.616 | 1.395 | 1.214 | -1.059 |
| LIMS1 | 2.607 | 1.586 | -1.347 | 1.007 |
| VILL | 2.602 | 2.089 | 1.792 | 1.126 |
| F2RL2 | 2.599 | 1.451 | 1.727 | 1.085 |
| ITGA2B | 2.593 | -2.239 | -2.924 | 2.94 |
| GPLD1 | 2.59 | 1.959 | 2.238 | 1.275 |
| PAM | 2.588 | 3.447 | 1.871 | 1.425 |
| CMAS | 2.586 | -3.053 | 1.695 | -1.151 |
| NQO1 | 2.586 | 1.519 | 1.061 | -1.328 |
| RFC2 | 2.573 | 1.941 | -1.027 | -1.066 |
| TAGLN2 | 2.571 | 2.602 | 2.419 | 1.131 |
| ACADL | 2.569 | 3.646 | 1.294 | 1.079 |
| TRAK2 | 2.555 | -1.161 | 1.555 | 1.169 |
| PDE2A | 2.552 | 1.96 | -1.19 | 1.159 |
| Cmah | 2.541 | 2.005 | 2.343 | -1.817 |
| PPP1R16B | 2.53 | 1.388 | 2.239 | -1.697 |
| PGM1 | 2.529 | 3.55 | 1.732 | 1.004 |
| LRRK2 | 2.523 | 2.186 | -1.426 | -1.238 |
| NFE2 | 2.511 | 1.448 | 1.445 | -1.121 |
| RUFY4 | 2.506 | 2.804 | 4.203 | 1.055 |
| SAMD9L | 2.499 | 2.142 | 1.21 | -1.871 |
| PLP2 | 2.497 | 1.598 | 1.149 | -1.339 |
| PNKP | 2.488 | 1.981 | -1.054 | 1.139 |
| Gm10865 | 2.482 | 1.538 | -1.354 | -1.024 |
| FLOT1 | 2.477 | 2.004 | 1.507 | 1.092 |
| Gm4013 | 2.475 | -2.814 | 1.602 | 1.504 |
| BYSL | 2.473 | 2.14 | 1.403 | 1.186 |
| AZIN1 | 2.471 | -1.326 | 1.325 | -1.041 |
| GPR55 | 2.449 | 1.449 | 1.929 | 1.124 |
| SELP | 2.442 | 2.156 | 2.717 | 2.584 |
| SLC28A2 | 2.44 | 2.484 | 1.47 | 3.493 |
| RAB24 | 2.437 | 2.224 | 1.746 | 1.124 |
| Nrgn | 2.433 | -2.929 | -1.23 | 1.101 |
| INTS12 | 2.419 | 2.04 | 1.049 | -1.068 |
| IRGM | 2.418 | 1.37 | 1.897 | -1.211 |
| RASA2 | 2.409 | -1.153 | -1.12 | -1.158 |
| CXCR1 | 2.408 | 2.792 | 5.112 | 1.656 |
| C4orf19 | 2.408 | 3.746 | 4.395 | 1.066 |
| LASP1 | 2.407 | 1.771 | 1.519 | -1.438 |
| CAPG | 2.403 | 1.719 | -1.067 | -1.117 |
| C16orf54 | 2.399 | 1.249 | 1.265 | -1.219 |
| Gm12070 | 2.395 | 1.611 | 1.811 | -1.105 |
| ARHGDIB | 2.394 | 1.611 | 1.146 | -1.288 |
| PPM1M | 2.383 | 1.713 | -1.203 | -1.18 |
| DUSP6 | 2.377 | 1.085 | 2.132 | -2.872 |
| RSC1A1 | 2.365 | 2.515 | 1.069 | -1.079 |
| SNAPC5 | 2.357 | 1.995 | 1.286 | -1.414 |
| SLC25A45 | 2.356 | 1.942 | 1.394 | -1.44 |
| PGAM1 | 2.351 | 2.307 | 1.312 | 1.654 |
| PTGIR | 2.346 | 1.423 | -1.17 | 2.665 |
| IL27 | 2.344 | 1.926 | 1.231 | -2.158 |
| GPI | 2.343 | 1.965 | 1.106 | 1.33 |
| CCDC125 | 2.343 | 2.251 | -1.329 | -1.023 |
| ABCA13 | 2.342 | 1.692 | -1.01 | 1.012 |
| XAF1 | 2.34 | 2.166 | 1.254 | -2.402 |
| NSMCE1 | 2.339 | 2.13 | 2.089 | -1.197 |
| PGD | 2.335 | 1.708 | 1.156 | 1.219 |
| THAP4 | 2.328 | 1.635 | 1.089 | 1.016 |
| EID1 | 2.328 | 2.63 | 1.432 | -1.2 |
| FKBPL | 2.317 | 1.91 | 1.344 | 1.319 |
| RHOC | 2.315 | 2.045 | 1.219 | -1.239 |
| ASNS | 2.313 | 1.529 | 1.107 | -1.065 |
| 9830107B12Rik/A530064D06Rik | 2.312 | 1.601 | -1.341 | 1.103 |
| LARS2 | 2.312 | -3.155 | 1.123 | -1.136 |
| MPST | 2.307 | 1.381 | -2.03 | -1.843 |
| AK2 | 2.305 | 2.401 | 1.201 | 1.213 |
| ID1 | 2.304 | 2.372 | 1.718 | 7.657 |
| PABPC1L | 2.297 | 1.587 | 1.174 | -1.012 |
| MVP | 2.295 | 2.532 | 2.412 | -1.081 |
| ALDH2 | 2.294 | 1.217 | -1.192 | -1.342 |
| APOBR | 2.281 | 1.715 | 1.149 | -1.344 |
| FABP5 | 2.28 | 3.582 | 1.773 | 1.607 |
| SMPDL3A | 2.28 | 4.143 | 1.31 | -1.475 |
| TRIOBP | 2.275 | 1.515 | -1.023 | -1.462 |
| IFITM3 | 2.271 | 2.029 | 1.78 | -1.096 |
| SNX1 | 2.271 | 2.838 | 1.85 | -1.239 |
| ENTPD3 | 2.267 | 2.297 | 4.998 | -1.114 |
| ALAS1 | 2.262 | 2.288 | 2.441 | 1.491 |
| KLF7 | 2.262 | 2.041 | 1.731 | 1.027 |
| LY75 | 2.259 | 1.688 | -1.076 | 1.258 |
| Stfa1 (includes others) | 2.259 | 1.274 | 2.057 | 1.227 |
| CYBB | 2.245 | 2.694 | 1.005 | 2.179 |
| CSF2RB | 2.244 | 3.021 | 4.649 | 4.916 |
| RTP4 | 2.24 | 1.513 | 1.514 | -4.901 |
| DGKG | 2.238 | 1.99 | 1.219 | -1.792 |
| GALNS | 2.234 | 2.134 | -1.115 | 1.212 |
| PRAM1 | 2.233 | 1.901 | 2.798 | 1.306 |
| CCDC85B | 2.232 | 1.353 | 1.415 | -1.409 |
| SUB1 | 2.231 | 2.861 | 1.412 | -1.227 |
| MBD2 | 2.22 | 2.188 | 1.28 | 1.093 |
| CYB5R4 | 2.216 | 1.862 | -1.107 | 1.007 |
| Oasl2 | 2.215 | 2.14 | 2.639 | -3.014 |
| CTNNBIP1 | 2.213 | 1.796 | 1.231 | -1.068 |
| CBR3 | 2.21 | 3.502 | 2.126 | 1.187 |
| SP140 | 2.21 | 2.133 | 1.015 | -1.325 |
| LXN | 2.209 | 2.832 | 1.453 | -1.235 |
| HILPDA | 2.201 | 1.011 | 1.223 | -1.499 |
| USP46 | 2.197 | 1.383 | -1.215 | -1.11 |
| NTPCR | 2.196 | 2.374 | 1.182 | -1.019 |
| OAS2 | 2.194 | 1.803 | 2.753 | -2.02 |
| ETHE1 | 2.193 | 1.667 | -1.348 | 1.232 |
| APPBP2 | 2.189 | 1.821 | 1.063 | -1.151 |
| Tmem18 | 2.186 | 2.986 | 1.297 | 1.152 |
| ICA1 | 2.184 | 1.273 | -1.146 | -1.245 |
| TNFAIP8L2 | 2.181 | 1.577 | 1.34 | 1.073 |
| CDKN2D | 2.177 | 1.806 | 1.491 | 1.057 |
| MGST1 | 2.174 | 1.609 | -1.349 | -1.08 |
| ELOVL7 | 2.173 | 1.41 | -1.267 | -1.036 |
| CASP4 | 2.171 | 2.806 | 2.533 | 1.349 |
| F630028O10Rik | 2.168 | -1.268 | -1.075 | 1.453 |
| MPPE1 | 2.167 | 2.105 | -1.293 | -1.219 |
| E030030I06Rik | 2.167 | -1.114 | -1.129 | -1.56 |
| PYGL | 2.165 | 1.927 | 1.158 | -1.081 |
| HRH2 | 2.163 | 2.539 | 3.11 | 1.402 |
| Saa3 | 2.161 | 1.537 | 2.297 | 1.834 |
| Ifi27l2a/Ifi27l2b | 2.161 | 1.841 | 1.625 | -10.005 |
| KCTD10 | 2.155 | 2.193 | -1.066 | -1.191 |
| FAM162A | 2.15 | 1.481 | 2.737 | 1.052 |
| NXN | 2.147 | 1.327 | -1.026 | 1.079 |
| USP18 | 2.146 | 1.41 | 1.181 | -2.977 |
| SPINT1 | 2.144 | 6.376 | 1.932 | 1.943 |
| Slfn1 | 2.144 | 1.55 | 1.476 | 1.059 |
| ALOX12 | 2.142 | -1.987 | -1.895 | -1.086 |
| PRKAB1 | 2.14 | 1.602 | 1.206 | -1.05 |
| ALDOA | 2.139 | 2.053 | 1.471 | 1.075 |
| MEFV | 2.135 | 1.564 | 3.706 | 1.499 |
| BEND4 | 2.134 | 1.711 | 1.628 | -1.278 |
| HLA-A | 2.132 | 2.929 | 4.19 | -1.258 |
| TPI1 | 2.131 | 2.712 | 2.028 | -1.039 |
| SIPA1L1 | 2.126 | 2.416 | 2.071 | 2.016 |
| DNMT3L | 2.125 | 1.71 | 1.253 | -1.151 |
| TUBB1 | 2.122 | -2.651 | -3.019 | -1.181 |
| Fus | 2.115 | 1.094 | 1.84 | 1.151 |
| C16orf72 | 2.113 | 1.113 | 1.419 | 1.208 |
| CTSV | 2.108 | 2.223 | 1.347 | 1.483 |
| NCBP1 | 2.104 | 1.392 | 1.154 | 1.258 |
| A330040F15Rik | 2.104 | -1.137 | -1.067 | 1.108 |
| JAK3 | 2.101 | 2.447 | 4.073 | 1.085 |
| TMOD3 | 2.1 | 2.049 | 1.664 | 1.172 |
| B430306N03Rik | 2.096 | 1.737 | 1.64 | -1.034 |
| H2-K2/H2-Q9 | 2.094 | 2.398 | 2.487 | 1.157 |
| TIRAP | 2.093 | 1.368 | 1.279 | 1.122 |
| SREBF2 | 2.091 | 2.336 | 1.625 | -1.418 |
| RND1 | 2.09 | 3.732 | 2.324 | -1.271 |
| CPD | 2.083 | 1.94 | 1.393 | 1.06 |
| E2F2 | 2.078 | -3.178 | 1.509 | 1.098 |
| SEMA4A | 2.077 | 2.346 | 2.528 | 2.86 |
| A730008H23Rik | 2.076 | 1.43 | 1.284 | -1.005 |
| FFAR2 | 2.073 | 2.129 | -1.291 | 3.738 |
| DENND11 | 2.073 | 1.37 | 1.049 | 1.126 |
| CCNB2 | 2.072 | -3.782 | -1.107 | 1.116 |
| CTNS | 2.071 | 2.389 | 1.527 | 1.16 |
| CDK2 | 2.071 | 1.523 | -1.103 | -1.119 |
| RFX2 | 2.064 | 2.963 | 1.599 | 1.019 |
| LIME1 | 2.062 | 1.221 | -1.079 | 1.182 |
| MED10 | 2.06 | 2.005 | 1.094 | -1.338 |
| ACOX3 | 2.053 | 1.713 | 1.372 | 1.2 |
| FUNDC1 | 2.051 | 2.975 | 1.342 | 1.006 |
| UBE2H | 2.049 | -1.258 | 1.65 | -1.195 |
| ZNF414 | 2.048 | 1.789 | 2.225 | 1.063 |
| S100A13 | 2.043 | 1.015 | 1.065 | -1.349 |
| 2310010J17Rik | 2.041 | -1.521 | 1.06 | 1.874 |
| IL18R1 | 2.04 | 2.114 | 1.363 | 2.937 |
| GCHFR | 2.037 | -6.578 | 1.138 | 1.371 |
| STX11 | 2.035 | 1.229 | -1.276 | 1.696 |
| MOGAT2 | 2.035 | 1.072 | -1.138 | 1.05 |
| PDCD6 | 2.034 | 1.455 | -1.21 | 1.059 |
| SLA2 | 2.033 | 1.665 | -1.115 | -1.13 |
| CLIC1 | 2.031 | 1.669 | 1.41 | -1.097 |
| TRIM56 | 2.031 | 1.566 | -1.237 | -1.137 |
| TPCN2 | 2.03 | 1.707 | 1.389 | -1.014 |
| POLK | 2.028 | 1.483 | 1.218 | 1.116 |
| BST1 | 2.026 | 1.541 | 1.468 | 1.046 |
| PFKFB2 | 2.022 | 1.572 | 2.217 | -1.008 |
| SLC37A3 | 2.022 | 1.736 | 1.31 | -1.276 |
| MTA3 | 2.021 | 1.576 | 2.385 | -1.519 |
| PRTN3 | 2.019 | 2.093 | 1.133 | 1.879 |
| TMED8 | 2.019 | 1.783 | 1.195 | 1.134 |
| STRADB | 2.019 | -3.485 | 1.496 | -1.071 |
| RPS6KA2 | 2.018 | 1.584 | -1.006 | 1.342 |
| KLF5 | 2.018 | 2.361 | 2.046 | -1.203 |
| LPP | 2.016 | 2.169 | 1.772 | -1.233 |
| PROM1 | 2.014 | 1.535 | -1.184 | 1.374 |
| DDX28 | 2.012 | 2.609 | 3.035 | -1.179 |
| NCF4 | 2.01 | 1.917 | 1.353 | 1.074 |
| ARMC7 | 2.01 | 1.303 | 1.576 | -1.174 |
| SMOX | 2.01 | -1.686 | 2.206 | -1.361 |
| RAC2 | 2.007 | 1.542 | 1.263 | -1.146 |
| AACS | 2.005 | 1.615 | 1.194 | 1.258 |
| CAPZA1 | 2.005 | 1.876 | 1.129 | 1.21 |
| ZNHIT1 | 2.004 | 2.047 | 1.341 | 1.344 |
| AGL | 2.001 | 1.746 | -1.141 | 1.205 |
| EIF4EBP1 | 1.999 | 1.904 | 1.73 | 1.036 |
| MGAM | 1.996 | 1.49 | -1.022 | -1.018 |
| OSTF1 | 1.993 | 2.016 | 1.148 | 1.136 |
| ACVR1B | 1.993 | 2.369 | 1.616 | -1.051 |
| SLC25A14 | 1.993 | 1.531 | 1.182 | -1.121 |
| FIS1 | 1.992 | -1.625 | 1.046 | -1.087 |
| PPP1R3B | 1.992 | 1.359 | 1.235 | -1.152 |
| ADORA3 | 1.99 | 2.198 | 2.773 | 1.468 |
| TBC1D8 | 1.99 | 1.312 | 1.023 | -1.274 |
| ACAD11 | 1.989 | 2.21 | -1.38 | -1.068 |
| Prss34 | 1.985 | 1.463 | -6.587 | -1.329 |
| RNF34 | 1.984 | 1.414 | 1.102 | 1.03 |
| OPTN | 1.984 | -1.426 | 1.005 | -1.243 |
| ERO1A | 1.983 | 1.74 | 1.474 | 1.478 |
| GPAA1 | 1.981 | 1.728 | 1.438 | 1.485 |
| IL18RAP | 1.981 | 1.599 | -1.004 | 1.386 |
| ANKRA2 | 1.98 | 1.202 | -1.214 | 1.135 |
| IFIT1B | 1.98 | 1.588 | 1.304 | -4.324 |
| MSRA | 1.978 | 1.661 | -1.04 | -1.027 |
| AP3S1 | 1.977 | 1.426 | 1.574 | 3.192 |
| SP110 | 1.976 | 1.581 | 1.065 | -1.133 |
| PRSS57 | 1.975 | 2.189 | 1.159 | 2.005 |
| F10 | 1.975 | 1.858 | 2.422 | 1.316 |
| TRAF1 | 1.973 | 1.916 | 3.521 | -1.294 |
| HIF1A | 1.973 | 1.982 | 2.503 | -1.341 |
| SNAI1 | 1.972 | 7.326 | 5.604 | 1.07 |
| SLC35D2 | 1.972 | 2.249 | 1.16 | -1.365 |
| NT5E | 1.97 | 1.789 | -1.377 | 1.603 |
| GPR84 | 1.969 | 3.367 | 2.135 | 1.281 |
| MMP9 | 1.963 | 1.728 | 1.217 | 1.492 |
| CD300LF | 1.963 | 1.579 | 1.984 | 1.409 |
| HP | 1.958 | 1.45 | 1.213 | 10.552 |
| Irgm1 | 1.957 | 1.526 | 1.224 | -1.536 |
| SLC7A5 | 1.955 | -10.402 | 2.905 | 1.383 |
| VRK3 | 1.954 | 1.674 | 1.64 | -1.013 |
| ZNF207 | 1.954 | 1.645 | 1.205 | -1.112 |
| KIAA0513 | 1.952 | 1.767 | 1.151 | 1.095 |
| Rpph1 | 1.946 | -1.322 | -1.452 | -1.101 |
| GP9 | 1.945 | -1.404 | -1.712 | 1.256 |
| ANXA7 | 1.943 | 2.219 | 1.482 | 1.095 |
| FBXL5 | 1.943 | 1.638 | 1.908 | -1.107 |
| CDC14A | 1.943 | 2.154 | 1.024 | -1.169 |
| HMGCR | 1.943 | 1.074 | 1.001 | -1.295 |
| KIAA1109 | 1.942 | 1.143 | -1.09 | 1.006 |
| SCHIP1 | 1.942 | 1.241 | 1.575 | -1.063 |
| DEGS1 | 1.937 | 1.527 | -1.285 | 1.067 |
| PADI4 | 1.935 | 1.566 | 1.029 | 1.001 |
| DPCD | 1.935 | 2.018 | 1.507 | -1.418 |
| NHSL2 | 1.934 | 1.581 | 1.191 | 1.555 |
| DBNL | 1.934 | 1.884 | 1.221 | 1.061 |
| RAB28 | 1.93 | 1.795 | -1.003 | 1.049 |
| Hmgn2 (includes others) | 1.93 | 1.481 | 1.054 | -1.291 |
| SSBP4 | 1.929 | 2.446 | 1.544 | 1.401 |
| TRIP4 | 1.928 | 1.616 | -1.091 | -1.24 |
| CARHSP1 | 1.928 | -1.108 | 1.289 | -1.535 |
| KLHL6 | 1.927 | 1.227 | -1.285 | 1.114 |
| CELA1 | 1.927 | -1.04 | -5.889 | -1.087 |
| P2RY1 | 1.925 | 1.661 | 1.562 | 1.455 |
| TPM3 | 1.922 | 1.627 | 1.2 | -1.053 |
| PEF1 | 1.921 | 1.66 | -1.112 | 1.066 |
| RNF25 | 1.921 | 1.896 | 1.037 | -1.111 |
| CCDC88B | 1.921 | 1.649 | 1.01 | -1.149 |
| EBI3 | 1.921 | 1.874 | 1.324 | -1.509 |
| MVK | 1.911 | 1.398 | 1.088 | -1.275 |
| APH1B | 1.91 | 1.377 | -1.366 | -1.032 |
| RIPK3 | 1.907 | 3.307 | -1.008 | -1.041 |
| GRN | 1.906 | 2.435 | 1.317 | 1.214 |
| CD200R1 | 1.906 | 1.819 | 1.468 | 1.198 |
| SLC31A2 | 1.905 | 2.152 | -1.002 | -1.198 |
| TADA3 | 1.905 | 1.346 | 1.052 | -1.228 |
| CELSR3 | 1.904 | 1.293 | 1.332 | 1.007 |
| 6430706D22Rik | 1.904 | 2.008 | -1.049 | -1.322 |
| Oas1b | 1.904 | 1.498 | 1.513 | -1.798 |
| ECI2 | 1.903 | 2.677 | 1.244 | 1.031 |
| GBP7 | 1.902 | 1.813 | 1.074 | -1.343 |
| ADAM15 | 1.9 | 1.84 | 2.037 | 1.449 |
| CEBPE | 1.898 | 1.079 | -1.41 | 1.217 |
| C6orf120 | 1.897 | 1.744 | 1.305 | 1.104 |
| Igtp | 1.897 | 1.588 | 1.193 | 1.072 |
| CAPNS1 | 1.896 | 1.483 | 1.434 | -1.142 |
| AP2S1 | 1.895 | 1.656 | 1.143 | 1.037 |
| CMTM6 | 1.895 | 1.47 | 1.16 | -1.044 |
| GALE | 1.892 | 1.556 | 1.243 | 1.219 |
| NDUFV3 | 1.892 | 1.524 | 1.122 | 1.004 |
| Pcmt1 | 1.892 | -1.53 | -1.086 | -1.123 |
| SH3BGRL3 | 1.891 | 1.614 | 1.243 | -1.063 |
| KLHL28 | 1.889 | 1.205 | -1.007 | 1.019 |
| SH3BP5 | 1.889 | 1.268 | -1.486 | -1.545 |
| TIMP2 | 1.888 | 1.878 | 1.11 | -1.541 |
| ITGAM | 1.887 | 1.577 | 1.219 | 1.826 |
| TWF2 | 1.886 | 1.725 | -1.057 | -1.013 |
| ARPC4 | 1.885 | 1.327 | 1.194 | -1.035 |
| MSMP | 1.884 | -1.415 | 1.726 | 1.002 |
| Gm10012 | 1.883 | -2.855 | -1.223 | 1.032 |
| DTNB | 1.882 | 1.247 | 1.165 | 1.172 |
| IRF9 | 1.88 | 1.408 | 1.113 | -1.826 |
| DMXL2 | 1.878 | 2.039 | 1.382 | 1.086 |
| D2HGDH | 1.876 | 1.773 | 1.206 | -1.329 |
| XDH | 1.874 | 1.372 | -1.193 | 2.275 |
| YKT6 | 1.874 | 1.507 | 1.426 | 1.249 |
| ZNF76 | 1.868 | 1.165 | 1.221 | 1.022 |
| ACPP | 1.867 | 1.314 | -1.013 | -1.013 |
| GDA | 1.866 | -1.363 | -1.045 | 1.898 |
| TRDMT1 | 1.866 | 2.163 | 1.281 | 1.344 |
| TES | 1.866 | 1.661 | -1.019 | -1.016 |
| MSRB2 | 1.866 | 1.252 | -1.348 | -1.302 |
| RIOK2 | 1.865 | 1.228 | 1.403 | -1.11 |
| FLYWCH2 | 1.863 | -1.325 | 1.285 | -1.198 |
| EML2 | 1.862 | 1.402 | 1.652 | 1.022 |
| ZFC3H1 | 1.861 | 1.189 | 1.155 | 1.067 |
| FLNA | 1.861 | 1.585 | -1.011 | 1.044 |
| AOAH | 1.86 | -1.478 | -1.379 | 1.09 |
| MRPL45 | 1.859 | 1.337 | -1.275 | 1.068 |
| CYB561D1 | 1.858 | 1.304 | 1.017 | 1.058 |
| CYTH4 | 1.858 | 1.401 | 1.521 | -1.205 |
| CCNC | 1.856 | 1.791 | 1.14 | 1.046 |
| KHNYN | 1.855 | 1.315 | 1.221 | -1.363 |
| Cd33 | 1.855 | 1.476 | 1.859 | -1.551 |
| ISY1-RAB43 | 1.852 | 1.282 | 1.144 | 1.03 |
| LBR | 1.851 | 1.501 | 1.129 | 1.112 |
| ANKRD28 | 1.849 | 1.211 | -1.318 | 1.51 |
| PLOD3 | 1.848 | 1.843 | 1.082 | 1.691 |
| ACOT8 | 1.846 | 1.646 | 1.175 | -1.08 |
| MAFG | 1.846 | 1.491 | -1.212 | -1.182 |
| HK1 | 1.84 | 1.461 | 1.534 | 1.291 |
| ACTN1 | 1.84 | 1.526 | 1.223 | -1.329 |
| NFU1 | 1.839 | 2.191 | -1.021 | -1.042 |
| CTSE | 1.838 | 1.037 | -1.268 | 1.352 |
| RAB23 | 1.838 | 2.571 | 1.254 | -1.186 |
| C20orf27 | 1.836 | -2.435 | 1.295 | -1.259 |
| C8orf44-SGK3/SGK3 | 1.834 | 2.108 | 1.395 | 1.332 |
| EGLN3 | 1.832 | 3.968 | 3.997 | 1.363 |
| CASP1 | 1.83 | 2.075 | 1.132 | 1.06 |
| RNASEL | 1.83 | 1.349 | 1.272 | -1.128 |
| SEMA6B | 1.828 | 2.673 | 5.877 | 3.897 |
| GTPBP1 | 1.827 | 1.508 | 1.13 | 1.137 |
| POLD4 | 1.827 | 1.378 | 1.205 | -1.02 |
| GLIPR2 | 1.827 | 1.687 | 1.3 | -1.211 |
| TCP11L1 | 1.826 | 2.424 | 1.729 | 1.27 |
| MAPK3 | 1.825 | 1.477 | 1.087 | -1.126 |
| CSTF3 | 1.824 | 1.082 | -1.046 | -1.007 |
| CENPB | 1.823 | 1.702 | 1.965 | 1.267 |
| MRPS18C | 1.822 | 1.899 | 1.455 | -1.082 |
| NT5C2 | 1.817 | 1.742 | 1.113 | -1.106 |
| TRAF3IP3 | 1.817 | -1.014 | -1.041 | -1.338 |
| Gm12250 | 1.817 | 1.724 | 1.137 | -1.351 |
| NMRK1 | 1.817 | 1.751 | 1.496 | -1.62 |
| UBA7 | 1.815 | 1.547 | 1.123 | 1.053 |
| RECK | 1.815 | 2.105 | 1.504 | -1.107 |
| MEGF9 | 1.815 | 1.452 | 1.213 | -1.244 |
| C15orf39 | 1.815 | 1.3 | 1.998 | -1.364 |
| STX18 | 1.814 | 1.939 | 1.031 | 1.059 |
| PFN1 | 1.812 | 1.575 | 2.416 | -1.079 |
| ATXN1 | 1.812 | 1.627 | 1.09 | -1.12 |
| PPP6R1 | 1.811 | 1.371 | -1.025 | -1.093 |
| MTUS1 | 1.81 | 1.847 | 1.172 | 1.232 |
| PTPN7 | 1.807 | 2.056 | 1.203 | -1.059 |
| YWHAB | 1.805 | 1.74 | -1.001 | -1.115 |
| EIF2AK2 | 1.804 | 1.591 | 1.01 | -1.358 |
| PARP10 | 1.802 | 1.597 | 1.437 | -1.281 |
| INPP4A | 1.802 | 1.378 | -1.011 | -1.391 |
| Slfn2 | 1.801 | 1.464 | 1.337 | 3.267 |
| 1810037I17Rik/Gm2036 | 1.801 | 1.603 | 1.179 | -1.214 |
| HSD17B11 | 1.801 | 1.752 | 1.072 | -1.499 |
| CASZ1 | 1.8 | 1.45 | 1.293 | -1.067 |
| OGFR | 1.8 | 1.49 | 1.006 | -1.151 |
| OAS3 | 1.798 | 1.347 | 1.51 | -2.66 |
| UCK2 | 1.797 | 1.115 | 1.286 | 1.307 |
| RAB37 | 1.795 | 1.477 | 1.048 | -1.124 |
| OSGIN1 | 1.795 | 1.419 | 2.304 | -2.916 |
| CNN2 | 1.794 | 1.452 | 1.028 | -1.055 |
| RNF6 | 1.793 | 1.34 | 1.01 | -1.05 |
| PDK1 | 1.792 | 2.11 | 2.273 | 1.15 |
| MAX | 1.791 | 1.308 | 1.247 | 1.13 |
| CDS2 | 1.79 | 1.799 | 1.307 | 1.061 |
| RAB31 | 1.787 | 1.915 | 1.712 | 1.102 |
| Cd52 | 1.787 | 1.385 | 1.436 | -1.085 |
| CAMKK1 | 1.786 | 1.377 | 2.67 | 1.036 |
| B230216N24Rik | 1.785 | 3.236 | 1.948 | -1.033 |
| ARHGAP30 | 1.784 | 1.376 | 1.012 | -1.217 |
| GRINA | 1.782 | 1.568 | 1.823 | 1.082 |
| C19orf38 | 1.781 | 1.695 | 1.133 | 1.812 |
| Serpina3g (includes others) | 1.78 | -1.447 | -6.607 | 4.457 |
| TTC13 | 1.78 | 1.811 | -1.129 | 1.438 |
| DDX6 | 1.78 | 1.679 | 1.431 | 1.086 |
| ARHGAP19 | 1.779 | 1.261 | -1.775 | 1.358 |
| AMPD3 | 1.779 | 1.837 | 2.06 | -1.478 |
| RHOU | 1.778 | 1.718 | 1.405 | -1.07 |
| CUEDC2 | 1.777 | 1.567 | 1.068 | 1.121 |
| 1810026B05Rik | 1.777 | 1.211 | 1.168 | -1.456 |
| LARP4B | 1.776 | 1.366 | 1.156 | 1.023 |
| RUSC1 | 1.775 | 1.802 | 1.849 | 1.308 |
| CLASP1 | 1.775 | 1.277 | -1.056 | 1.117 |
| ITGB1BP1 | 1.775 | 1.573 | 1.31 | -1.07 |
| SOAT1 | 1.774 | 1.968 | 1.168 | 2.071 |
| RPE | 1.774 | 1.418 | -1.092 | -1.079 |
| PILRB | 1.774 | 1.174 | 1.067 | -1.265 |
| P4HA1 | 1.773 | 3.859 | 2.781 | -1.379 |
| UBE2C | 1.771 | -78.691 | -1.467 | 1.025 |
| HNRNPA2B1 | 1.769 | 1.381 | 1.142 | 1.193 |
| MED20 | 1.769 | 1.028 | 1.014 | -1.409 |
| PPP1R15B | 1.767 | 1.733 | 1.626 | 1.058 |
| TOM1 | 1.767 | 1.807 | -1.03 | -1.082 |
| NUDT22 | 1.765 | 1.783 | 1.461 | -1.179 |
| VCL | 1.762 | 1.277 | 1.068 | 1.104 |
| TRIM21 | 1.762 | 1.672 | 1.093 | -1.335 |
| CD14 | 1.76 | 2.133 | 4.76 | 1.899 |
| 6330407A03Rik | 1.757 | 1.455 | -1.305 | -1.079 |
| Sp100 | 1.757 | 1.338 | 1.305 | -1.424 |
| BCL2L11 | 1.756 | 1.363 | 1.894 | 1.185 |
| LILRB3 | 1.755 | 1.61 | 2.393 | 1.146 |
| CDK5R1 | 1.754 | 1.239 | -1.315 | -1.045 |
| KBTBD7 | 1.752 | 1.572 | 1.318 | -1.008 |
| RTN3 | 1.748 | 1.349 | 1.519 | 1.156 |
| GCNT1 | 1.748 | 1.883 | 1.986 | 1.128 |
| CD37 | 1.748 | 1.617 | 1.467 | -1.037 |
| IL4R | 1.747 | 2.39 | 3.599 | 1.536 |
| STAMBPL1 | 1.747 | 1.455 | 1.201 | 1.203 |
| LCP1 | 1.747 | 1.63 | 1.679 | 1.048 |
| ATP6V1G1 | 1.747 | 1.405 | 1.162 | -1.016 |
| HCFC1R1 | 1.745 | 1.54 | 1.178 | -1.45 |
| PLD1 | 1.744 | 1.97 | 1.086 | -1.009 |
| SDF2 | 1.744 | 1.325 | -1.079 | -1.054 |
| RAB8B | 1.743 | 1.795 | 1.283 | 1.114 |
| HINT3 | 1.743 | -1.232 | 1.195 | -1.177 |
| GABARAPL2 | 1.743 | -10.62 | -1.044 | -1.18 |
| CDK5 | 1.742 | 1.645 | -1.111 | 1.032 |
| MXD3 | 1.741 | 2.492 | 1.477 | -1.292 |
| PGM2 | 1.739 | 2.024 | 1.986 | 1.161 |
| ARHGAP1 | 1.739 | 1.47 | 1.678 | 1.116 |
| CCDC12 | 1.739 | 1.259 | -1.018 | -1.182 |
| TRAPPC3 | 1.738 | 1.231 | -1.036 | -1.011 |
| CFLAR | 1.738 | 1.223 | -1.005 | -1.034 |
| ATP13A2 | 1.737 | 2.243 | 1.093 | -1.044 |
| H2-T9 | 1.736 | 2.273 | -1.228 | 1.098 |
| KATNB1 | 1.736 | 1.54 | 1.348 | -1.006 |
| ANXA2 | 1.734 | 1.613 | 1.232 | 1.654 |
| DDI2 | 1.734 | 2.254 | 3.775 | 1.498 |
| SOAT2 | 1.732 | 2.068 | 4.547 | 1.192 |
| Evi5l | 1.732 | 1.171 | -1.043 | -1.084 |
| ATP2C1 | 1.731 | 1.436 | 1.001 | 1.145 |
| OSBPL9 | 1.73 | 1.907 | 1.261 | 1.002 |
| NDUFA8 | 1.73 | 1.936 | 1.176 | -1.155 |
| GALNT3 | 1.728 | 1.647 | -1.624 | 1.019 |
| TP53INP1 | 1.728 | 1.897 | -1.054 | -1.025 |
| ERG | 1.728 | 1.046 | 1.036 | -1.204 |
| TESK2 | 1.726 | 1.092 | 1.122 | 1.141 |
| Dgcr6 | 1.724 | 1.129 | 1.048 | 1.213 |
| ARHGAP24 | 1.724 | 1.535 | 1.387 | -1.113 |
| ADAM19 | 1.722 | 1.708 | 1.645 | -1.018 |
| COPZ1 | 1.721 | 1.417 | 1.58 | 1.045 |
| IL12A | 1.72 | 1.338 | 1.058 | -3.178 |
| ARPC5 | 1.717 | 1.458 | 1.038 | -1.017 |
| HMGB2 | 1.717 | 1.668 | 1.209 | -1.068 |
| SNX18 | 1.716 | 1.345 | 1.063 | 1.152 |
| CYFIP1 | 1.716 | 2.631 | 1.276 | 1.053 |
| SERTAD3 | 1.715 | 1.284 | -1.013 | -1.618 |
| ATP11B | 1.714 | 1.393 | 1.049 | -1.009 |
| GCC1 | 1.714 | 1.054 | 1.221 | -1.277 |
| KRTCAP3 | 1.713 | 1.518 | 1.233 | 2.185 |
| WDR1 | 1.712 | 1.296 | -1.043 | -1.026 |
| NCF1 | 1.711 | 1.518 | -1.029 | -1.172 |
| PNPLA8 | 1.709 | -1.106 | -1.226 | -1.045 |
| 3110056K07Rik | 1.708 | 1.691 | 1.042 | -1.678 |
| NDUFB7 | 1.707 | 1.401 | 1.038 | -1.023 |
| MLST8 | 1.707 | 1.067 | 1.046 | -1.036 |
| FAM114A2 | 1.706 | 1.238 | 1.132 | -1.31 |
| GGH | 1.705 | 2.139 | -1.163 | -1.021 |
| MYL6 | 1.704 | -1.343 | -1.025 | -1.101 |
| CPNE3 | 1.703 | 1.362 | -1.147 | 1.074 |
| RINL | 1.702 | 1.601 | 1.033 | 1.064 |
| UBASH3A | 1.701 | -1.181 | -1.38 | 1.445 |
| CDKN2AIP | 1.701 | 1.467 | 1.145 | -1.044 |
| GPX1 | 1.7 | -2.992 | 1.223 | -1.005 |
| RNPEP | 1.697 | 1.764 | 1.202 | 1.015 |
| GNAI3 | 1.697 | 1.515 | 1.01 | -1.005 |
| CCDC28A | 1.697 | 1.442 | -1.125 | -1.025 |
| PRKCB | 1.697 | 1.648 | -1.003 | -1.263 |
| PFDN1 | 1.697 | 3.486 | 1.104 | -1.279 |
| VPS4B | 1.696 | 1.488 | 1.074 | -1.063 |
| PSMB9 | 1.696 | 1.886 | 1.039 | -1.122 |
| LAMTOR2 | 1.694 | 1.589 | -1.081 | -1.141 |
| RHOBTB2 | 1.693 | 1.335 | 1.577 | -1.07 |
| DCAF12 | 1.691 | -18.395 | -1.137 | -1.044 |
| SLC40A1 | 1.691 | 1.556 | -1.284 | -2.107 |
| ATP8A1 | 1.69 | 1.744 | -1.142 | 1.164 |
| 6530402F18Rik | 1.689 | 1.156 | 1.136 | -1.08 |
| RASSF5 | 1.688 | 1.812 | 1.267 | -1.119 |
| SLC16A3 | 1.687 | 1.173 | 1.294 | 1.319 |
| LAMP2 | 1.685 | 1.676 | 1.622 | 1.55 |
| COMTD1 | 1.685 | 1.562 | -1.175 | 1.289 |
| SDR39U1 | 1.685 | 1.46 | 1.041 | 1.265 |
| ST3GAL5 | 1.685 | -2.157 | -1.395 | -1.452 |
| Tnfrsf26 | 1.683 | -1.051 | 1.208 | 1.254 |
| CRTAC1 | 1.683 | 1.369 | -1.561 | 1.121 |
| IKBKE | 1.683 | 1.654 | 1.231 | 1.114 |
| CSNK2B | 1.682 | 1.32 | 1.036 | 1.088 |
| FBXO28 | 1.68 | 1.248 | 1.094 | -1.005 |
| BCS1L | 1.679 | 2.617 | -1.173 | -1.09 |
| MMADHC | 1.678 | 2.037 | 1.293 | 1.02 |
| MRPL34 | 1.678 | 1.342 | 1.128 | -1.021 |
| ANKRD16 | 1.677 | 1.174 | 1.141 | 1.213 |
| ACTR3 | 1.675 | 1.634 | 1.014 | 1.221 |
| FCER1G | 1.674 | 1.533 | 1.051 | 1.071 |
| CTSD | 1.673 | 1.538 | 1.293 | 1.403 |
| JMJD6 | 1.672 | 1.285 | -1.088 | 1.076 |
| CDKN3 | 1.671 | 1.143 | -1.269 | -1.134 |
| MITD1 | 1.671 | 1.024 | 1.028 | -1.186 |
| PELI2 | 1.671 | 1.767 | 1.062 | -1.355 |
| ELOVL1 | 1.67 | 1.807 | 1.201 | 1.142 |
| TMEM42 | 1.67 | 1.496 | 1.485 | -1.003 |
| PINK1 | 1.669 | -1.846 | 1.33 | 1.001 |
| BIN3 | 1.669 | 1.775 | 1.319 | -1.023 |
| PTGS1 | 1.669 | -1.069 | -1.351 | -1.386 |
| RNF20 | 1.668 | -1.871 | -1.224 | -1.096 |
| FERMT3 | 1.667 | 1.342 | 1.034 | 1.079 |
| POLR2H | 1.667 | 1.735 | 1.69 | -1.115 |
| GSTCD | 1.667 | -1.106 | 1.048 | -1.163 |
| NPEPPS | 1.667 | 1.354 | 1.292 | -1.24 |
| Cd24a | 1.666 | -1.659 | -1.086 | 1.131 |
| NRAS | 1.663 | 1.041 | 1.53 | 1.068 |
| PDZD11 | 1.663 | 1.881 | 1.054 | -1.068 |
| CLEC5A | 1.66 | 1.267 | 1.131 | 1.562 |
| AHSA1 | 1.66 | 1.322 | 1.072 | 1.173 |
| PPP1R16A | 1.66 | 2.192 | 1.694 | -1.024 |
| KCTD20 | 1.659 | 1.254 | -1.039 | -1.05 |
| STXBP2 | 1.658 | 1.472 | 1.197 | 1.156 |
| FDPS | 1.658 | 2.212 | 1.892 | -1.402 |
| CLEC4A | 1.657 | 1.361 | -1.226 | 1.222 |
| PRDX6 | 1.657 | -1.024 | -1.091 | -1.068 |
| PTGER2 | 1.656 | 1.118 | 1.377 | -1.766 |
| PNP | 1.654 | 2.417 | 1.634 | 1.388 |
| PIWIL2 | 1.654 | 1.75 | 2.968 | 1.267 |
| SPRYD3 | 1.653 | 1.688 | 2.765 | 1.219 |
| COMMD1 | 1.653 | 1.008 | -1.035 | 1.07 |
| Gt(ROSA)26Sor | 1.653 | 1.086 | 1.061 | -1.238 |
| TREX1 | 1.652 | 2.066 | 1.871 | -1.142 |
| ATP6V1D | 1.652 | 1.637 | 1.136 | -1.3 |
| TGFBI | 1.651 | 1.867 | 1.97 | 1.652 |
| NLRX1 | 1.651 | 1.958 | 1.455 | 1.534 |
| CDC42EP4 | 1.651 | 1.031 | 1.086 | 1.211 |
| APOBEC3B | 1.65 | 1.803 | 3.472 | 1.335 |
| IFITM2 | 1.649 | 1.573 | 3.426 | 1.121 |
| ARPC3 | 1.649 | 1.852 | 1.244 | 1.092 |
| ADAM8 | 1.649 | 1.16 | 1.272 | 1.087 |
| PRKAG1 | 1.649 | 1.447 | 1.056 | 1.073 |
| TRIM5 | 1.649 | 1.449 | 1.072 | -1.586 |
| UBA1 | 1.648 | 1.307 | 1.082 | 1.036 |
| CRISPLD2 | 1.648 | 1.315 | -1.107 | -1.039 |
| Gm10408 (includes others) | 1.647 | -1.254 | 1.321 | 1.278 |
| STIM2 | 1.647 | 1.165 | -1.025 | 1.26 |
| SLC27A4 | 1.647 | 1.095 | -1.126 | 1.078 |
| PLIN3 | 1.647 | 1.853 | 1.355 | -1.113 |
| ADK | 1.646 | 2.011 | -2.005 | 1.156 |
| HECA | 1.646 | 1.417 | -1.076 | 1.07 |
| VAMP8 | 1.644 | 1.495 | 1.106 | 1.145 |
| SLC25A44 | 1.644 | 1.814 | 1.673 | 1.076 |
| TRAPPC1 | 1.644 | 1.803 | 1.17 | 1.004 |
| MOSPD1 | 1.643 | 1.264 | 1.036 | 1.41 |
| NKG7 | 1.643 | -2.033 | -1.168 | 1.266 |
| TRAPPC4 | 1.643 | 1.29 | 1.166 | -1.108 |
| POMP | 1.643 | 1.634 | -1.197 | -1.186 |
| BBS9 | 1.64 | 1.48 | 1.045 | 1.267 |
| 2610001J05Rik | 1.64 | 1.596 | 1.217 | 1.248 |
| FAM111A | 1.639 | 1.212 | -1.218 | 1.024 |
| KIN | 1.638 | 1.467 | 1.007 | -1.209 |
| 1500011B03Rik | 1.637 | -1.069 | 1.295 | 1.005 |
| TANK | 1.636 | 1.287 | 1.013 | 1.492 |
| MSL1 | 1.634 | 1.67 | 1.088 | -1.456 |
| KISS1R | 1.632 | 1.118 | -1.055 | 1.017 |
| ADAR | 1.632 | 1.385 | 1.531 | -1.518 |
| PXN | 1.631 | 1.448 | 2.205 | -1.087 |
| ENSA | 1.63 | 1.503 | 1.349 | -1.116 |
| PDLIM2 | 1.63 | 2.038 | 2.357 | -1.441 |
| C19orf25 | 1.629 | 1.773 | 1.281 | -1.075 |
| CORO7/CORO7-PAM16 | 1.629 | 1.39 | 1.483 | -1.143 |
| AP1S3 | 1.628 | 1.258 | 1.178 | -1.162 |
| PDCL3 | 1.627 | 1.736 | 1.261 | -1.075 |
| Cdc42 | 1.627 | 1.532 | 1.263 | -1.103 |
| SPHK2 | 1.625 | 1.552 | 1.689 | -1.068 |
| RNF10 | 1.624 | -15.071 | 1.275 | 1.093 |
| B3GNT2 | 1.623 | 1.047 | -1.055 | 1.065 |
| Mff | 1.623 | -1.454 | -1.001 | -1.123 |
| ING4 | 1.622 | 1.316 | 1.019 | -1.206 |
| Gdap10 | 1.622 | 1.005 | 1.19 | -1.512 |
| Raet1d/Raet1e | 1.621 | -1.243 | -1.233 | -1.052 |
| MRPS21 | 1.62 | 1.287 | 1.181 | -1.123 |
| MAP2K4 | 1.618 | 1.382 | 1.337 | 1.216 |
| HSD17B7 | 1.618 | 1.11 | -1.147 | 1.033 |
| CDK19 | 1.618 | 1.434 | 1.331 | -1.302 |
| MDH2 | 1.617 | 1.474 | 1.163 | 1.2 |
| MAP3K11 | 1.617 | 1.52 | 1.956 | 1.028 |
| DTX4 | 1.616 | 2.275 | 3.012 | 1.733 |
| Rn45s | 1.616 | -4.741 | -1.32 | -1.075 |
| MVD | 1.615 | 1.755 | 1.432 | 1.283 |
| PCMTD2 | 1.615 | 1.514 | -1.1 | -1.22 |
| ARID3A | 1.614 | 1.505 | 1.107 | -1.004 |
| RHBDF2 | 1.613 | 1.245 | 1.843 | 1.396 |
| CAPN10 | 1.613 | 1.539 | 1.303 | 1.102 |
| HDAC5 | 1.613 | 1.436 | 1.969 | 1.067 |
| GDAP2 | 1.613 | 1.499 | 1.169 | -1.07 |
| WDR37 | 1.612 | 1.382 | 1.073 | 1.084 |
| BNIP3 | 1.612 | 1.795 | 2.515 | -1.133 |
| SNAP29 | 1.612 | 1.383 | 1.846 | -1.136 |
| UBL5 | 1.611 | 1.27 | 1.251 | -1.113 |
| Esrra | 1.61 | 1.659 | 1.308 | 1.068 |
| SCAPER | 1.61 | 1.372 | -1.198 | -1.455 |
| SNF8 | 1.609 | 1.159 | -1.004 | 1.084 |
| 3110021A11Rik | 1.608 | 1.262 | -1.098 | 1.38 |
| BMX | 1.608 | 1.534 | -1.243 | 1.362 |
| SLC9A6 | 1.608 | 2.061 | 1.423 | 1.253 |
| HPCAL1 | 1.608 | 1.559 | 1.241 | -1.229 |
| CFL1 | 1.607 | 1.301 | 1.494 | 1.059 |
| ALDH3B1 | 1.606 | 1.569 | 1.076 | 1.258 |
| RNF181 | 1.606 | 1.44 | 1.231 | -1.011 |
| HSD17B10 | 1.606 | 1.698 | 1.038 | -1.171 |
| GHDC | 1.606 | 1.271 | 1.008 | -1.28 |
| IL13RA1 | 1.605 | 1.967 | 3.848 | 1.438 |
| 9330175E14Rik | 1.605 | 1.08 | 1.135 | 1.058 |
| CORO1A | 1.605 | 1.481 | 1.144 | -1.137 |
| PPP1R3D | 1.605 | 1.651 | 2.061 | -1.197 |
| MPDU1 | 1.604 | 1.405 | 1.267 | 1.126 |
| GMFG | 1.604 | 1.306 | 1.117 | -1.245 |
| AIFM2 | 1.604 | 2.58 | -1.043 | -1.327 |
| SOS2 | 1.603 | 1.432 | 1.976 | 1.002 |
| TBRG1 | 1.603 | 1.728 | 1.042 | -1.186 |
| RNF144A | 1.603 | 1.17 | 1.177 | -1.41 |
| DHRS9 | 1.602 | 1.132 | 1.027 | 1.074 |
| KRI1 | 1.602 | 1.556 | 1.339 | 1.073 |
| ORMDL1 | 1.602 | 1.432 | 1.163 | -1.281 |
| ATXN7L3B | 1.602 | 1.496 | -1.038 | -1.426 |
| RAB6A | 1.601 | 1.272 | -1.086 | -1.103 |
| SFT2D1 | 1.601 | 1.405 | -1.058 | -1.203 |
| PRSS16 | 1.6 | 1.334 | 1.07 | 1.518 |
| 4930594C11Rik | 1.599 | -1.712 | -1.123 | 1.103 |
| TTLL1 | 1.598 | 1.36 | 1.046 | 1.115 |
| ISG20 | 1.598 | -12.507 | 1.119 | -1.82 |
| NR1H2 | 1.597 | 1.39 | 1.496 | -1.166 |
| GPR137 | 1.596 | 1.187 | -1.042 | -1.09 |
| HCK | 1.594 | 1.622 | 1.321 | 1.632 |
| DCTN3 | 1.594 | 1.588 | 1.137 | -1.093 |
| ATG9A | 1.594 | 1.3 | 1.478 | -1.167 |
| CNR2 | 1.591 | 1.065 | 1.18 | 1.272 |
| NADK | 1.591 | 1.663 | 1.041 | 1.263 |
| MICU1 | 1.591 | 1.694 | 1.083 | 1.041 |
| TFE3 | 1.59 | 1.277 | 1.465 | 1.15 |
| TMEM189 | 1.59 | 1.251 | 1.437 | 1.133 |
| XXYLT1 | 1.589 | 4.036 | 1.747 | 1.059 |
| ZFYVE19 | 1.589 | 2.217 | 1.432 | -1.084 |
| ACADSB | 1.588 | 1.164 | 1.098 | -1.002 |
| IL16 | 1.586 | 1.086 | -1.016 | -1.388 |
| SBSN | 1.585 | 1.759 | 1.106 | 1.49 |
| C3 | 1.584 | 1.324 | -1.178 | 2.783 |
| IL2RG | 1.584 | 2.116 | 3.181 | 1.485 |
| CHMP1A | 1.584 | 1.339 | 1.31 | 1.091 |
| GNG12 | 1.584 | 1.609 | 1.306 | -1.084 |
| SNX27 | 1.584 | 1.541 | -1.122 | -1.633 |
| IRF7 | 1.584 | -1.058 | 1.615 | -3.715 |
| GNAI2 | 1.583 | 1.468 | 1.422 | -1.027 |
| DHDDS | 1.582 | 1.216 | 1.252 | -1.155 |
| SLFN13 | 1.581 | 1.17 | 1.193 | -2.058 |
| SLC25A11 | 1.58 | 1.581 | -1.007 | -1.062 |
| PIP5K1B | 1.58 | 1.871 | 1.357 | -1.441 |
| TP53INP2 | 1.577 | 1.234 | 3.229 | -1.183 |
| STXBP5 | 1.576 | 1.141 | 1.12 | 1.187 |
| GPANK1 | 1.576 | 1.198 | 1.034 | 1.113 |
| TMEM101 | 1.576 | 1.637 | -1.106 | 1.093 |
| GLOD4 | 1.576 | 1.757 | 1.224 | -1.069 |
| KHK | 1.575 | 1.302 | -1.114 | 1.206 |
| ZBTB42 | 1.575 | 1.427 | 1.61 | 1.076 |
| PYCARD | 1.575 | 1.823 | 1.307 | 1.054 |
| ITGA5 | 1.574 | 2.093 | 1.777 | 1.298 |
| NAMPT | 1.574 | 1.965 | -1.126 | 1.011 |
| NIPSNAP3A | 1.573 | 1.61 | 1.52 | 1.117 |
| CBY1 | 1.573 | 1.662 | 1.111 | -1.017 |
| SHISA5 | 1.573 | 1.381 | 2.504 | -1.268 |
| C6orf89 | 1.572 | 1.37 | 1.141 | 1.17 |
| TAF1B | 1.572 | 2.017 | 1.423 | 1.109 |
| OSBPL2 | 1.572 | 1.68 | -1.038 | 1.032 |
| RGS18 | 1.572 | -1.241 | -1.257 | -1.213 |
| PI16 | 1.571 | 1.407 | 1.208 | 3.032 |
| CDC27 | 1.571 | 1.087 | -1.155 | 1.214 |
| DDX60 | 1.57 | 1.515 | -1.23 | -1.57 |
| TMEM50B | 1.569 | 1.689 | 1.153 | 1.831 |
| TNFAIP6 | 1.569 | -1.878 | -1.287 | 1.055 |
| ALDH3A2 | 1.569 | 1.549 | -1.063 | -1.267 |
| FKBP8 | 1.568 | 1.294 | 1.049 | 1.035 |
| ATP6V0A1 | 1.568 | 1.672 | 1.577 | -1.109 |
| GGT5 | 1.568 | 1.51 | 1.506 | -1.138 |
| NUMB | 1.567 | 1.363 | 1.271 | -1.03 |
| CD84 | 1.567 | 1.669 | -1.14 | -1.084 |
| HCST | 1.566 | -1.015 | -1.07 | 1.271 |
| PSMD13 | 1.565 | 1.567 | 1.177 | 1.136 |
| Cxcl3 | 1.564 | 1.536 | 3.033 | 1.12 |
| B4GALT5 | 1.564 | 1.237 | 2.005 | 1.031 |
| RNF5 | 1.564 | 1.172 | 1.013 | -1.297 |
| HLTF | 1.564 | 1.302 | 1.042 | -1.31 |
| IL1RAP | 1.564 | 1.815 | 2.787 | -1.378 |
| SRF | 1.563 | 1.041 | 1.151 | -1.075 |
| Gm15645 | 1.562 | 1.138 | -1.171 | 1.087 |
| ABHD5 | 1.562 | 1.606 | -1.228 | -1.152 |
| CHMP2B | 1.561 | 1.827 | 1.085 | -1.018 |
| GPD2 | 1.561 | 1.628 | 1.086 | -1.092 |
| CDC37L1 | 1.56 | 1.183 | -1.009 | 1.036 |
| TCF25 | 1.56 | 1.235 | 1.137 | -1.057 |
| RIT1 | 1.56 | 1.437 | -1.01 | -1.329 |
| PER2 | 1.56 | 1.842 | 1.019 | -1.474 |
| BCKDHA | 1.559 | 1.654 | 1.22 | 1.086 |
| NDUFB6 | 1.559 | -1.272 | -1.131 | 1.061 |
| ANXA11 | 1.559 | 1.86 | 1.891 | -1.067 |
| TCP11L2 | 1.559 | 1.447 | 1.151 | -1.067 |
| PLEC | 1.559 | 1.477 | 1.17 | -1.111 |
| USB1 | 1.559 | 1.617 | 1.485 | -1.171 |
| TMCO3 | 1.559 | 1.974 | 1.254 | -1.19 |
| OSBPL11 | 1.558 | 1.344 | -1.035 | -1.031 |
| 2610507I01Rik | 1.557 | 1.486 | 1.039 | 1.102 |
| STK38 | 1.557 | 1.785 | 1.167 | 1.002 |
| PARP12 | 1.557 | 1.945 | 1.222 | -1.145 |
| AGGF1 | 1.557 | 1.454 | -1.016 | -1.17 |
| RDH12 | 1.557 | 1.382 | -1.078 | -1.918 |
| Gm16062 | 1.556 | -1.525 | 1.334 | 1.4 |
| ACRBP | 1.556 | 1.391 | 1.311 | 1.065 |
| RBM5 | 1.556 | 1.083 | -1.067 | -1.597 |
| NIF3L1 | 1.555 | 1.245 | 1.21 | 1.218 |
| CDC20 | 1.555 | -1.236 | -1.132 | 1.093 |
| HIPK1 | 1.555 | -1.655 | 1.086 | -1.009 |
| PRKAA1 | 1.555 | 1.489 | 1.35 | -1.039 |
| TAP1 | 1.555 | 1.669 | 1.291 | -1.045 |
| LEO1 | 1.555 | 1.36 | 1.407 | -1.061 |
| OASL | 1.555 | 1.543 | 1.439 | -2.088 |
| ANKRD27 | 1.553 | 1.323 | 1.004 | 1.491 |
| DMKN | 1.551 | 1.551 | 1.555 | 2.546 |
| ADD3 | 1.551 | 1.596 | -1.09 | 1.139 |
| SLC6A4 | 1.551 | 1.718 | -1.358 | 1.038 |
| PARP14 | 1.551 | 1.29 | 1.224 | -1.069 |
| MED7 | 1.551 | 1.532 | 1.941 | -1.305 |
| VTA1 | 1.55 | 1.382 | 1.094 | -1.048 |
| Acad12 | 1.55 | 1.201 | 1.191 | -1.207 |
| CYP51A1 | 1.55 | 1.327 | 1.156 | -1.837 |
| MTMR6 | 1.549 | 1.623 | 1.031 | -1.015 |
| TBC1D14 | 1.549 | 1.394 | 1.807 | -1.092 |
| TAP2 | 1.549 | 1.313 | 1.142 | -1.112 |
| Gm12854/S100a11 | 1.549 | 1.18 | 1.259 | -1.118 |
| VASP | 1.549 | 1.596 | 1.276 | -1.198 |
| IRAK3 | 1.548 | 1.535 | 1.631 | 1.131 |
| DCUN1D3 | 1.548 | 1.273 | -1.032 | -1.031 |
| Nos1ap | 1.548 | 1.475 | 1.425 | -1.134 |
| NDFIP1 | 1.548 | 1.594 | 1.051 | -1.192 |
| POP7 | 1.547 | 1.851 | 1.147 | -1.009 |
| TMEM9B | 1.546 | 1.589 | 1.204 | 1.256 |
| TRNT1 | 1.546 | 1.316 | 1.086 | 1.151 |
| SPC24 | 1.546 | -1.54 | -1.19 | -1.015 |
| PDCD10 | 1.545 | 1.609 | 1.186 | 1.011 |
| DCAF11 | 1.545 | 1.088 | -1.097 | -1.054 |
| LGALS9B | 1.545 | 1.763 | 1.731 | -1.172 |
| TRIB3 | 1.545 | 1.372 | 1.652 | -1.654 |
| SLC25A20 | 1.544 | 1.186 | 1.358 | 1.313 |
| APRT | 1.544 | 1.57 | 1.01 | 1.071 |
| TSTA3 | 1.543 | 1.713 | 1.098 | 1.106 |
| TRAPPC2 | 1.543 | -1.01 | -1.068 | -1.086 |
| IFI35 | 1.543 | 1.454 | 1.238 | -1.55 |
| CYFIP2 | 1.542 | 1.56 | 1.08 | 1.035 |
| ACTB | 1.542 | 1.397 | 1.186 | -1.107 |
| IPCEF1 | 1.542 | 1.368 | 1.262 | -1.621 |
| DHX58 | 1.542 | 1.623 | -1.079 | -2.617 |
| ARL5B | 1.541 | -1.006 | 1.202 | -1.068 |
| PHF23 | 1.541 | 1.438 | 1.325 | -1.189 |
| CLN5 | 1.54 | 1.803 | 1.332 | 1.096 |
| AP3B1 | 1.54 | 1.449 | 1.013 | -1.041 |
| MYD88 | 1.539 | 1.699 | 1.82 | 1.464 |
| Gm15417 | 1.539 | 1.121 | -1.227 | 1.382 |
| SCP2 | 1.538 | 1.584 | -1.043 | -1.025 |
| UNC13D | 1.537 | 1.266 | -1.004 | 1.094 |
| UBE2L6 | 1.537 | -157.312 | -1.02 | -1.864 |
| GAPT | 1.537 | 1.296 | 1.438 | -2.125 |
| YWHAG | 1.536 | 1.482 | 1.399 | 1.097 |
| CDK11A | 1.535 | 1.133 | -1.475 | -1.078 |
| RPS6KA1 | 1.535 | 1.433 | 1.005 | -1.106 |
| 2310015A10Rik | 1.535 | 1.636 | 1.969 | -1.132 |
| 5033406O09Rik | 1.535 | -1.325 | -1.269 | -1.194 |
| MAPK7 | 1.534 | 1.28 | -1.115 | 1.085 |
| COMMD3 | 1.534 | 1.538 | 1.019 | -1.056 |
| CYBA | 1.533 | 1.388 | 1.096 | -1.131 |
| CPNE2 | 1.529 | 2.019 | 1.723 | 1.149 |
| BECN1 | 1.529 | -2.121 | 1.085 | 1.118 |
| CUTA | 1.529 | 1.236 | 1.188 | -1.011 |
| ZMAT2 | 1.529 | 1.077 | 1.07 | -1.064 |
| H2AZ1 | 1.528 | 1.356 | -1.234 | 1.113 |
| PSMF1 | 1.528 | -2.703 | -1.093 | -1.128 |
| ATRN | 1.527 | 1.016 | -1.148 | 1.216 |
| MED22 | 1.527 | 1.925 | 1.148 | 1.046 |
| FAM110A | 1.527 | 1.262 | 1.644 | -1.254 |
| SH3YL1 | 1.526 | -2.051 | 1.061 | 1.153 |
| ATG4B | 1.526 | 1.124 | 1.133 | -1.049 |
| TBC1D20 | 1.526 | 1.304 | 1.198 | -1.17 |
| EMILIN2 | 1.525 | 1.67 | 2.757 | 8.32 |
| CEP164 | 1.525 | 1.69 | -1.182 | -1.165 |
| Bud31 | 1.524 | 1.16 | 1.061 | -1.064 |
| CHMP2A | 1.524 | 1.411 | 1.202 | -1.118 |
| TAF12 | 1.524 | 1.483 | 1.184 | -1.173 |
| ARHGAP9 | 1.523 | 1.533 | 1.151 | 1.174 |
| KCTD11 | 1.523 | 1.483 | 1.799 | 1.147 |
| MAEA | 1.523 | 1.392 | 1.056 | 1.011 |
| STIM1 | 1.523 | 1.483 | -1.121 | -1.037 |
| ZFAND2B | 1.522 | 1.466 | 1.044 | 1.029 |
| XRCC4 | 1.522 | 1.441 | 1.052 | -1.555 |
| DHRS7 | 1.521 | 1.213 | -1.264 | 1.302 |
| NDUFS6 | 1.521 | -1.372 | -1.115 | 1.089 |
| LRRC27 | 1.521 | 1.68 | 1.589 | 1.041 |
| DMAP1 | 1.521 | 1.512 | 1.012 | -1.157 |
| SEC61B | 1.52 | 1.182 | 1.054 | 1.056 |
| SCNM1 | 1.52 | 1.267 | 1.029 | -1.328 |
| COTL1 | 1.52 | 1.438 | 1.536 | -1.539 |
| RSU1 | 1.519 | 1.556 | 1.046 | 1.168 |
| WBP1 | 1.519 | 1.113 | -1.104 | 1.014 |
| Trim30a/Trim30d | 1.519 | 1.821 | 1.851 | -1.522 |
| BLOC1S1 | 1.518 | 1.301 | 1.017 | -1.147 |
| MAGOH | 1.517 | 1.037 | -1.082 | 1.042 |
| A930019D19Rik | 1.517 | 1.059 | 1.074 | -1.147 |
| SKAP2 | 1.516 | 1.462 | 1.326 | 1.095 |
| COG6 | 1.516 | 1.648 | 1.025 | 1.014 |
| CHAC2 | 1.516 | -2.957 | -1.41 | -1.023 |
| AKT2 | 1.515 | 1.306 | 1.353 | 1.053 |
| ANTKMT | 1.515 | 1.1 | 1.035 | 1.002 |
| SHOC2 | 1.515 | 1.306 | 2.084 | -1.121 |
| Fam169b | 1.515 | -1.745 | 1.574 | -1.322 |
| PARP3 | 1.514 | 1.236 | 1.116 | 1.22 |
| ABTB1 | 1.514 | 1.437 | 1.005 | 1.014 |
| GPBP1L1 | 1.514 | 1.096 | 1.368 | -1.207 |
| TMEM33 | 1.513 | 1.189 | 1.102 | 1.126 |
| PPP1R18 | 1.513 | 1.262 | 2.688 | -1.247 |
| CETN2 | 1.512 | -1.227 | -1.094 | -1.196 |
| LCOR | 1.512 | 1.175 | 1.03 | -1.251 |
| RNF114 | 1.512 | 1.152 | 1.146 | -1.371 |
| C14orf119 | 1.511 | 1.85 | -1.072 | 1.223 |
| ANKRD13A | 1.511 | 1.44 | 1.213 | -1.034 |
| USP36 | 1.509 | -1.437 | -1.045 | -1.115 |
| 5830432E09Rik | 1.509 | 1.098 | -1.207 | -1.146 |
| ECH1 | 1.508 | 1.315 | -1.371 | 1.059 |
| STK16 | 1.508 | 1.217 | -1.074 | 1.051 |
| PIGX | 1.508 | 1.519 | 1.169 | -1.042 |
| ARF5 | 1.508 | -3.861 | 1.143 | -1.047 |
| CDC42EP2 | 1.508 | 1.038 | -1.374 | -1.09 |
| ZFYVE27 | 1.508 | 1.275 | -1.036 | -1.104 |
| H3-3A/H3-3B | 1.508 | 1.185 | 1.207 | -1.217 |
| EIF4G2 | 1.507 | -1.155 | 1.251 | 1.042 |
| ALDOC | 1.507 | 2.795 | 2.208 | -2.832 |
| VMP1 | 1.506 | 1.236 | 1.871 | 1.272 |
| ENTPD5 | 1.506 | 1.462 | 1.223 | 1.211 |
| PTEN | 1.506 | 1.565 | 1.152 | -1.136 |
| EXOC1 | 1.505 | 1.579 | -1.007 | -1.006 |
| ATXN7L3 | 1.505 | 1.074 | 1.267 | -1.128 |
| PAIP2 | 1.505 | -1.013 | 1.151 | -1.292 |
| Gvin1 (includes others) | 1.505 | 1.661 | 1.128 | -1.354 |
| NDUFAF1 | 1.504 | 1.032 | -1.087 | -1.003 |
| VHL | 1.504 | 1.513 | 1.023 | -1.132 |
| Apol11b (includes others) | 1.503 | -43.162 | -1.092 | 1.992 |
| B4GALNT1 | 1.503 | 2.416 | 3.747 | 1.602 |
| ME2 | 1.503 | 1.424 | -1.2 | 1.34 |
| TMEM160 | 1.503 | 1.399 | 1.009 | 1.159 |
| DTX3L | 1.503 | 1.753 | 1.209 | -1.27 |
| VSIG10 | 1.503 | 1.861 | 1.455 | -1.341 |
| ITGB2 | 1.502 | 1.389 | 1.085 | -1.234 |
| RAP2C | 1.501 | 1.455 | 1.186 | 1.108 |
| GRK6 | 1.501 | 1.537 | 1.01 | 1.081 |
| MPP1 | 1.501 | -1.894 | 1.392 | -1.224 |

**Gene sets enriched in the Ly6G^+^ neutrophils in the JAK2^V617F^ lung compared to those in the WT lung from RNA sequence.**

| Name | Size | ES | NES | NOM p-val | FDR q-val |
| --- | --- | --- | --- | --- | --- |
| REACTOME_NEUTROPHIL_DEGRANULATION | 439 | 0.57 | 2.37 | 0.000 | 0.000 |
| REACTOME_CHOLESTEROL_BIOSYNTHESIS | 25 | 0.73 | 2.02 | 0.000 | 0.013 |
| REACTOME_RHO_GTPASES_ACTIVATE_NADPH_OXIDASES | 24 | 0.74 | 1.97 | 0.000 | 0.022 |
| REACTOME_RHO_GTPASES_ACTIVATE_WASPS_AND_WAVES | 36 | 0.66 | 1.94 | 0.002 | 0.025 |
| BIOCARTA_RAS_PATHWAY | 22 | 0.73 | 1.92 | 0.000 | 0.030 |
| KEGG_NICOTINATE_AND_NICOTINAMIDE_METABOLISM | 23 | 0.72 | 1.91 | 0.000 | 0.031 |
| REACTOME_AUTOPHAGY | 101 | 0.53 | 1.89 | 0.000 | 0.036 |
| SIG_INSULIN_RECEPTOR_PATHWAY_IN_CARDIAC_MYOCYTES | 50 | 0.60 | 1.89 | 0.000 | 0.034 |
| REACTOME_REGULATION_OF_MITOTIC_CELL_CYCLE | 80 | 0.55 | 1.89 | 0.000 | 0.031 |
| KEGG_PENTOSE_PHOSPHATE_PATHWAY | 25 | 0.67 | 1.86 | 0.000 | 0.040 |
| REACTOME_SIGNALING_BY_NTRK1_TRKA | 75 | 0.55 | 1.86 | 0.002 | 0.038 |
| REACTOME_PROGRAMMED_CELL_DEATH | 166 | 0.49 | 1.86 | 0.000 | 0.036 |
| REACTOME_APC_C:CDH1_MEDIATED_DEGRADATION_OF_CDC20_AND_OTHER_APC_C:CDH1_TARGETED_PROTEINS_IN_LATE_MITOSIS_EARLY_G1 | 66 | 0.56 | 1.84 | 0.000 | 0.041 |
| PID_IL8_CXCR2_PATHWAY | 33 | 0.63 | 1.84 | 0.000 | 0.041 |
| KEGG_PROTEASOME | 42 | 0.60 | 1.83 | 0.000 | 0.040 |
| REACTOME_MACROAUTOPHAGY | 84 | 0.54 | 1.83 | 0.000 | 0.041 |
| REACTOME_CDK_MEDIATED_PHOSPHORYLATION_AND_REMOVAL_OF_CDC6 | 66 | 0.55 | 1.82 | 0.000 | 0.042 |
| PID_RAC1_PATHWAY | 54 | 0.57 | 1.81 | 0.000 | 0.042 |
| REACTOME_ACTIVATION_OF_APC_C_AND_APC_C:CDC20_MEDIATED_DEGRADATION_OF_MITOTIC_PROTEINS | 70 | 0.55 | 1.81 | 0.000 | 0.042 |
| PID_TOLL_ENDOGENOUS_PATHWAY | 21 | 0.69 | 1.81 | 0.000 | 0.041 |
| BIOCARTA_IGF1R_PATHWAY | 22 | 0.67 | 1.80 | 0.005 | 0.045 |
| REACTOME_REGULATION_OF_PTEN_STABILITY_AND_ACTIVITY | 64 | 0.55 | 1.80 | 0.000 | 0.043 |
| REACTOME_DDX58_IFIH1_MEDIATED_INDUCTION_OF_INTERFERON_ALPHA_BETA | 66 | 0.53 | 1.80 | 0.002 | 0.044 |
| REACTOME_REGULATION_OF_ACTIN_DYNAMICS_FOR_PHAGOCYTIC_CUP_FORMATION | 59 | 0.54 | 1.80 | 0.000 | 0.042 |
| KEGG_SNARE_INTERACTIONS_IN_VESICULAR_TRANSPORT | 34 | 0.62 | 1.79 | 0.004 | 0.046 |
| REACTOME_SIGNALLING_TO_ERKS | 31 | 0.63 | 1.78 | 0.000 | 0.046 |
| REACTOME_VIF_MEDIATED_DEGRADATION_OF_APOBEC3G | 49 | 0.57 | 1.78 | 0.002 | 0.048 |
| REACTOME_ENDOSOMAL_SORTING_COMPLEX_REQUIRED_FOR_TRANSPORT_ESCRT | 28 | 0.65 | 1.77 | 0.007 | 0.051 |
| REACTOME_METABOLISM_OF_POLYAMINES | 56 | 0.55 | 1.76 | 0.000 | 0.054 |
| KEGG_GLYCOLYSIS_GLUCONEOGENESIS | 56 | 0.55 | 1.76 | 0.000 | 0.054 |
| REACTOME_RAB_REGULATION_OF_TRAFFICKING | 117 | 0.49 | 1.76 | 0.000 | 0.052 |
| REACTOME_DEGRADATION_OF_DVL | 53 | 0.55 | 1.75 | 0.000 | 0.054 |
| PID_IL8_CXCR1_PATHWAY | 27 | 0.64 | 1.75 | 0.002 | 0.056 |
| REACTOME_INTERFERON_ALPHA_BETA_SIGNALING | 54 | 0.55 | 1.74 | 0.000 | 0.059 |
| REACTOME_REGULATION_OF_APOPTOSIS | 49 | 0.55 | 1.74 | 0.000 | 0.059 |
| REACTOME_DEFECTIVE_CFTR_CAUSES_CYSTIC_FIBROSIS | 57 | 0.55 | 1.74 | 0.000 | 0.060 |
| REACTOME_FBXL7_DOWN_REGULATES_AURKA_DURING_MITOTIC_ENTRY_AND_IN_EARLY_MITOSIS | 51 | 0.55 | 1.73 | 0.000 | 0.062 |
| KEGG_STARCH_AND_SUCROSE_METABOLISM | 34 | 0.58 | 1.72 | 0.002 | 0.073 |
| REACTOME_REGULATION_OF_TLR_BY_ENDOGENOUS_LIGAND | 17 | 0.69 | 1.72 | 0.000 | 0.071 |
| REACTOME_CREB1_PHOSPHORYLATION_THROUGH_NMDA_RECEPTOR_MEDIATED_ACTIVATION_OF_RAS_SIGNALING | 27 | 0.62 | 1.71 | 0.009 | 0.073 |
| REACTOME_ABC_TRANSPORTER_DISORDERS | 73 | 0.50 | 1.71 | 0.000 | 0.075 |
| REACTOME_PLATELET_HOMEOSTASIS | 85 | 0.49 | 1.70 | 0.000 | 0.076 |
| KEGG_REGULATION_OF_AUTOPHAGY | 30 | 0.60 | 1.70 | 0.006 | 0.076 |
| REACTOME_SIGNAL_AMPLIFICATION | 31 | 0.59 | 1.70 | 0.002 | 0.077 |
| REACTOME_ROS_AND_RNS_PRODUCTION_IN_PHAGOCYTES | 36 | 0.59 | 1.70 | 0.007 | 0.077 |
| REACTOME_MICROAUTOPHAGY | 25 | 0.63 | 1.69 | 0.006 | 0.078 |
| REACTOME_RHO_GTPASE_EFFECTORS | 279 | 0.42 | 1.69 | 0.000 | 0.077 |
| REACTOME_TBC_RABGAPS | 43 | 0.56 | 1.69 | 0.002 | 0.078 |
| REACTOME_BUDDING_AND_MATURATION_OF_HIV_VIRION | 23 | 0.63 | 1.69 | 0.010 | 0.078 |
| REACTOME_APOPTOTIC_CLEAVAGE_OF_CELLULAR_PROTEINS | 38 | 0.57 | 1.69 | 0.007 | 0.078 |
| REACTOME_NEGATIVE_REGULATION_OF_NOTCH4_SIGNALING | 49 | 0.54 | 1.69 | 0.002 | 0.077 |
| REACTOME_DEGRADATION_OF_AXIN | 51 | 0.54 | 1.68 | 0.006 | 0.076 |
| REACTOME_TRANSLOCATION_OF_SLC2A4_GLUT4_TO_THE_PLASMA_MEMBRANE | 66 | 0.51 | 1.68 | 0.000 | 0.076 |
| REACTOME_DEGRADATION_OF_GLI1_BY_THE_PROTEASOME | 55 | 0.53 | 1.68 | 0.004 | 0.075 |
| REACTOME_ENERGY_DEPENDENT_REGULATION_OF_MTOR_BY_LKB1_AMPK | 28 | 0.61 | 1.67 | 0.009 | 0.087 |
| REACTOME_TP53_REGULATES_METABOLIC_GENES | 79 | 0.50 | 1.67 | 0.000 | 0.086 |
| REACTOME_NUCLEOBASE_CATABOLISM | 35 | 0.57 | 1.66 | 0.004 | 0.086 |
| BIOCARTA_MPR_PATHWAY | 20 | 0.65 | 1.66 | 0.010 | 0.086 |
| REACTOME_ZBP1_DAI_MEDIATED_INDUCTION_OF_TYPE_I_IFNS | 21 | 0.63 | 1.66 | 0.006 | 0.086 |
| KEGG_FC_GAMMA_R_MEDIATED_PHAGOCYTOSIS | 92 | 0.47 | 1.66 | 0.000 | 0.086 |
| BIOCARTA_LIS1_PATHWAY | 18 | 0.67 | 1.66 | 0.002 | 0.085 |
| BIOCARTA_EIF4_PATHWAY | 24 | 0.60 | 1.66 | 0.014 | 0.085 |
| REACTOME_MAP2K_AND_MAPK_ACTIVATION | 39 | 0.55 | 1.65 | 0.015 | 0.086 |
| REACTOME_IKK_COMPLEX_RECRUITMENT_MEDIATED_BY_RIP1 | 18 | 0.67 | 1.65 | 0.000 | 0.085 |
| REACTOME_APOPTOTIC_EXECUTION_PHASE | 51 | 0.52 | 1.65 | 0.002 | 0.084 |
| REACTOME_ANTIGEN_PROCESSING_CROSS_PRESENTATION | 86 | 0.48 | 1.65 | 0.000 | 0.087 |
| REACTOME_THROMBIN_SIGNALLING_THROUGH_PROTEINASE_ACTIVATED_RECEPTORS_PARS | 31 | 0.58 | 1.64 | 0.007 | 0.090 |
| REACTOME_HEDGEHOG_LIGAND_BIOGENESIS | 61 | 0.50 | 1.64 | 0.004 | 0.092 |
| REACTOME_SIGNALING_BY_MODERATE_KINASE_ACTIVITY_BRAF_MUTANTS | 43 | 0.54 | 1.64 | 0.011 | 0.091 |
| BIOCARTA_CREB_PATHWAY | 21 | 0.63 | 1.63 | 0.009 | 0.097 |
| REACTOME_MTOR_SIGNALLING | 39 | 0.55 | 1.63 | 0.011 | 0.101 |
| REACTOME_RAB_GEFS_EXCHANGE_GTP_FOR_GDP_ON_RABS | 86 | 0.47 | 1.62 | 0.007 | 0.102 |
| KEGG_OXIDATIVE_PHOSPHORYLATION | 110 | 0.45 | 1.62 | 0.002 | 0.101 |
| REACTOME_G_PROTEIN_ACTIVATION | 28 | 0.58 | 1.62 | 0.016 | 0.103 |
| REACTOME_TRIF_TICAM1_MEDIATED_TLR4_SIGNALING | 89 | 0.47 | 1.62 | 0.000 | 0.102 |
| REACTOME_GLYCOGEN_METABOLISM | 24 | 0.59 | 1.62 | 0.011 | 0.105 |
| REACTOME_SYNAPTIC_ADHESION_LIKE_MOLECULES | 21 | 0.63 | 1.61 | 0.013 | 0.105 |
| PID_TRAIL_PATHWAY | 25 | 0.59 | 1.61 | 0.014 | 0.104 |
| REACTOME_RIP_MEDIATED_NFKB_ACTIVATION_VIA_ZBP1 | 17 | 0.65 | 1.61 | 0.020 | 0.103 |
| REACTOME_STABILIZATION_OF_P53 | 52 | 0.51 | 1.61 | 0.006 | 0.103 |
| REACTOME_ANTIMICROBIAL_PEPTIDES | 64 | 0.49 | 1.61 | 0.002 | 0.102 |
| REACTOME_G_PROTEIN_BETA:GAMMA_SIGNALLING | 32 | 0.57 | 1.61 | 0.015 | 0.101 |
| REACTOME_ASYMMETRIC_LOCALIZATION_OF_PCP_PROTEINS | 60 | 0.50 | 1.61 | 0.009 | 0.100 |
| REACTOME_ER_TO_GOLGI_ANTEROGRADE_TRANSPORT | 143 | 0.43 | 1.61 | 0.000 | 0.103 |
| PID_MTOR_4PATHWAY | 67 | 0.48 | 1.61 | 0.010 | 0.102 |
| KEGG_OOCYTE_MEIOSIS | 105 | 0.45 | 1.61 | 0.000 | 0.102 |
| REACTOME_ANTIGEN_PROCESSING:_UBIQUITINATION_PROTEASOME_DEGRADATION | 291 | 0.40 | 1.60 | 0.000 | 0.105 |
| REACTOME_PCP_CE_PATHWAY | 88 | 0.46 | 1.60 | 0.002 | 0.106 |
| KEGG_TERPENOID_BACKBONE_BIOSYNTHESIS | 15 | 0.66 | 1.59 | 0.030 | 0.111 |
| REACTOME_INTERLEUKIN_1_FAMILY_SIGNALING | 131 | 0.44 | 1.59 | 0.002 | 0.110 |
| REACTOME_BETA_CATENIN_INDEPENDENT_WNT_SIGNALING | 141 | 0.43 | 1.59 | 0.000 | 0.109 |
| REACTOME_TRAF6_MEDIATED_IRF7_ACTIVATION | 24 | 0.60 | 1.59 | 0.009 | 0.111 |
| REACTOME_COPI_MEDIATED_ANTEROGRADE_TRANSPORT | 94 | 0.46 | 1.59 | 0.000 | 0.110 |
| REACTOME_REGULATION_OF_RAS_BY_GAPS | 64 | 0.48 | 1.59 | 0.005 | 0.109 |
| PID_PLK1_PATHWAY | 44 | 0.52 | 1.59 | 0.009 | 0.111 |
| KEGG_PARKINSONS_DISEASE | 103 | 0.45 | 1.58 | 0.000 | 0.111 |
| PID_CDC42_PATHWAY | 69 | 0.47 | 1.58 | 0.006 | 0.110 |
| REACTOME_GLYCOLYSIS | 69 | 0.48 | 1.58 | 0.000 | 0.110 |
| REACTOME_G_BETA: GAMMA_SIGNALLING_THROUGH_CDC42 | 20 | 0.62 | 1.58 | 0.024 | 0.112 |
| REACTOME_INTRINSIC_PATHWAY_FOR_APOPTOSIS | 46 | 0.51 | 1.58 | 0.007 | 0.112 |
| REACTOME_ADP_SIGNALLING_THROUGH_P2Y_PURINOCEPTOR_12 | 22 | 0.61 | 1.58 | 0.022 | 0.114 |
| PID_ERBB1_DOWNSTREAM_PATHWAY | 103 | 0.45 | 1.57 | 0.004 | 0.114 |
| PID_HIF1_TFPATHWAY | 64 | 0.48 | 1.57 | 0.007 | 0.115 |
| REACTOME_CLASS_I_MHC_MEDIATED_ANTIGEN_PROCESSING_PRESENTATION | 342 | 0.39 | 1.57 | 0.000 | 0.116 |
| REACTOME_PURINE_CATABOLISM | 17 | 0.65 | 1.57 | 0.026 | 0.118 |
| REACTOME_CELLULAR_RESPONSE_TO_HYPOXIA | 70 | 0.47 | 1.57 | 0.004 | 0.118 |
| PID_LIS1_PATHWAY | 28 | 0.56 | 1.57 | 0.025 | 0.118 |
| REACTOME_CLASS_I_PEROXISOMAL_MEMBRANE_PROTEIN_IMPORT | 20 | 0.61 | 1.56 | 0.021 | 0.118 |
| REACTOME_REGULATION_OF_RUNX2_EXPRESSION_AND_ACTIVITY | 69 | 0.47 | 1.56 | 0.006 | 0.118 |
| REACTOME_SIGNALLING_TO_RAS | 19 | 0.64 | 1.56 | 0.020 | 0.117 |
| REACTOME_TOLL_LIKE_RECEPTOR_4_TLR4_CASCADE | 118 | 0.43 | 1.56 | 0.000 | 0.119 |
| REACTOME_ADP_SIGNALLING_THROUGH_P2Y_PURINOCEPTOR_1 | 24 | 0.57 | 1.56 | 0.015 | 0.119 |
| REACTOME_EPHB_MEDIATED_FORWARD_SIGNALING | 42 | 0.51 | 1.56 | 0.013 | 0.118 |
| REACTOME_INTERLEUKIN_37_SIGNALING | 20 | 0.61 | 1.56 | 0.035 | 0.117 |
| REACTOME_CELL_EXTRACELLULAR_MATRIX_INTERACTIONS | 18 | 0.62 | 1.56 | 0.038 | 0.118 |
| BIOCARTA_GH_PATHWAY | 26 | 0.57 | 1.55 | 0.027 | 0.122 |
| REACTOME_RAB_GERANYLGERANYLATION | 60 | 0.48 | 1.55 | 0.008 | 0.122 |
| REACTOME_REGULATION_OF_IFNA_SIGNALING | 21 | 0.59 | 1.55 | 0.038 | 0.123 |
| REACTOME_RESPIRATORY_ELECTRON_TRANSPORT_ATP_SYNTHESIS_BY_CHEMIOSMOTIC_COUPLING_AND_HEAT_PRODUCTION_BY_UNCOUPLING_PROTEINS | 104 | 0.43 | 1.55 | 0.004 | 0.123 |
| REACTOME_SIGNALING_BY_RHO_GTPASES | 406 | 0.37 | 1.54 | 0.000 | 0.131 |
| KEGG_INSULIN_SIGNALING_PATHWAY | 132 | 0.42 | 1.54 | 0.000 | 0.131 |
| REACTOME_AUF1_HNRNP_D0_BINDS_AND_DESTABILIZES_MRNA | 49 | 0.48 | 1.54 | 0.014 | 0.133 |
| REACTOME_EPH_EPHRIN_SIGNALING | 90 | 0.44 | 1.53 | 0.002 | 0.132 |
| REACTOME_CYCLIN_A_B1_B2_ASSOCIATED_EVENTS_DURING_G2_M_TRANSITION | 25 | 0.56 | 1.53 | 0.021 | 0.131 |
| REACTOME_ACTIVATION_OF_NF_KAPPAB_IN_B_CELLS | 63 | 0.46 | 1.53 | 0.005 | 0.135 |
| REACTOME_OLFACTORY_SIGNALING_PATHWAY | 280 | 0.38 | 1.53 | 0.000 | 0.135 |
| REACTOME_MITOTIC_G2_G2_M_PHASES | 189 | 0.40 | 1.52 | 0.002 | 0.140 |
| KEGG_PATHOGENIC_ESCHERICHIA_COLI_INFECTION | 52 | 0.48 | 1.52 | 0.018 | 0.139 |
| REACTOME_PROSTACYCLIN_SIGNALLING_THROUGH_PROSTACYCLIN_RECEPTOR | 19 | 0.60 | 1.52 | 0.038 | 0.139 |
| REACTOME_NITRIC_OXIDE_STIMULATES_GUANYLATE_CYCLASE | 21 | 0.60 | 1.52 | 0.022 | 0.138 |
| REACTOME_FCERI_MEDIATED_NF_KB_ACTIVATION | 76 | 0.45 | 1.52 | 0.006 | 0.138 |
| REACTOME_FCGAMMA_RECEPTOR_FCGR_DEPENDENT_PHAGOCYTOSIS | 83 | 0.45 | 1.52 | 0.005 | 0.137 |
| REACTOME_GLUCONEOGENESIS | 32 | 0.54 | 1.52 | 0.020 | 0.136 |
| PID_MET_PATHWAY | 78 | 0.45 | 1.52 | 0.009 | 0.139 |
| REACTOME_DIGESTION_AND_ABSORPTION | 22 | 0.57 | 1.52 | 0.027 | 0.138 |
| REACTOME_COPII_MEDIATED_VESICLE_TRANSPORT | 63 | 0.46 | 1.52 | 0.011 | 0.139 |
| REACTOME_INTERLEUKIN_1_SIGNALING | 95 | 0.43 | 1.52 | 0.009 | 0.138 |
| KEGG_LONG_TERM_POTENTIATION | 65 | 0.47 | 1.52 | 0.011 | 0.137 |
| REACTOME_SIGNALING_BY_BRAF_AND_RAF_FUSIONS | 63 | 0.47 | 1.51 | 0.006 | 0.138 |
| REACTOME_ACTIVATED_NOTCH1_TRANSMITS_SIGNAL_TO_THE_NUCLEUS | 28 | 0.54 | 1.51 | 0.028 | 0.143 |
| REACTOME_THE_ROLE_OF_GTSE1_IN_G2_M_PROGRESSION_AFTER_G2_CHECKPOINT | 70 | 0.46 | 1.51 | 0.020 | 0.143 |
| REACTOME_SIGNALING_BY_RAS_MUTANTS | 56 | 0.47 | 1.50 | 0.019 | 0.146 |
| REACTOME_HEDGEHOG_ON_STATE | 81 | 0.43 | 1.50 | 0.022 | 0.147 |
| REACTOME_SIGNALING_BY_NTRKS | 96 | 0.43 | 1.50 | 0.007 | 0.152 |
| REACTOME_DEGRADATION_OF_BETA_CATENIN_BY_THE_DESTRUCTION_COMPLEX | 80 | 0.43 | 1.50 | 0.013 | 0.152 |
| KEGG_PROGESTERONE_MEDIATED_OOCYTE_MATURATION | 80 | 0.44 | 1.49 | 0.011 | 0.153 |
| REACTOME_SIGNALING_BY_WNT | 286 | 0.37 | 1.49 | 0.000 | 0.153 |
| REACTOME_NICOTINATE_METABOLISM | 31 | 0.52 | 1.49 | 0.040 | 0.155 |
| KEGG_RIG_I_LIKE_RECEPTOR_SIGNALING_PATHWAY | 64 | 0.46 | 1.49 | 0.011 | 0.154 |
| REACTOME_MTORC1_MEDIATED_SIGNALLING | 22 | 0.55 | 1.49 | 0.041 | 0.157 |
| REACTOME_GLUCOSE_METABOLISM | 88 | 0.43 | 1.49 | 0.002 | 0.156 |
| SIG_PIP3_SIGNALING_IN_CARDIAC_MYOCTES | 65 | 0.45 | 1.48 | 0.020 | 0.164 |
| REACTOME_NEUROTRANSMITTER_RECEPTORS_AND_POSTSYNAPTIC_SIGNAL_TRANSMISSION | 195 | 0.39 | 1.47 | 0.002 | 0.176 |
| REACTOME_ACTIVATION_OF_NMDA_RECEPTORS_AND_POSTSYNAPTIC_EVENTS | 86 | 0.43 | 1.47 | 0.019 | 0.179 |
| REACTOME_RESPIRATORY_ELECTRON_TRANSPORT | 85 | 0.42 | 1.47 | 0.009 | 0.177 |
| KEGG_EPITHELIAL_CELL_SIGNALING_IN_HELICOBACTER_PYLORI_INFECTION | 65 | 0.45 | 1.46 | 0.017 | 0.184 |
| REACTOME_INSERTION_OF_TAIL_ANCHORED_PROTEINS_INTO_THE_ENDOPLASMIC_RETICULUM_MEMBRANE | 22 | 0.55 | 1.45 | 0.049 | 0.193 |
| REACTOME_PYRUVATE_METABOLISM | 30 | 0.51 | 1.45 | 0.036 | 0.194 |
| REACTOME_PTEN_REGULATION | 133 | 0.40 | 1.45 | 0.005 | 0.193 |
| REACTOME_REGULATION_OF_RUNX3_EXPRESSION_AND_ACTIVITY | 50 | 0.46 | 1.45 | 0.024 | 0.195 |
| REACTOME_THE_CITRIC_ACID_TCA_CYCLE_AND_RESPIRATORY_ELECTRON_TRANSPORT | 154 | 0.39 | 1.44 | 0.006 | 0.197 |
| REACTOME_CYCLIN_A:CDK2_ASSOCIATED_EVENTS_AT_S_PHASE_ENTRY | 81 | 0.43 | 1.44 | 0.018 | 0.196 |
| REACTOME_TOLL_LIKE_RECEPTOR_CASCADES | 141 | 0.39 | 1.44 | 0.011 | 0.194 |
| PID_ERBB1_RECEPTOR_PROXIMAL_PATHWAY | 34 | 0.50 | 1.44 | 0.044 | 0.195 |
| REACTOME_G1_S_DNA_DAMAGE_CHECKPOINTS | 63 | 0.45 | 1.44 | 0.025 | 0.194 |
| PID_INSULIN_GLUCOSE_PATHWAY | 26 | 0.53 | 1.44 | 0.048 | 0.195 |
| REACTOME_TOLL_LIKE_RECEPTOR_TLR1:TLR2_CASCADE | 90 | 0.42 | 1.44 | 0.025 | 0.194 |
| REACTOME_THE_ROLE_OF_NEF_IN_HIV_1_REPLICATION_AND_DISEASE_PATHOGENESIS | 26 | 0.53 | 1.44 | 0.047 | 0.193 |
| REACTOME_ACTIVATION_OF_GENE_EXPRESSION_BY_SREBF_SREBP | 42 | 0.48 | 1.44 | 0.029 | 0.193 |
| PID_LKB1_PATHWAY | 46 | 0.47 | 1.44 | 0.039 | 0.194 |
| REACTOME_ONCOGENIC_MAPK_SIGNALING | 79 | 0.42 | 1.43 | 0.022 | 0.198 |
| REACTOME_RHO_GTPASES_ACTIVATE_IQGAPS | 27 | 0.52 | 1.43 | 0.048 | 0.198 |
| PID_THROMBIN_PAR1_PATHWAY | 42 | 0.47 | 1.43 | 0.043 | 0.198 |
| KEGG_ADHERENS_JUNCTION | 72 | 0.43 | 1.43 | 0.023 | 0.198 |
| REACTOME_MAPK6_MAPK4_SIGNALING | 83 | 0.42 | 1.42 | 0.023 | 0.199 |
| BIOCARTA_BIOPEPTIDES_PATHWAY | 28 | 0.50 | 1.42 | 0.049 | 0.204 |
| REACTOME_TRANSPORT_TO_THE_GOLGI_AND_SUBSEQUENT_MODIFICATION | 172 | 0.38 | 1.42 | 0.007 | 0.203 |
| KEGG_ALZHEIMERS_DISEASE | 147 | 0.38 | 1.42 | 0.017 | 0.205 |
| REACTOME_RUNX1_REGULATES_TRANSCRIPTION_OF_GENES_INVOLVED_IN_DIFFERENTIATION_OF_HSCS | 89 | 0.41 | 1.41 | 0.021 | 0.207 |
| REACTOME_DOWNSTREAM_TCR_SIGNALING | 82 | 0.41 | 1.41 | 0.022 | 0.207 |
| REACTOME_INTRA_GOLGI_AND_RETROGRADE_GOLGI_TO_ER_TRAFFIC | 191 | 0.37 | 1.41 | 0.005 | 0.211 |
| REACTOME_REGULATION_OF_CHOLESTEROL_BIOSYNTHESIS_BY_SREBP_SREBF | 54 | 0.44 | 1.40 | 0.047 | 0.215 |
| REACTOME_PROTEIN_UBIQUITINATION | 64 | 0.42 | 1.40 | 0.038 | 0.212 |
| REACTOME_RHO_GTPASES_ACTIVATE_PKNS | 55 | 0.44 | 1.40 | 0.028 | 0.212 |
| REACTOME_CYTOSOLIC_SENSORS_OF_PATHOGEN_ASSOCIATED_DNA | 58 | 0.43 | 1.40 | 0.042 | 0.217 |
| REACTOME_TOLL_LIKE_RECEPTOR_9_TLR9_CASCADE | 88 | 0.41 | 1.39 | 0.021 | 0.229 |
| REACTOME_COMPLEX_I_BIOGENESIS | 48 | 0.44 | 1.38 | 0.048 | 0.233 |
| KEGG_NEUROTROPHIN_SIGNALING_PATHWAY | 121 | 0.38 | 1.38 | 0.018 | 0.232 |
| REACTOME_DOWNSTREAM_SIGNALING_EVENTS_OF_B_CELL_RECEPTOR_BCR | 76 | 0.41 | 1.38 | 0.047 | 0.233 |
| REACTOME_L1CAM_INTERACTIONS | 111 | 0.39 | 1.38 | 0.027 | 0.233 |
| REACTOME_SPHINGOLIPID_METABOLISM | 82 | 0.41 | 1.38 | 0.024 | 0.235 |
| KEGG_OLFACTORY_TRANSDUCTION | 278 | 0.34 | 1.37 | 0.009 | 0.243 |
| REACTOME_FC_EPSILON_RECEPTOR_FCERI_SIGNALING | 125 | 0.38 | 1.37 | 0.011 | 0.244 |
| KEGG_PEROXISOME | 77 | 0.40 | 1.37 | 0.032 | 0.246 |
| REACTOME_TCF_DEPENDENT_SIGNALING_IN_RESPONSE_TO_WNT | 191 | 0.36 | 1.37 | 0.011 | 0.245 |
| REACTOME_SPHINGOLIPID_DE_NOVO_BIOSYNTHESIS | 43 | 0.45 | 1.37 | 0.043 | 0.245 |
| REACTOME_SIGNALING_BY_NOTCH4 | 76 | 0.40 | 1.36 | 0.042 | 0.248 |
| REACTOME_MYD88_CASCADE_INITIATED_ON_PLASMA_MEMBRANE | 77 | 0.40 | 1.36 | 0.041 | 0.247 |
| KEGG_PHOSPHATIDYLINOSITOL_SIGNALING_SYSTEM | 74 | 0.40 | 1.36 | 0.049 | 0.249 |
| REACTOME_TCR_SIGNALING | 103 | 0.38 | 1.36 | 0.026 | 0.248 |
| REACTOME_SWITCHING_OF_ORIGINS_TO_A_POST_REPLICATIVE_STATE | 84 | 0.39 | 1.35 | 0.033 | 0.248 |
