# Supplementary material for: Clonal hematopoiesis with JAK2V617F promotes pulmonary hypertension with ALK1 upregulation in lung neutrophils
Source: Nat Commun. 2021 Oct 26;12:6177. doi: 10.1038/s41467-021-26435-0 (PMC8548396; doi:10.1038/s41467-021-26435-0)
Supplement: Supplementary file 5 — Reporting Summary [file 41467_2021_26435_MOESM5_ESM.pdf]

nature portfolio

Corresponding author(s): Tomofumi Misaka, Kazuhiko Ikeda

Last updated by author(s): Aug 25, 2021

Reporting Summary

Nature Portfolio wishes to improve the reproducibility of the work that we publish. This form provides structure for consistency and transparency in reporting. For further information on Nature Portfolio policies, see our Editorial Policies and the Editorial Policy Checklist.

Statistics

For all statistical analyses, confirm that the following items are present in the figure legend, table legend, main text, or Methods section.

n/a	Confirmed	
☐	☒	The exact sample size (*n*) for each experimental group/condition, given as a discrete number and unit of measurement
☐	☒	A statement on whether measurements were taken from distinct samples or whether the same sample was measured repeatedly
☐	☒	The statistical test(s) used AND whether they are one- or two-sided<br>*Only common tests should be described solely by name; describe more complex techniques in the Methods section.*
☒	☐	A description of all covariates tested
☐	☒	A description of any assumptions or corrections, such as tests of normality and adjustment for multiple comparisons
☐	☒	A full description of the statistical parameters including central tendency (e.g. means) or other basic estimates (e.g. regression coefficient) AND variation (e.g. standard deviation) or associated estimates of uncertainty (e.g. confidence intervals)
☐	☒	For null hypothesis testing, the test statistic (e.g. F, t, r) with confidence intervals, effect sizes, degrees of freedom and P value noted<br>*Give P values as exact values whenever suitable.*
☒	☐	For Bayesian analysis, information on the choice of priors and Markov chain Monte Carlo settings
☒	☐	For hierarchical and complex designs, identification of the appropriate level for tests and full reporting of outcomes
☒	☐	Estimates of effect sizes (e.g. Cohen's *d*, Pearson's *r*), indicating how they were calculated

Our web collection on statistics for biologists contains articles on many of the points above.

Software and code

Policy information about availability of computer code

Data collection	Sysmex pocH-100i<br>Keyence BZ-X700 and Keyence BZ II Viewer (version 01.03.01.01)<br>CFX Connect real-time PCR system<br>QuantStudio 3 real-time PCR system<br>ImageQuant LAS-4000 digital imaging system<br>Odyssey CLX imaging system<br>FACSCanto II<br>Hiseq2500

Data analysis	ImageJ ver 1.52a<br>Image Studio Lite version 5.2<br>LabScribe3<br>Bio-Rad CFX Manager 3.1<br>GraphPadPrism 8.1.2<br>TopHat2 version 2.0.13<br>Bowtie2 version 2.1.0<br>Flowjo v10.2<br>Ingenuity Pathways Analysis (IPATM) Winter Release (December2019) version<br>Gene Set Enrichment Analysis v1.4.3<br>QuantStudio Design and Analysis v1.4.3<br>Statistical Package for Social Sciences (SPSS) version 26

For manuscripts utilizing custom algorithms or software that are central to the research but not yet described in published literature, software must be made available to editors and reviewers. We strongly encourage code deposition in a community repository (e.g. GitHub). See the Nature Portfolio guidelines for submitting code & software for further information.

Data

Policy information about availability of data

All manuscripts must include a data availability statement. This statement should provide the following information, where applicable:

- Accession codes, unique identifiers, or web links for publicly available datasets
- A description of any restrictions on data availability
- For clinical datasets or third party data, please ensure that the statement adheres to our policy

The RNA sequencing data generated in this study have been deposited in the DNA Data Bank of Japan database under accession code DDBJ PRJDB9389 [https://ddbj.nig.ac.jp/BPSearch/bioproject?acc=PRJDB9389]. The putative STAT binding sites were assessed using JASPAR [http://jaspar.genereg.net/] and TFBIND/TRANSFAC [https://tfbind.hgc.jp/] databases.

Field-specific reporting

Please select the one below that is the best fit for your research. If you are not sure, read the appropriate sections before making your selection.

[x] Life sciences   [ ] Behavioural & social sciences   [ ] Ecological, evolutionary & environmental sciences

For a reference copy of the document with all sections, see nature.com/documents/nr-reporting-summary-flat.pdf

Life sciences study design

All studies must disclose on these points even when the disclosure is negative.

Sample size	Sample sizes were determined based on our studies and previous experimental experience with similar assays (eg. Ueda et. al Blood Adv 2017; 1: 1001–1015. Misaka et al. J Mol Cell Cardiol 2018; 114: 93-104) and referred to other similar reports (eg. Tu et al. Circ Res 2019; 124: 846–855).
Data exclusions	In case of mice, when the major bleeding was observed and/or the mice were dead unexpectedly due to the technical issues during the right heart catheterization, the data of the individual mice were excluded. No data were excluded from in vitro studies or human studies.
Replication	Each experiment was run in technical duplicates or triplicates, and the biological experiments were performed in independent replicates. All attempts at replication were successful. The experiments were usually conducted at least three times independently to ensure reproducibility of the data and for statistical analysis. Individual values are shown in each figure.
Randomization	Mice were matched into groups according to the genotype, age and body weight, and then the mice were allocated and selected randomly. The investigators were blinded to group allocation during data collection using the mouse identification number, which did not specify the allocated group. In vitro studies, samples were randomly allocated into the different treatment group.
Blinding	During data collection and sample analysis using analysis equipment, the investigators were blinded to the mouse genotype, the group allocation, clinical backgrounds using the numerical code, which did not specify the allocated group. When the mice were treated with the specific chemicals, the investigators were not blinded because the drugs had to be administrated to the same mouse, but the mice and samples were eventually blinded using the numerical code when analyzing the data (eg. right ventricular systolic pressure, histological analysis for quantification).

Reporting for specific materials, systems and methods

We require information from authors about some types of materials, experimental systems and methods used in many studies. Here, indicate whether each material, system or method listed is relevant to your study. If you are not sure if a list item applies to your research, read the appropriate section before selecting a response.

Materials & experimental systems

n/a	Involved in the study	
☐	☒	Antibodies
☐	☒	Eukaryotic cell lines
☒	☐	Palaeontology and archaeology
☐	☒	Animals and other organisms
☐	☒	Human research participants
☒	☐	Clinical data
☒	☐	Dual use research of concern

Methods

n/a	Involved in the study	
☒	☐	ChIP-seq
☐	☒	Flow cytometry
☒	☐	MRI-based neuroimaging

Antibodies

Antibodies used

Primary antibodies:
a-smooth muscle actin (M0851, Dako; 19245, Cell Signaling Technology), Ly6G (ab25377, Abcam), F4/80 (70076, Cell Signaling Technology), CD45 (70257, Cell Signaling Technology; sc-53665, Santa Cruz Biotechnology Inc.), CD45R (103201, Biolegend), Ki67 (ab15580, Abcam; NB600-1252, Novus Biologicals), CD41 (ab63983, Abcam), TER-119 (116201, BioLegend), GFP (NBP2-22111, Novus Biologicals), Myeloperoxidase (ab9535, Abcam), CD31 (102401, BioLegend), HIF1a (36169, Cell Signaling Technology), Phospho-STAT3 (9145, Cell Signaling Technology), STAT3 (4904, Cell Signaling Technology), Phospho-Smad1/Smad5/Smad8 (AB3848-I, Merck Millipore), Smad1 (9743, Cell Signaling Technology), ALK1 (14745-1-AP, Proteintech), ALK2 (MAB637, R&D Systems), GAPDH (60004-1-Ig, Proteintech), Ly6G MicroBeads (130-120-337, Miltenyi Biotec GmbH), CD117 (130-091-224, Miltenyi Biotec GmbH), CD31 MicroBeads (130-097-418, Miltenyi Biotec GmbH), CD45.2 (109814, BioLegend), Ly6G (560599, BD Biosciences).
Secondary antibodies:
Histofine Simple Stain Mouse MAX PO (R) (414341F, Nichirei Bioscience), Histofine MOUSESTAIN KIT (414321F, Nichirei Bioscience), Goat anti-mouse horseradish peroxidase-conjugated secondary antibody (m-IgGκ BP-HRP, sc-516102, Santa Cruz Biotechnology Inc.), mouse anti-rabbit horseradish peroxidase-conjugated secondary antibody (sc-2357, Santa Cruz Biotechnology Inc.), IRDye 680RD Goat anti-Rabbit IgG (925-68071, LI-COR, Inc), IRDye 680RD Goat anti-Mouse IgG (925-68070, LI-COR, Inc), IRDye 800CW Donkey anti-Rabbit IgG (925-32213, LI-COR, Inc), IRDye 800CW Goat anti-Mouse IgG 925-32210, LI-COR, Inc), Donkey Anti-Mouse IgG H&L (Alexa Fluor® 488) (ab150105, Abcam), Donkey anti-Rabbit IgG (H+L) ReadyProbes™ Secondary Antibody, Alexa Fluor 594 (R37119, Thermo Fisher Scientific), Rabbit anti-Rat IgG (H+L) Cross-Adsorbed Secondary Antibody, Alexa Fluor 594 (A-21211, Thermo Fisher Scientific), Goat Anti-Rat IgG H&L (Alexa Fluor® 647) (ab150159, Abcam).

Validation

As per the manufacturer's product information, mouse anti-a-smooth muscle actin antibody (ClonelA, 4M0851, Dako) and rabbit anti-a-smooth muscle actin antibody (19245, Cell Signaling Technology) detect endogenous a-smooth muscle actin by immunostaining of frozen and paraffin tissue sections, and are validated using isolated mouse smooth muscle cells in house. As per the manufacturer's product information, rat anti-Ly6G antibody (ab25377, Abcam) detects endogenous mouse Ly6G by immunostaining of frozen and paraffin tissue sections.
As per the manufacturer's product information, rabbit anti-F4/80 antibody (70076, Cell Signaling Technology) detects endogenous mouse F4/80 by immunostaining of frozen and paraffin tissue sections.
As per the manufacturer's product information, rabbit anti-CD45 antibody (70257, Cell Signaling Technology) or rat anti-CD45 antibody (se-53665, Santa Cruz Biotechnology Inc.) detects endogenous mouse CD45 by immunostaining of frozen and paraffin tissue sections.
As per the manufacturer's product information, rat anti-CD45R antibody (103201, Biolegend) detects endogenous mouse CD45R by immunostaining of frozen and paraffin tissue sections.
As per the manufacturer's product information, rabbit anti-CD41 antibody (ab63983, Abcam) detects endogenous mouse CD41 by immunostaining of paraffin tissue sections.
As per the manufacturer's product information, rabbit or mouse anti-Ki67 antibody (ab15580, Abcam; NB600-1252, Novus Biologicals) detects mouse Ki67 by immunofluorescence and immunohistochemistry of frozen and paraffin tissue sections.
As per the manufacturer's product information, rat anti-TER-119 antibody (116201, BioLegend) detects endogenous mouse TER-119 by immunostaining of frozen and paraffin tissue sections.
As per the manufacturer's product information, mouse anti-GFP antibody (NBP2-22111, Novus Biologicals) detects GFP by immunostaining of frozen and paraffin tissue sections.
As per the manufacturer's product information, mouse anti-Myeloperoxidase antibody (ab9535, Abcam) detects Myeloperoxidase by immunofluorescence and is validated using isolated mouse neutrophils in house.
As per the manufacturer's product information, rat anti-CD31 antibody (102401, BioLegend) detects mouse CD31 by immunofluorescent staining is validated using isolated mouse endothelial cells.
As per the manufacturer's product information, rabbit anti-HIF1a antibody (36169, Cell Signaling Technology) detects endogenous levels of total HIF1a protein of mouse by Western blot and used for Immunoprecipitation.
As per the manufacturer's product information, rabbit anti-Phospho-STAT3 antibody (9145, Cell Signaling Technology) detects endogenous levels of STAT3 of mouse and human only when phosphorylated at tyrosine 705 by Western blot.
As per the manufacturer's product information, rabbit anti-STAT3 antibody (4904, Cell Signaling Technology) detects endogenous levels of total STAT3 protein of mouse and human by Western blot and used for chromatin Immunoprecipitation.
As per the manufacturer's product information, rabbit anti-Phospho-Smad1/Smad5/Smad8 antibody (AB3848-I, Merck Millipore) detects endogenous levels of Smad1/Smad5/Smad8 of mouse and human only when dually phosphorylated at Ser463 and Ser465, as well as phosphorylated Smad5 and Smad8 at the equivalent sites by Western blot.
As per the manufacturer's product information, rabbit anti-Smad1 antibody (9743, Cell Signaling Technology) detects endogenous levels of total Smad1 protein of mouse and human by Western blot.
As per the manufacturer's product information, rabbit anti-ALK1 antibody (14745-1-AP, Proteintech) detects endogenous levels of total ALK1 protein of mouse and human by Western blot and flow cytometry. The decreases in ALK1 protein levels were verified by

knockdown with siRNA.
As per the manufacturer's product information, mouse anti-ALK2 antibody (MAB637, R&D Systems) detects endogenous levels of ALK2 of human by Western blot
As per the manufacturer's product information, mouse anti-GAPDH (60004-1-Ig, Proteintech) detects endogenous levels of GAPDH of mouse and human by Western blot.
Anti-Ly6G MicroBeads (130-120-337, Miltenyi Biotec GmbH) was used for the positive selection of mouse neutrophils. The purity of the neutrophils was >98% as determined by May-Giemsa staining and was confirmed with the positive immunostaining by anti-Ly6G and anti-Myeloperoxidase antibodies and with the negative immunostaining by an anti-CD31 antibody.
Anti-CD117 MicroBeads (130-091-224, Miltenyi Biotec GmbH) was used for the positive selection of mouse hematopoietic stem and progenitor cells. The sored cells from mouse bone marrow using CD117-MACS were used for positive controls. On the manufacturer's website, the purity of the CD117+ cells validated by flow cytometry or fluorescence microscopy.
Anti-CD31 MicroBeads (130-097-418, Miltenyi Biotec GmbH) was used for the positive selection of endothelial cells from single-cell suspensions of mouse tissues. The sored cells were confirmed with the positive immunostaining by an anti-CD31 antibody. On the manufacturer's website, the purity of the CD31+ cells validated by flow cytometry or fluorescence microscopy.
As per the manufacturer's product information, APC anti-mouse CD45.2 antibody (109814, BioLegend) detects the endogenous expression of Ly5.2 bearing C57BL6 mice and is used for flow cytometry.
As per the manufacturer's product information, APC Rat anti-mouse Ly6G antibody (109814, BioLegend) detects endogenous expression of Ly6G of mouse and is used for flow cytometry.

As per the manufacturer's product information, Histofine Simple Stain Mouse MAX PO (R) (414341F, Nichirei Bioscience) has been validated for use in immunohistochemical staining on formalin-fixed paraffin-embedded mouse tissue sections as a secondary antibody for use with Rabbit primary antibody.
As per the manufacturer's product information, Histofine Simple Stain Mouse MAX PO (Rat) (414311F, Nichirei Bioscience) has been validated for use in immunohistochemical staining on formalin-fixed paraffin-embedded mouse tissue sections as a secondary antibody for use with Rat primary antibody.
As per the manufacturer's product information, goat anti-mouse horseradish peroxidase-conjugated secondary antibody (m-IgGκ BP-HRP, sc-516102, Santa Cruz Biotechnology Inc.) has been validated for use in westernblotting as a secondary antibody for use with Mouse primary antibody.
As per the manufacturer's product information, mouse anti-rabbit horseradish peroxidase-conjugated secondary antibodies (sc-2357, Santa Cruz Biotechnology Inc.) has been validated for use in westernblotting as a secondary antibody for use with Rabbit primary antibody.
As per the manufacturer's product information, IRDye 680RD Goat anti-Rabbit IgG (925-68071, LI-COR, Inc), IRDye 680RD Goat anti-Mouse IgG (925-68070, LI-COR, Inc), IRDye 800CW Donkey anti-Rabbit IgG (925-32213, LI-COR, Inc), and IRDye 800CW Goat anti-Mouse IgG 925-32210, LI-COR, Inc) have been validated for use in westernblotting as secondary antibodies.
As per the manufacturer's product information, donkey Anti-Mouse IgG H&L (Alexa Fluor® 488) (ab150105, Abcam) has been validated for use in immunostaining as a secondary antibody.
As per the manufacturer's product information, donkey anti-Rabbit IgG (H+L) ReadyProbes™ Secondary Antibody Alexa Fluor 594 (R37119, Thermo Fisher Scientific) has been validated for use in immunostaining as a secondary antibody for use with Rabbit primary antibody.
As per the manufacturer's product information, Rabbit anti-Rat IgG (H+L) Cross-Adsorbed Secondary Antibody Alexa Fluor 594 (A-21211, Thermo Fisher Scientific) has been validated for use in immunostaining as a secondary antibody for use with Rat primary antibody.
As per the manufacturer's product information, Goat Anti-Rat IgG H&L (Alexa Fluor® 647) (ab150159, Abcam) has been validated for use in immunostaining as a secondary antibody for use with Rat primary antibody.

Eukaryotic cell lines

Policy information about cell lines

Cell line source(s)	JAK2V617F/+ knock-in HCT116 cells were purchased from Horizon Discovery Ltd (Catalog No. HD 104-025, Serial No. 01489). JAK2+/+ HCT116 cells were provided together and used as control wild-type (JAK2+/+) from the same company (Catalog No. HD PAR-073, Serial No. 35494).
Authentication	JAK2V617F/+ knock-in HCT116 cells were authorized by Sanger sequence to confirm the presence of knock-in. Authentication of the HCT116 cells was described on the Horizon Discovery Ltd web site: https://horizondiscovery.com/en/engineered-cell-lines/products/cancer-cell-lines?nodeid=entrezgene-3717&catalognumber=HD%20104-025
Mycoplasma contamination	Mycoplasma testing was negative.
Commonly misidentified lines (See ICLAC register)	None

Animals and other organisms

Policy information about studies involving animals; ARRIVE guidelines recommended for reporting animal research

Laboratory animals	The JAK2V617F mice of transgenic-Jak2V617F with a C57BL/6J background were obtained as described in the previous literature (Leukemia 2008; 22: 87-95. Blood Adv 2017; 1: 1001-1015). The female JAK2V617F mice aged between 8 and 10 weeks (body weight range was from 18 to 24 g) were used in the present study otherwise indicated. Wild-type littermates were used as controls. We characterized further JAK2V617F mice using male JAK2V617F mice aged between 8 and 10 weeks and aged female JAK2V617F mice

at 8- to 9-month-old. Female C57BL/6J mice aged between 8 and 10 weeks as recipients were obtained from charles river laboratories Japan. CAG-EGFP reporter mice with a C57BL/6J background were purchased from Japan SLC. The JAK2V617F mice were crossed with CAG-EGFP mice to generate JAK2V617F/CAG-EGFP double transgenic mice (JAK2V617F-GFP). WT littermates were used as controls (WT¬GFP).

Wild animals	The study did not involve wild animals.
Field-collected samples	The study did not involve samples collected from the field.
Ethics oversight	All animal studies were reviewed and approved by the Fukushima Medical University Animal Research Committee (approval number; 2019084).

Note that full information on the approval of the study protocol must also be provided in the manuscript.

Human research participants

Policy information about studies involving human research participants

Population characteristics	This study recruited the patients with pulmonary hypertension at Fukushima Medical University hospital between April 2018 and April 2020, and blood samples were collected after obtaining the informed consent. For the control group, we also recruited healthy volunteers by request or poster advertisement and the patients who had never developed the symptoms of pulmonary hypertension or without history of cardiopulmonary diseases during the same periods at the same hospital. The covariate-relevant population characteristics of the human research participants such as age, gender were described in Supplementary Table 1.
Recruitment	The patients were recruited consecutively at hospitalization and outpatient-clinic at the same hospital during the periods. For the control group, we recruited healthy volunteers by request or poster advertisement and the patients who had never developed the symptoms of pulmonary hypertension or without history of cardiopulmonary diseases during the same periods at the same hospital. After the written informed consent was obtained from each patient or volunteer, the blood samples were collected and registered in the present study. The persons who did not agree with the study consent after explanation about the contents were not included for this study. Given that PH patients were recruited consecutively at a single center and only one PH patient did not agree with the study participation, a self-selection bias would not critically be introduced or would not impact the results.
Ethics oversight	The protocol of the human studies was approved by the institutional ethics committee of Fukushima Medical University Hospital (approval number; 29348) and confirmed in accordance with the ethical guidelines of the 1975 Declaration of Helsinki.

Note that full information on the approval of the study protocol must also be provided in the manuscript.

Flow Cytometry

Plots

Confirm that:

- [x] The axis labels state the marker and fluorochrome used (e.g. CD4-FITC).
- [x] The axis scales are clearly visible. Include numbers along axes only for bottom left plot of group (a 'group' is an analysis of identical markers).
- [x] All plots are contour plots with outliers or pseudocolor plots.
- [x] A numerical value for number of cells or percentage (with statistics) is provided.

Methodology

Sample preparation	Leukocytes were isolated from the peripheral blood and the lungs. The single cell suspensions from the lung tissues were prepared by the same methods described in MACS. To form a cell suspension from the lungs, the tissues were minced and digested in 2 mg/mL collagenase type II (Worthington Biochemical) for 30 min. Then the tissues were passed through an 18-gauge needle and a 70 um cell strainer.<br>After lysing red blood cells, the cells were washed with PBS and incubated with the relevant antibodies (CD45, 1:100 dilution; Ly6G, 1:50 dilution) for 30 min at 4 degree. After centrifuge at 2500 rpm for 5 min, the cells were resuspended with 1mL PBS, and then subjected to the flow cytometry by FACSCanto II.<br>HCT116 cells were trypsinized and collected as a single cell suspension, and then 1x10^5 cells were incubated with an anti-ALK1 antibody (14745-1-AP, Proteintech, 1:50 dilution) for 30 min at 4 degree. After centrifuge at 400 g for 5 min, the cells were incubated with R-PE-conjugated donkey anti-rabbit secondary antibody (Jackson ImmunoResearch, 1:100 dilution) for 30 min at room temperature, and then after centrifuge at 400 g for 5 min, the cells were resuspended with FACS buffer and subjected to the flow cytometry by FACSCanto II.
Instrument	All data were collected using an FACSCanto II (BD Biosciences).
Software	All data were analyzed with FlowJoTM 10 software (version 10.2; Treestar, Ashland, OR).
Cell population abundance	In the post-cell fraction excluding the debris including red blood cells based on the FSC/SSC gating strategy, the cell abundance was > 20-60%. Sorted cell abundance was approximately 20% for CD45 or Ly6G expression.

Gating strategy

FSC/SSC were gated based on the cell sizes comparing to a standard beads. Positives were determined by comparing the controls of the unstained samples and the samples stained by an isotype control antibody. A sample gating strategy is provided in supplementary figure.

[x] Tick this box to confirm that a figure exemplifying the gating strategy is provided in the Supplementary Information.
